# The Finnegan Score for Neonatal Opioid Withdrawal Revisited With Routine Electronic Data: Retrospective Study

**DOI:** 10.2196/50575

**Published:** 2024-02-28

**Authors:** Till Rech, Kerstin Rubarth, Christoph Bührer, Felix Balzer, Christof Dame

**Affiliations:** 1 Department of Neonatology, Charité - Universitätsmedizin Berlin, Berlin, Germany; 2 Institute of Medical Informatics, Charité - Universitätsmedizin Berlin, Berlin, Germany

**Keywords:** data science application, neonatology, Finnegan score, neonatal opioid withdrawal syndrome, mixed models, neonate, neonatal, abstinence, opioid, withdrawal, substance abuse, postnatal, pediatrics, electronic health record, EHR, monitoring, health record, finnegan, neonatal abstinence syndrome, NAS, opioid withdrawal

## Abstract

**Background:**

The severity of neonatal abstinence syndrome (NAS) may be assessed with the Finnegan score (FS). Since the FS is laborious and subjective, alternative ways of assessment may improve quality of care.

**Objective:**

In this pilot study, we examined associations between the FS and routine monitoring data obtained from the electronic health record system.

**Methods:**

The study included 205 neonates with NAS after intrauterine (n=23) or postnatal opioid exposure (n=182). Routine monitoring data were analyzed at 60±10 minutes (t–1) and 120±10 minutes (t–2) before each FS assessment. Within each time period, the mean for each variable was calculated. Readings were also normalized to individual baseline data for each patient and parameter. Mixed effects models were used to assess the effect of different variables.

**Results:**

Plots of vital parameters against the FS showed heavily scattered data. When controlling for several variables, the best-performing mixed effects model displayed significant effects of individual baseline-controlled mean heart rate (estimate 0.04, 95% CI 0.02‐0.07) and arterial blood pressure (estimate 0.05, 95% CI 0.01‐0.08) at t–1 with a goodness of fit (*R*^2^_m_) of 0.11.

**Conclusions:**

Routine electronic data can be extracted and analyzed for their correlation with FS data. Mixed effects models show small but significant effects after normalizing vital parameters to individual baselines.

## Introduction

When exposure to opioids ends, neonates may develop withdrawal symptoms [1]. Neonatal abstinence syndrome (NAS), also referred to as *neonatal opioid withdrawal syndrome*, can be subdivided into primary NAS due to prenatal opioid abuse by (or treatment of) the mother, and iatrogenic NAS (iNAS) when neonates are treated with opioids. Primary NAS may develop in more than 90% of infants after intrauterine opiate exposure [2]. Occurrence and severity vary interindividually and are influenced by several factors, such as prematurity [3], breastfeeding [4], and multisubstance exposure, which results in more severe symptoms and worse outcomes than exclusive exposure to a single substance [5]. Though primary NAS is typically understood as abstinence from opioids, neonates can also develop withdrawal symptoms after exposure to other substances or medication such as tobacco [6], alcohol [7], cocaine [8], selective serotonin reuptake inhibitors and other antidepressants [9], benzodiazepines [10], and a combination of opioids and other substances [5].

Many neonatal intensive care units monitor withdrawal symptoms using the Neonatal Narcotic Abstinence Scoring System, also called the *Finnegan score* (FS), which is composed of 32 clinical signs, each scored between 0 and 5 (maximum score 46) [11]. The FS was originally designed to assess withdrawal in otherwise healthy-term infants from mothers abusing opioids [11,12]. Thus, the validity of the FS in other patients receiving neonatal intensive care is unclear, particularly in preterm or term neonates experiencing iNAS [11,12].

The implementation of electronic patient data management systems (PDMSs) alongside the availability of digital data on vital signs allows using data science algorithms to reevaluate clinical scoring systems and to facilitate clinical decision-making using decision support algorithms [13]. Having provided the first examples in adult medicine, these methods have shown promising results in neonatology. For instance, algorithmic analysis of heart rate characteristics is used to generate the Heart Rate Observation score—an estimate of the risk of developing sepsis [14]. Other approaches for early detection of sepsis use more variables and extensive machine learning algorithms but have not yet been validated in prospective settings [15]. Other studies have attempted to use data science algorithms to predict neonatal mortality [16]. Regarding opioid exposure, the PoPPI (Procedural Pain in Premature Infants) trial assessed the possibility of minimizing procedural pain in neonates receiving morphine treatment [17]. These data have allowed for the successful establishment of models predicting whether cardiorespiratory instability occurs after morphine administration and whether it requires intensified treatment [17,18].

Considering the subjectivity and the effort in generating an FS, the exploration of data-driven alternative ways of monitoring withdrawal symptoms appears necessary. In this pilot trial, we analyzed the association between electronic health data—mostly continuously and routinely monitored vital parameters—and the FS as a measure of the severity of NAS. Strong associations would allow an objective and less laborious NAS assessment based on routinely available data.

## Methods

### Ethical Considerations

The institutional review board of the Charité – Universitätsmedizin Berlin approved the study (EA2/104/21). Due to the retrospective nature of the study, the need for patient consent was waived.

### Data Export and Inclusion and Exclusion Criteria

Continuously and routinely monitored vital parameters were extracted from the electronic health systems and harmonized for further analysis.

Data were exported for all patients admitted to the clinic between January 1, 2013, and February 1, 2022. The data set was then refined on the basis of the following inclusion criteria, and all calculations were later performed within the refined data set. To include all patients with continued clinical suspicions of withdrawal symptoms but to exclude those with one-time-only suspicions or accidental documentation, we performed the export by selecting patients with at least 3 documented FSs, since a pharmaceutical intervention was usually not initiated on the basis of a single scoring result. We cross-referenced this export selection with patients classified as having NAS in accordance with *ICD-10* (*International Statistical Classification of Diseases, Tenth Revision*) criteria for quality control purposes. We categorized patients into subgroups of primary NAS and iNAS based on opioid medication, history of surgery, and time after birth before documenting the first FS for each patient.

Primary NAS was coded when at least 1 FS was documented before any opioid medication was administered, any surgery was performed, and the patient had not yet approached postpartum day 8. iNAS was coded when any opioid medication was administered before the patient’s first FS regardless of postnatal age. Patients with documented FS who did not meet any of these criteria were excluded. To design a sensitivity analysis, the analytic code was also applied to total study population without exclusion due to unclear NAS classification.

### Review of Hospital Data Structure

Each variable was checked for availability within the hospital information system (SAP/Cerner) as well as the PDMS (COPRA) used in the neonatal wards (levels 1‐3).

### Medication Data

Medications were not named consistently; hence, their names had to be preprocessed manually. We exported all unique medication entries from the PDMS and categorized them manually. The complete list of medication categories is provided in Multimedia Appendix 1.

### Variables

To evaluate patients’ demographics, we recorded their sex, gestational age at birth, birth weight, mode of delivery, number of documented FSs, and whether a time frame for individual baseline calculation was available and, if so, whether data were available within this time frame for the abovementioned vital parameters (including heart rate, respiratory rate, peripheral oxygen saturation, and mean blood pressure).

We calculated means for all variables listed below within specified time periods (t–1 and t–2, see the *Time Periods* section): heart rate, respiratory rate, peripheral oxygen saturation, and blood pressure.

**Figure 1.**
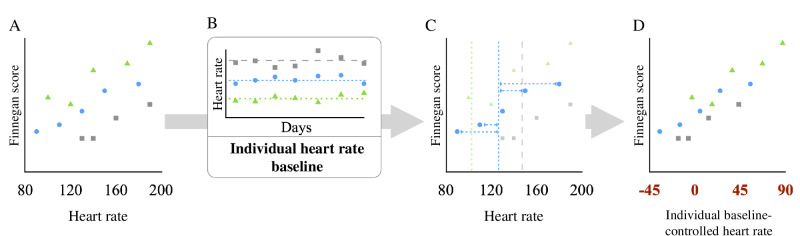
Schematic representation of the calculation of an individual baseline-controlled mean of a given vital parameter—heart rate. To reduce the scattering of data in (**A**), we calculated the mean of the vital parameter for each patient (denoted with a green triangle, a blue dot, and a gray square) during the individual baseline period (**B**). The definition of this period is illustrated in Figure 2. We then calculated the difference from the baseline for each vital parameter of each patient, as shown in (**C**). When plotting this difference (**D**), we obtained individual baseline-controlled means for vital parameters plotted on a scale around zero and with a more linear grouping of all measurements, irrespective of patient identity.

**Figure 2.**
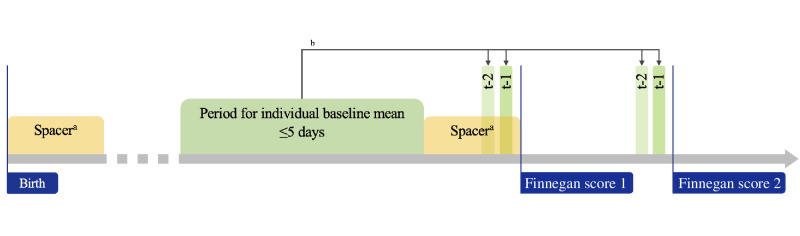
Time periods for the calculation of individual baselines (timeline not to scale). We defined a period to calculate an individual baseline for each patient of up to 5 consecutive days. After birth and before documenting the first Finnegan score, we introduced a spacer. ^a^The spacer has a length of 1 day for patients with primary neonatal abstinence syndrome (NAS) and 3 days for those with iatrogenic NAS. ^b^For all measurements within this period, we calculated a mean that is used to baseline-control measurements during every t–1 and t–2 for the respective patient; this baseline-control approach is illustrated in Figure 1.

Additionally, we generated an individual baseline for each patient by calculating the mean for each variable in a period of up to 5 days before documenting the first FS, which we defined as the relevant beginning of withdrawal (Figure 1). When calculating the individual baseline, we excluded spacer periods immediately post partum to minimize effects from postnatal adaptation and those immediately before documenting the first FS to minimize the effects of early-onset withdrawal. We set this spacer period to 1 day for patients with primary NAS and 3 days for those with iNAS (Figure 2). The sensitivity analysis was carried out with both spacer periods for the whole collective. We introduced the difference between this individual baseline and the mean of the respective vital parameter within the specified period before documenting any FS as a new variable to use as an alternative to the mean of the vital parameters and henceforth referred to this variable as the “baseline-controlled mean” (Figures1  2).

Body temperature was assessed in variable patterns, and we calculated the mean and baseline-controlled mean body temperature within 1 day before documenting each FS and used these data for both time periods (t–1 and t–2).

Furthermore, we included the following variables. (1) The pharmacodynamics of buprenorphine—the first-line pharmacotherapy for NAS—is highly complex, and data on transferability to neonates are limited [19,20]. Hence, we did not attempt to estimate pharmacodynamics in neonates but considered the time between the last documented opioid medication and each FS instead. As these *hours since medication* can only be recorded for patients who have been administered any opioids before documenting the respective FS, either because of iNAS or because of treatment of any type of NAS, we used the date of birth as the date of the last opioid medication for infants with primary NAS if no opioid medication was documented more recently. (2) The last body weight measure before documenting each FS was considered as a percentage of the individual’s birth weight. (3) The current gestational age at each FS documentation was recorded.

### Time Periods

All graphs and models were created for 2 time periods. With the goal of exploring options for predicting withdrawal symptoms, we focused on time periods before each FS. The first time period, t–1, was set to 1 hour±10 minutes before each FS, resulting in a 20-minute period from 10 minutes before until 10 minutes after the time point of 1 hour prior of each FS documentation. The second time period was set in the same manner to 2 hours±10 minutes before the respective FS, resulting in an earlier time period, t–2. The time periods are visualized in Figure 2.

### Data Analysis Software

Data analysis was carried out using RStudio (version 2022.07.1+554) and R (version 4.2.1; 2022-06-23 ucrt) [21,22] using the following packages and their dependencies in addition to the function included in R, RStudio, and the ::base-package R during data extraction and harmonization: cli [23], data.table [24], dplyr [25], lubridate [26], tibble [27], and tidyverse [28].

**Figure 3.**
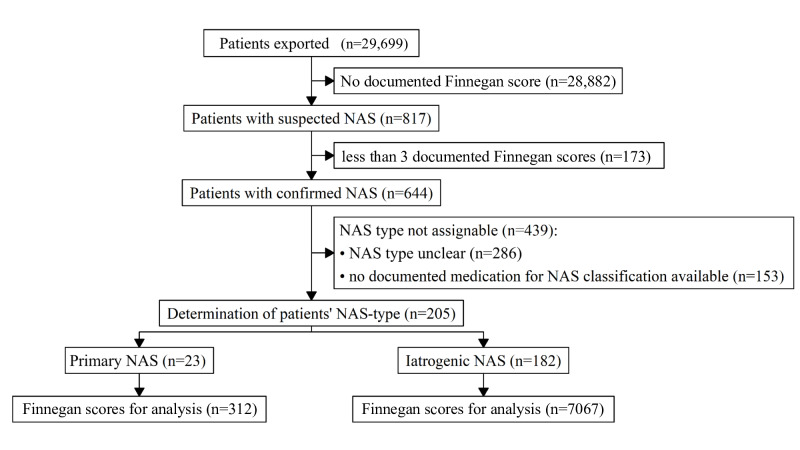
Patient allocation and numbers. NAS: neonatal abstinence syndrome.

We used the consort package to generate Figure 3 [29]. Table 1 was created using the tableone package; significance was tested using chi-square tests for categorical variables, Wilcoxon tests for skewed variables and *t* tests for normally distributed metric variables [30]. Skewness was assessed using the summary-function from tableone in accordance with tableone documentation [30]. We generated graphs with ggplot2 [31] and fitted our mixed effects models using lme4 [32]. Goodness of fit parameters of the mixed effects models was calculated using MuMIn [33]. Table output from RStudio was facilitated using flextable [34]. All code is has been published previously [35].

**Table 1.** Patient characteristics^a^.

Characteristics		Total	Primary NAS^b^	Iatrogenic NAS	*P* value
Participants, n		205	23	182	N/A^c^
**Sex, n (%)**					>.99^d^
	Female	72 (49.7)	8 (47.1)	64 (50.0)	
	Male	73 (50.3)	9 (52.9)	64 (50.0)	
Median gestational age at birth in weeks (IQR)		37+1 (32+4 to 39+1)	38+4 (37+1.75 to 40+1.5)	37+0 (31+5 to 39+1)	.02^e^
Birth weight (g), median (IQR)		2663 (1758-3243)	3140 (2583-3366)	2585 (1695-3200)	.01^e^
**Mode of delivery, n (%)**					<.001^d^
	Cesarean section	114 (55.6)	3 (13.0)	111 (61.0)	
	Vaginal delivery	74 (36.1)	19 (82.6)	55 (30.2)	
	Data not available	17 (8.3)	1 (4.3)	16 (8.8)	
Median number of documented Finnegan scores (IQR)		23 (10-47)	7 (5-14)	26 (12-50.75)	<.001^e^
**Time frame for individual baseline, n (%)**					.004^d^
	Definable	172 (83.9)	14 (60.9)	158 (86.8)	
	Not definable	33 (16.1)	9 (39.1)	24 (13.2)	
**Individual baseline data for heart rate, n (%)**					.005^d^
	Available	166 (81.0)	14 (60.9)	152 (83.5)	
	Not available^f^	6 (2.9)	0 (0)	6 (3.3)	
**Individual baseline data for respiratory rate, n (%)**					.005^d^
	Available	164 (80.0)	14 (60.9)	150 (82.4)	
	Not available^f^	8 (3.9)	0 (0)	8 (4.4)	
**Individual baseline data for peripheral oxygen saturation, n (%)**					.005^d^
	Available	165 (80.5)	14 (60.9)	151 (83.0)	
	Not available^f^	7 (3.4)	0 (0)	7 (3.8)	
**Individual baseline data for mean blood pressure, n (%)**					.004^d^
	Available	162 (79.0)	14 (60.9)	148 (81.3)	
	Not available^f^	10 (4.9)	0 (0)	10 (5.5)	

a All values rounded to integers except for pH.

b NAS: neonatal abstinence syndrome.

c N/A: not applicable.

d Categorial variables were assessed using the chi-square-test.

e Metric variables were assessed using the Wilcoxon–Mann-Whitney *U* test.

f Number of patients, for which a time interval for individual baseline calculation was definable but no data for the respective vital parameters were available within this interval.

### Graphs

**Figure 4.**
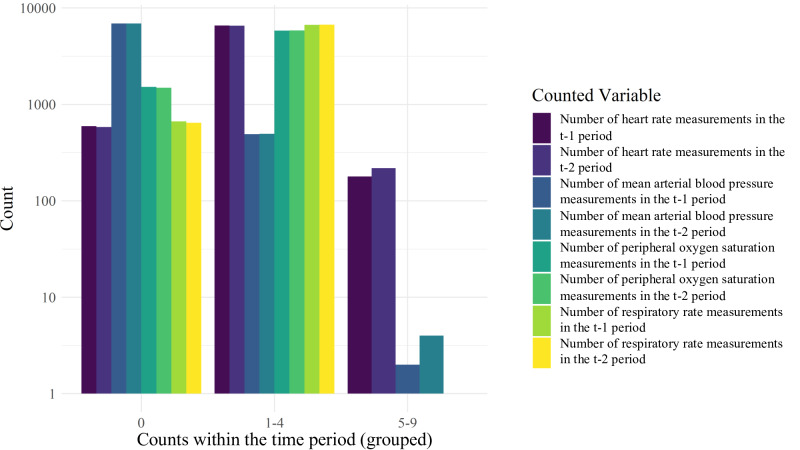
Distribution of data frequency within the time periods per variable. The y-axis is scaled logarithmically; the x-axis shows the groups of frequencies, starting with 0, corresponding to Finnegan scores for which there are no data points for the respective variables within the respective time period. All variables show counts within clusters 0 and 1‐4. Within clusters 5‐9, only counts for heart rate and blood pressure are available in both time periods.

We visualized data availability in a clustered bar plot, reporting the number of data points per variable available for each patient within each period (Figure 4). By computing 2 statistical measures for each of the 4 vital parameter variables in each of the 2 time periods, we obtained a total of 16 graphs (see Multimedia Appendix 1). To visualize data distribution, we generated heat map plots. The color of each tile is set by the number of data points weighted by the number of measurements generating the data point so that data points based on more observations contribute more to the color scale. Hence, 2 data points based on 1 observation each and 1 data point based on 2 observations both result in the same color of the respective tile.

### Mixed Effects Models

We developed several mixed effects models. The models were fitted to descriptively analyze the relationship between the FS and vital parameters as well as NAS type, the time elapsed since the last opioid medication, gestational age, and the percentage of birth weight reached by the most recent body weight measurement. We controlled for interindividual differences by including the patient identifier as the random effect. We generally selected one set of vital parameter variables mixing neither means or individual baseline-controlled means nor the time periods of different vital parameters within a single mixed effects model. The models were not fitted to predict the FS; therefore, neither cross-validation nor bootstrapping were applicable. The regression equation for the full model was as follows, where “mean” could be substituted with “baseline-controlled mean” and “t–1” with “t–2” throughout the equation:

Value of Finnegan-Score~Intercept + Mean heart rate in the t–1 period + Mean peripheral oxygen saturation in the t–1 period + Mean respiratory rate in the t–1 period + Mean of the mean arterial blood pressure in the t–1 period + Mean body temperature within 1 day before documenting the FS + Hours between the last medication as specified and documentation of the FS + Percentage of birth weight + Gestational age + NAS type + (1 / patient identifier)

We evaluated the goodness of fit again for models with a simplified set of variables, excluding variables with small effects (estimates of <0.05) and high degree of missingness (>30% missing values). Goodness of fit was determined using the Akaike information criteria (AIC), Bayesian information criteria (BIC), and *R*^2^_m_ and *R*^2^_c_ and is listed in Multimedia Appendix 1.

For the sensitivity analysis, we excluded the NAS-type variable from the full model. The model’s results and goodness-of-fit data from the sensitivity analysis can be found in Multimedia Appendix 2.

## Results

### Patient Characteristics and Data Composition

Patient demographic data are provided in Table 1, and patient allocation is demonstrated in Figure 3. Out of 205 neonates with NAS, 78 (38%) had been *ICD-10*–coded. Abstinence after intrauterine exposure (P96.1) was coded in 17.4% (n=4) of patients who were classified as having primary NAS and 5.5% (n=10) of patients who were classified as having iNAS. Abstinence after therapeutic exposition (P96.2) was coded in 21.7% (n=5) and 34.6% (n=63) of the respective patient groups. We obtained 7050 FS data points and calculated the baseline-controlled mean for up to 166 (81%) infants (Table 1). Two reasons prevented us from doing so in the other cases: no definable time period (as illustrated in Figure 2) or no data points within the time period to calculate upon.

### Visual Interpretation

As shown in Figure 4, heart rate measurements showed the highest data density and mean blood pressure measurements showed the lowest. Heart rate measurements were the only variable for which at least 5 data points within a time period were commonly available, the only other variable being mean arterial blood pressure in very rare instances.

**Figure 5.**
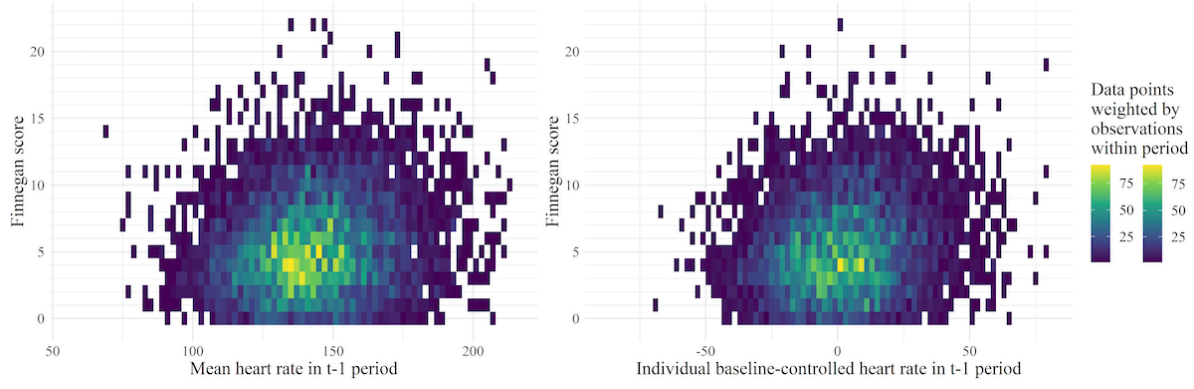
Heat maps of mean heart rate and baseline-controlled mean difference in heart rate during the t–1 time period (60±10 minutes before documenting the Finnegan score [FS]), values of children with iatrogenic neonatal abstinence syndrome. Left: the x-axis shows the mean heart rate in beats per minute (bpm); right: the x-axis shows the baseline-controlled mean difference in heart rate in bpm. Each tile has a width of 2 bpm and a height of 1 FS point; the opacity is generated from the amount of data points within the area of the respective tile weighted by the number of heart rate observations that each of those data points is calculated from.

**Figure 6.**
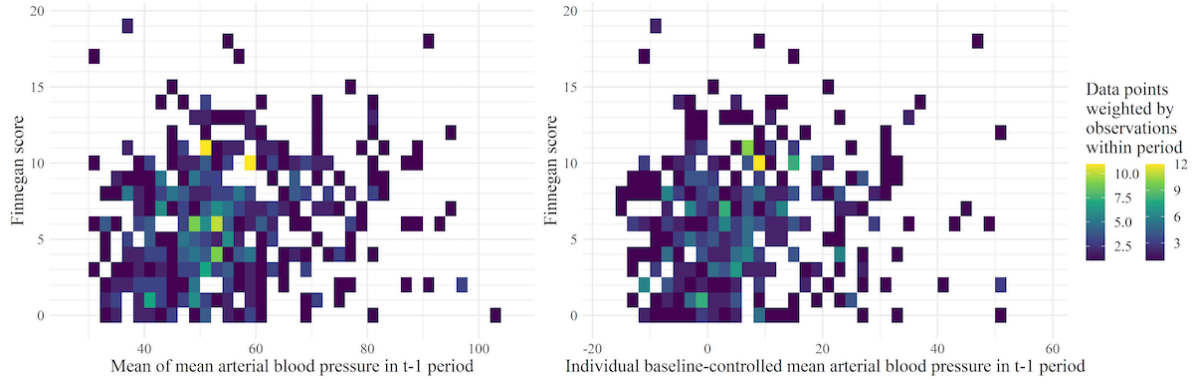
Heat maps of mean and baseline-controlled mean arterial blood pressure during the t–1 period (60±10 minutes before documenting the Finnegan score [FS]) for children with iatrogenic neonatal abstinence syndrome. Left: the x-axis shows the mean arterial blood pressure in mm Hg; right: the x-axis shows the baseline-controlled mean arterial blood pressure in mm Hg. Each tile has a width of 2 mm Hg and a height of 1 FS point; the opacity is generated from the amount of data points within the area of the respective tile weighted by the number of mean arterial blood pressure observations that each of those data points is calculated from.

The plots show a widely distributed pattern for mean and baseline-controlled mean heart rate (Figure 5) and mean arterial blood pressure (Figure 6), each plotted against the FS. The tile color and different ranges shown in the color legends illustrate differences in the density of data available for heart rate and mean arterial blood pressure measurements; this corresponds to the data density described above and is shown in Figure 4. While there was no direct relationship between the FS and the respective parameters visible, the graphs for the baseline-controlled version of each parameter showed a narrower spectrum on the x-axis. In particular, the baseline-controlled blood pressure shows a discernible trend of greater-than-zero values, indicating a rise in blood pressure in neonates in comparison with that before withdrawal assessment. However, this rise in baseline-controlled blood pressure does not clearly increase with an increase in the FS.

### Regression Analysis of Vital Parameters in Fitted Models

Estimates for vital parameters stayed either positive or negative across all fitted models and varied only in the corresponding SEs, CIs, and *P* values. Among all models, the model containing all variables, using the later time period of t–1 and the baseline-controlled mean as the statistical measure, yielded the highest *R*^2^_c_ and *R*^2^_m_ and second-lowest AIC and BIC (Table 2). This model was fitted on 357 observations obtained from 84 infants.

**Table 2.** Results of the mixed model using baseline-controlled means as statistical measures, t–1 as time period, and a complete set of variables (*R*^2^_m_=0.11; *R*^2^_c_=0.43; Akaike information criterion=1999.36; Bayesian information criterion=2045.89; 84 patients; 357 Finnegan scores).

Parameters			Estimate		SE		95% CI		*P* value
(Intercept)			9.31		2.88		3.51 to 14.92		.002
Individual baseline-controlled heart rate in the t–1 period			0.04		0.01		0.02 to 0.07		<.001
Individual baseline-controlled peripheral oxygen saturation in the t–1 period			–0.09		0.05		–0.19 to 0.01		.09
Individual baseline-controlled respiratory rate in the t–1 period			0.00		0.01		–0.02 to 0.03		.84
Individual baseline-controlled mean arterial blood pressure in the t–1 period			0.05		0.02		0.01 to 0.08		.005
Individual baseline-controlled body temperature within 1 day before documenting the Finnegan score			0.33		0.49		–0.63 to 1.28		.51
Hours between last medication as specified and Finnegan score			0.00		0.00		0.00 to 0.00		.46
Percentage of birth weight			0.00		0.00		–0.01 to 0.00		.19
Gestational age			–0.06		0.06		–0.18, to 0.06		.35
Iatrogenic (vs primary) neonatal abstinence syndrome			–0.27		1.64		–3.41 to 2.92		.87

An increasing individual baseline-controlled heart rate (estimate 0.04, 95% CI 0.02 to 0.07) and an increasing arterial blood pressure (estimate 0.05, 95% CI 0.01-0.08) correlated significantly with an increased FS.

Furthermore, a decreasing individual baseline-controlled peripheral oxygen saturation (estimate –0.09, 95% CI −0.19 to 0.01), decreasing gestational age (estimate −0.06, 95% CI −0.18 to 0.06) as well as increasing individual baseline-controlled respiratory rate (estimate 0.00, 95% CI −0.02 to 0.03), increasing hours since the last medication (estimate 0.00, 95% CI 0.00-0.00), increasing baseline-controlled body temperature (estimate 0.33, 95% CI −0.63 to 1.28), increasing percentage of birth weight (estimate 0.00, 95% CI −0.01 to 0.00), and the status of iatrogenic (vs primary) NAS (estimate −0.27, 95% CI −3.41 to 2.92) were associated with an increasing FS. However, while the inclusion of these variables improved the goodness of fit of the model, the estimates showed SEs and 95% CIs too large to be considered significant.

The irregularity and scarcity of blood pressure measurements in neonatal standard care resulted in missing values for the individual baseline-controlled mean arterial blood pressure and thereby reduced the amount of complete data on which the model could be fitted. Due to the large number of missing values (mean blood pressure unavailable for >90% of FS), imputation was not applicable. When blood pressure was not included—thereby reducing the complexity of the model but increasing the number of complete observations—all measures for goodness of fit decreased (worst model with blood pressure [*R*^2^_m_=0.08, AIC=2345]; best model without blood pressure [*R*^2^_m_=0.07, AIC=25,992]). On excluding the NAS type from our sensitivity analysis, these results were confirmed. Including previously excluded patients and using spacer periods of the same length for all patients during baseline calculation resulted in similar results (Multimedia Appendix 2).

The abovementioned estimated effects are small and may seem clinically irrelevant. However, due to the nomenclature of mixed effects models, the estimates refer to 1-unit changes of the respective variable. This implies that a heart rate increase of 10 beats per minute from the individual baseline would coincide with an FS increase of 0.4 and a 10-mm Hg increase (increase of 0.6) in the mean arterial blood pressure assuming that all other values remained constant.

## Discussion

This pilot study shows a measurable association between withdrawal assessment based on FS and heart rate and blood pressure, which underlies heavy scatter and is only revealed when controlling for several other influencing variables in regression analysis. The unfiltered correlation between FS values and vital parameters was weak, and the analysis revealed heavily scattered data. Thus, we fitted mixed effects models that corrected for various variables. These models supported the hypothesis that opioid withdrawal measured by FS is associated with vital parameter readings if big data sets are considered. Multiple analyses revealed robust estimates with a small magnitude for the association of increasing FS with an increased heart rate and arterial blood pressure (Table 2) but not with the respiratory rate. However, even in the model exhibiting the best goodness of fit (*R*^2^_m_=0.11; Table 2), this association was found to be weak, likely because of heavily scattered input data. Notably, our analysis only revealed an association with the FS, which, while being the currently and widely used assessment tool for withdrawal, can only be understood as a resemblance of the latter, not withdrawal itself.

The use of electronic monitoring data and health records may become an attractive source for clinical decision-making (eg, for identifying the risk of sepsis) or multivariable predictive models (eg, for neurodevelopmental impairment) in neonatology [36-39]. While more conservative models perform similarly on the task of predicting sepsis in neonates, advanced machine learning techniques exhibit better performance in case of heterogenous big data pipelines [40].

Conceptually, it is appealing to develop such a big data–based prediction strategy, particularly if it can be validated by using a clinical scoring system. Since opioid withdrawal symptoms considered in the FS rely on the accuracy of reporting, detecting, and describing symptoms, adding monitoring or laboratory data might improve subsequent clinical decisions. However, items in the FS may be too complex per se for fitting common models of analyzing big data from electronic health records. In this regard, the density of electronic monitoring data, such as arterial blood pressure, was surprisingly low in our cohort. Other easily accessible data such as intrauterine growth restriction and maternal tobacco or multisubstance abuse also failed to sufficiently predict NAS severity in previous studies [41]. Success in using continuously monitored electronic data for decision-making in care of neonatal opioid withdrawal, however, may critically depend on the granularity of these data. At our institution, the heart rate may have been recorded with very low data granularity, for example (Figure 4), as these readings were previously summarized to means for storage capacity reasons. This is not unusual as hospital systems regularly do not save the highest available data frequency to reduce the required data storage space, all the while limiting its use for research at later stages [40]. Thus, we applied specific periods (t–1 and t–2; Figures1  2) and identified an individual baseline of vital parameters to compile data with varying temporal resolution. Of note, temporal cross-correlation of vital parameters might have resulted in an improvement in predictive values on the severity of FS, as previously shown in other cohorts of preterm infants with sepsis, necrotizing enterocolitis, or retinopathy [38,42,43].

To date, our data did not offer sufficient temporal resolution to cross-correlate vital parameter data strings. However, based on the most the recent study by Poppe et al [38], we suggest that continuously logged electronic data sampled preferentially at 1 Hz should be obtained. For model development, such high-frequency data may also be obtained during prospective trials. For model validation, real-world data are required at a later stage. High temporal resolution also allows models to reach high levels of goodness of fit and significance with the use of relatively basic sets of variables, as demonstrated for both instability and requirement of treatment after morphine analgesia based on documented episodes of apnea, profound oxygen desaturation, the average heart and respiratory rates, and the postmenstrual age [18]. While our models based on our limited temporal resolution data did not show significant effects of changes in the respiratory rate, the addition of respiratory signals to heart rate characteristics also improved the performance of sepsis prediction models. The resulting model features an especially strong negative predictive value, and we suggest validating this model with larger cohorts [44]. Further research is necessary to not only validate the effects we observed in other cohorts but also analyze associations between high-frequency data and withdrawal, potentially using measures obtainable within these data, such as heart rate characteristics and variations. Most recently, our institution has begun to archive vital parameter data in real time and with high resolution, enabling us to pursue this path.

Our data do not allow considering the different (substance or dose) pharmacologic interventions for neonatal opioid withdrawal and variations in the half-life of such substances when computing the time elapsed since the last administration and the next FS. The complex metabolism of buprenorphine in neonates [19,20] may also affect the analytical mixed models’ performance and be relevant for strategies using artificial intelligence for future clinical decision-making.

Despite these limitations, the discrepancy between the FS and data from electronic monitoring may also reflect an inherent weakness of the clinical score. Since the FS has been reported to be subjective, resulting in low interrater reliability, our study indirectly supports the “Eat, Sleep, Console” approach for neonatal opioid withdrawal as successfully shown in the recent cluster-randomized controlled trial of the ACT NOW Collaborative [45].

## Supplementary material

10.2196/50575 Multimedia Appendix 1 Extented set of figures on vital parameters and tables showing mixed effects model results.

10.2196/50575 Multimedia Appendix 2 Sensitivity analysis.

## Abbreviations

AIC: Akaike information criterion
BIC: Bayesian information criterion
FS: Finnegan score
*ICD-10*: *International Statistical Classification of Diseases, Tenth Revision*
iNAS: iatrogenic neonatal abstinence syndrome
NAS: neonatal abstinence syndrome
PDMS: patient data management system
PoPPI: Procedural Pain in Premature Infants
