# Supplementary material for: The Finnegan Score for Neonatal Opioid Withdrawal Revisited With Routine Electronic Data: Retrospective Study
Source: JMIR Pediatr Parent. 2024 Feb 28;7:e50575. doi: 10.2196/50575 (PMC11004517; doi:10.2196/50575)
Supplement: Multimedia Appendix 1 [file pediatrics-v7-e50575-s001.docx]

Multimedia Appendix to:
Finnegan score revisited by routine electronic data

Till Rech, Kerstin Rubarth, Christoph Bührer, Felix Balzer, Christof Dame

2023-07-05

Table of Contents

[Multimedia Appendix 1: evaluated medication 3](#_Toc139480182)

[Analysis 4](#_Toc139480183)

[Diagrams 4](#_Toc139480184)

[Multimedia Appendix 2: Graphs on heart rate 4](#_Toc139480185)

[Multimedia Appendix 3: Graphs on respiratory rate 6](#_Toc139480186)

[Multimedia Appendix 4: Graphs on peripheral oxygen saturation 8](#_Toc139480187)

[Multimedia Appendix 5: Graphs on mean arterial bloodpressure 10](#_Toc139480188)

[Multimedia Appendix 6: Mixed effects models with full set of variables 12](#_Toc139480189)

[Multimedia Appendix 6.1: Variable set: Mean 12](#_Toc139480190)

[t-1 12](#_Toc139480191)

[t-2 14](#_Toc139480192)

[Multimedia Appendix 6.2: Variable set: Individual baseline-controlled mean 15](#_Toc139480193)

[t-1 15](#_Toc139480194)

[t-2 17](#_Toc139480195)

[Multimedia Appendix 7: Mixed effects model without body temperature 19](#_Toc139480196)

[Multimedia Appendix 7.1: Variable set: Mean 19](#_Toc139480197)

[t-1 19](#_Toc139480198)

[t-2 21](#_Toc139480199)

[Multimedia Appendix 7.2: Variable set: Individual baseline-controlled mean 22](#_Toc139480200)

[t-1 22](#_Toc139480201)

[t-2 24](#_Toc139480202)

[Multimedia Appendix 8: Mixed effects model without bloodpressure, peripheral_oxygen_saturation 26](#_Toc139480203)

[Multimedia Appendix 8.1: Variable set: Mean 26](#_Toc139480204)

[t-1 26](#_Toc139480205)

[t-2 28](#_Toc139480206)

[Multimedia Appendix 8.2: Variable set: Individual baseline-controlled mean 29](#_Toc139480207)

[t-1 29](#_Toc139480208)

[t-2 31](#_Toc139480209)

[Multimedia Appendix 9: Mixed effects models without bloodpressure, peripheral_oxygen_saturation, gest_age, body temperature 33](#_Toc139480210)

[Multimedia Appendix 9.1: Variable set: Mean 33](#_Toc139480211)

[t-1 33](#_Toc139480212)

[t-2 34](#_Toc139480213)

[Multimedia Appendix 9.2: Variable set: Individual baseline-controlled mean 35](#_Toc139480214)

[t-1 35](#_Toc139480215)

[t-2 36](#_Toc139480216)

[Multimedia Appendix 10: Mixed effects models without bloodpressure, peripheral_oxygen_saturation, gestational age, body temperature, percentage of body weight 37](#_Toc139480217)

[Multimedia Appendix 10.1: Variable set: Mean 37](#_Toc139480218)

[t-1 37](#_Toc139480219)

[t-2 38](#_Toc139480220)

[Multimedia Appendix 10.2: Variable set: Individual baseline-controlled mean 39](#_Toc139480221)

[t-1 39](#_Toc139480222)

[t-2 40](#_Toc139480223)

[Multimedia Appendix 11: Model fit comparison including reworked analysis 41](#_Toc139480224)

## Multimedia Appendix 1: evaluated medication

The following medication types were regarded as relevant for classification of primary versus iatrogenic neonatal abstinence syndrome: alfentanil, buprenorphine, clonidine, codeine, fentanyl, hydromorphone, levomethadone, methadone, morphine, nalbuphine, oxycodone, pethidine, phenobarbital, piritramide, remifentanil, sufentanil, tilidine, tramadol.

# Analysis

## Diagrams

### Multimedia Appendix 2: Graphs on heart rate
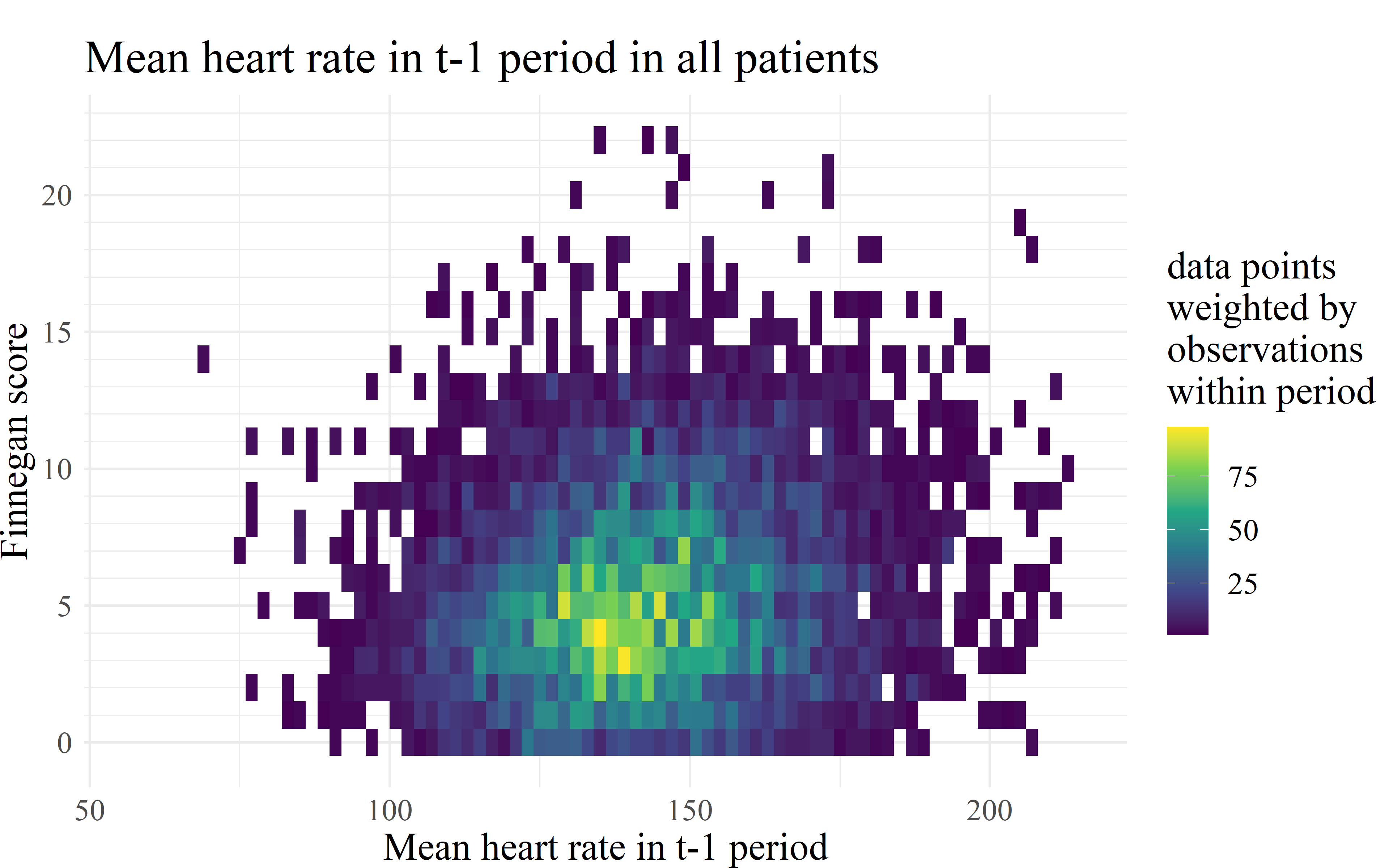

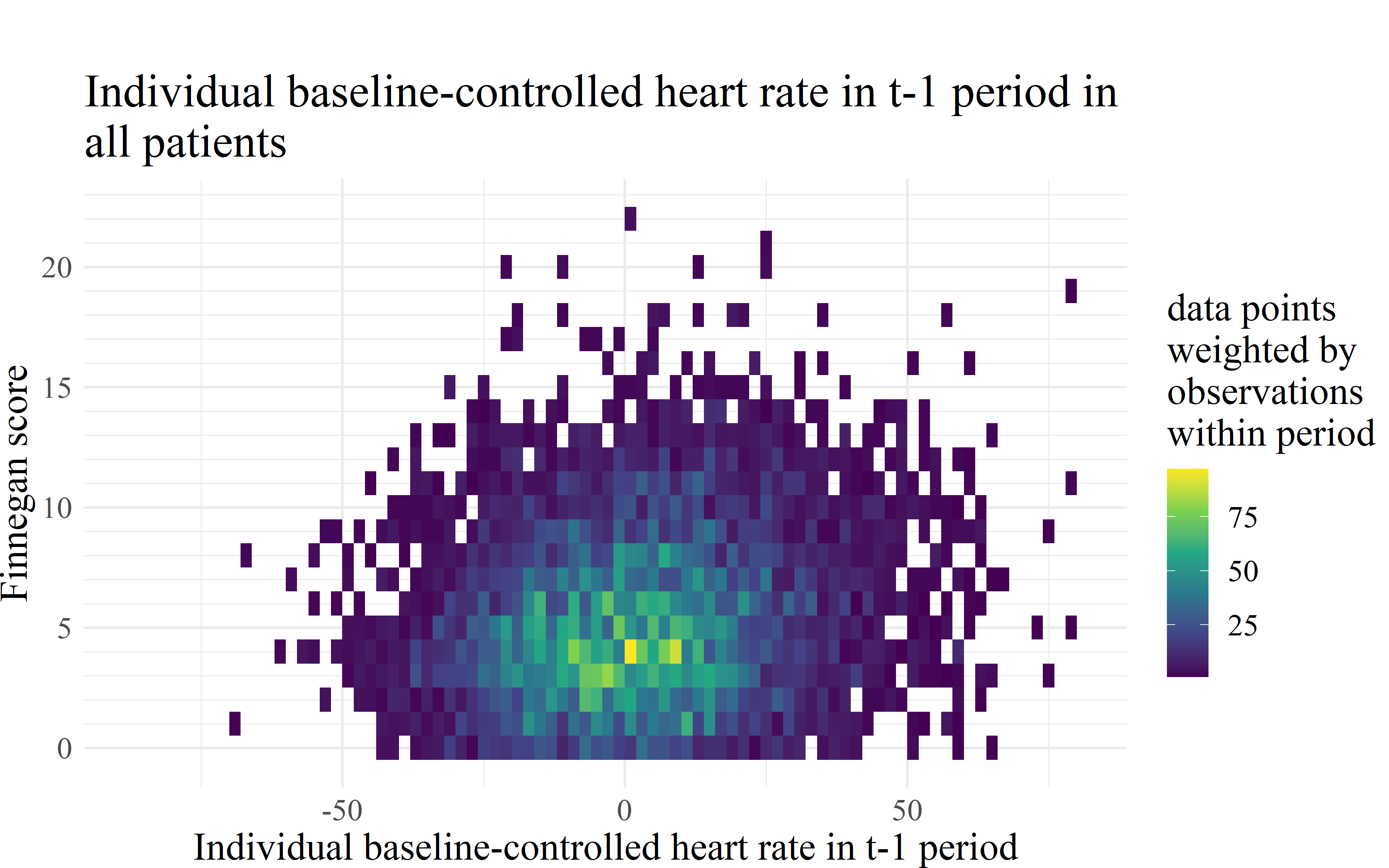


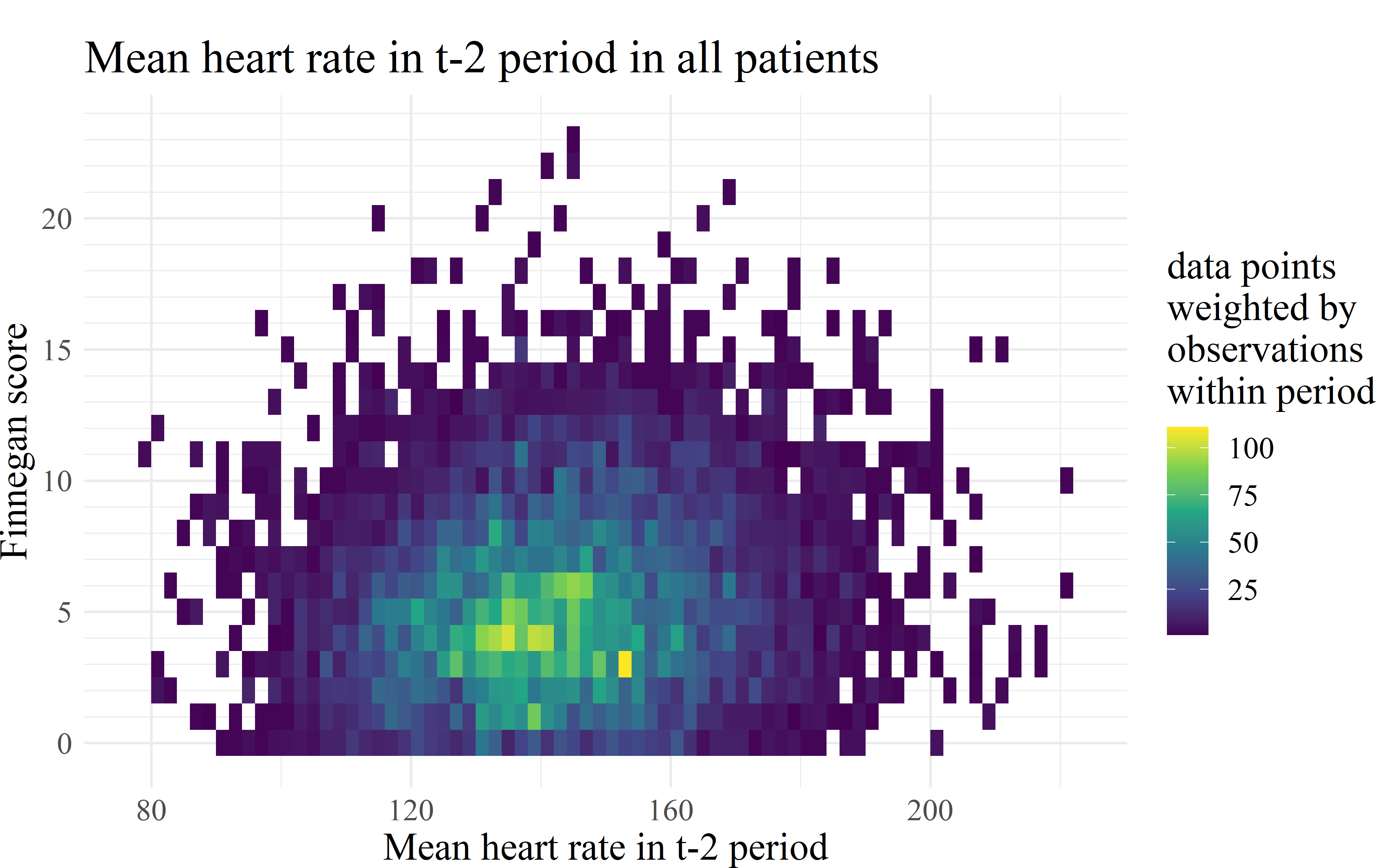

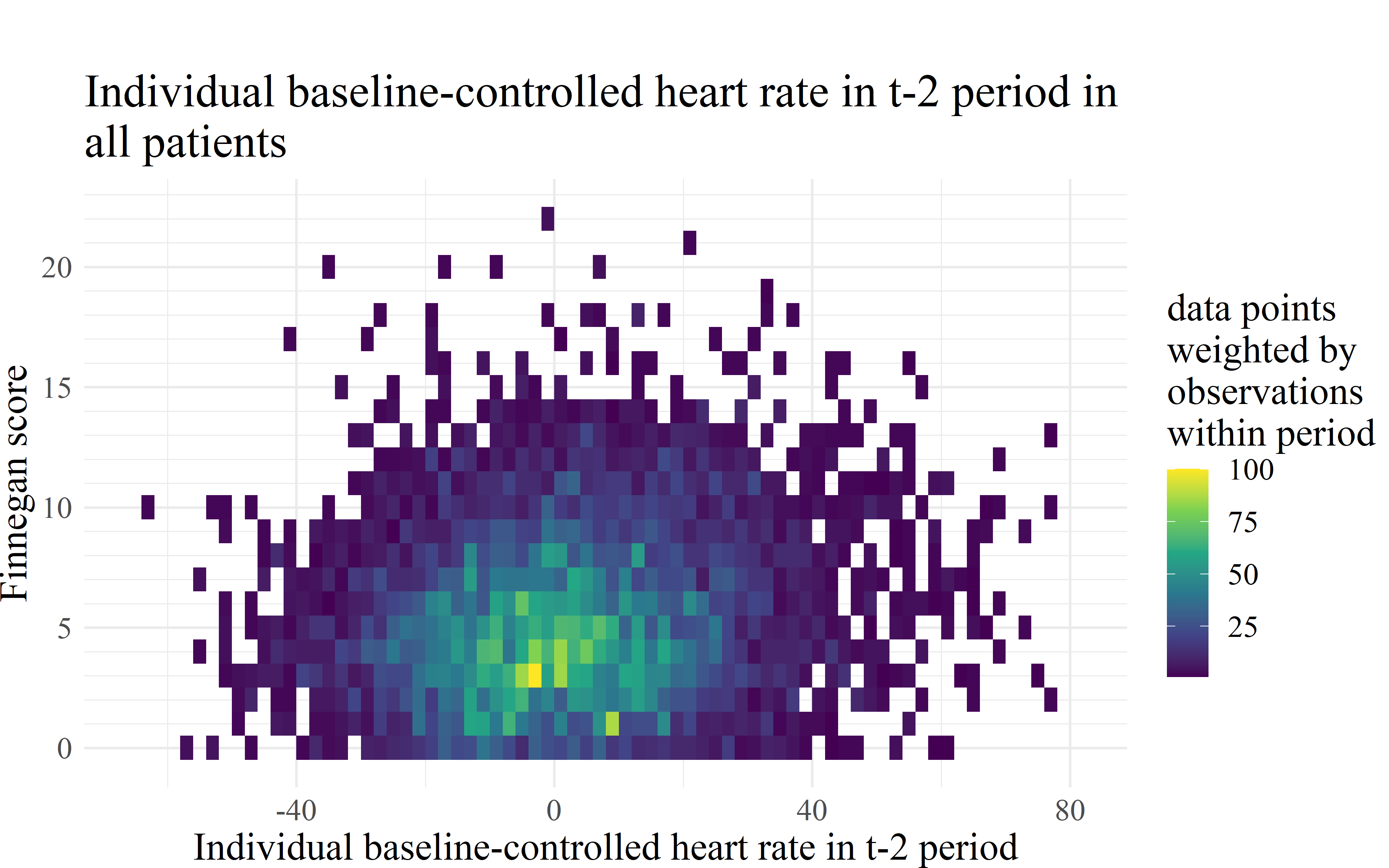


### Multimedia Appendix 3: Graphs on respiratory rate


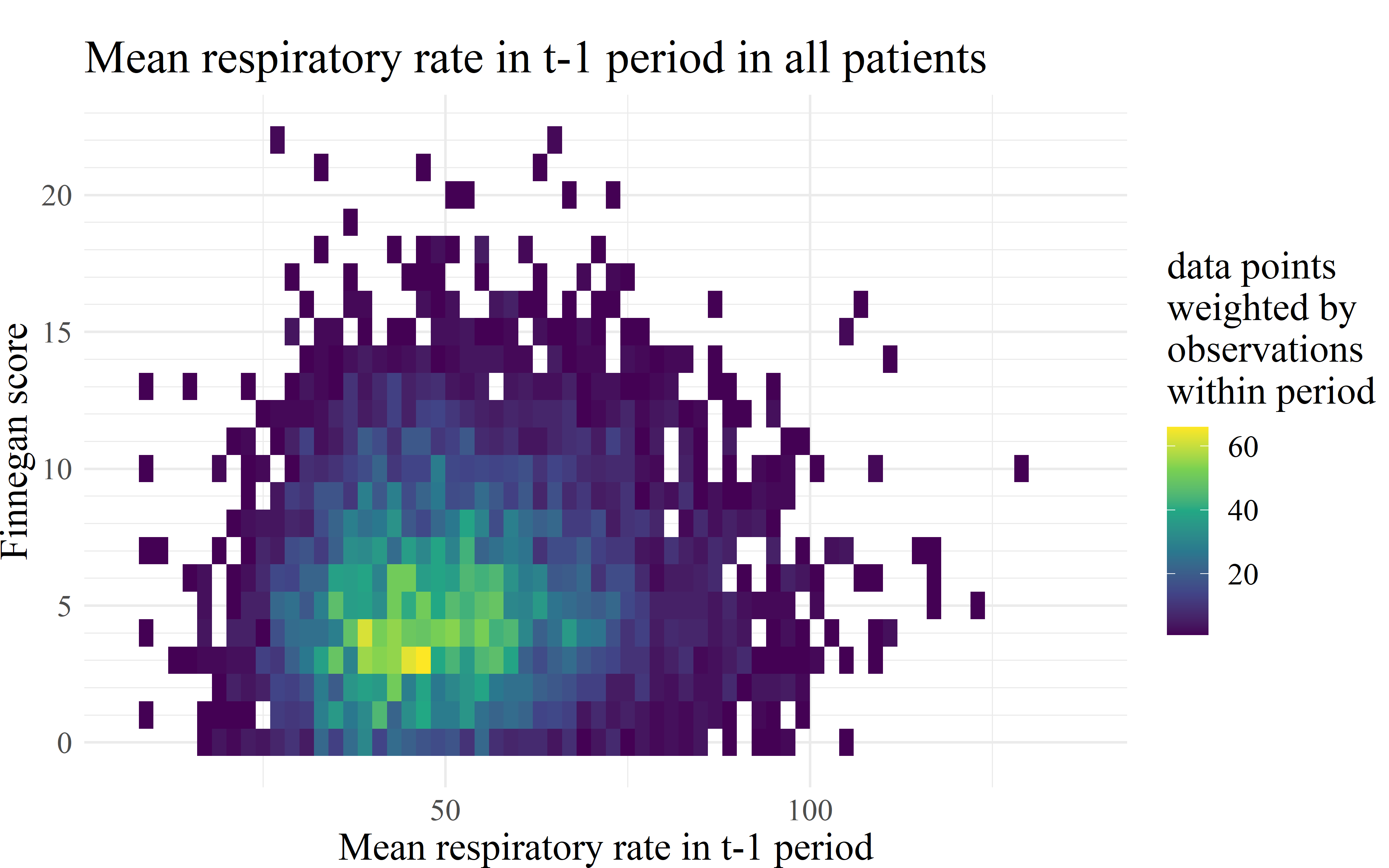

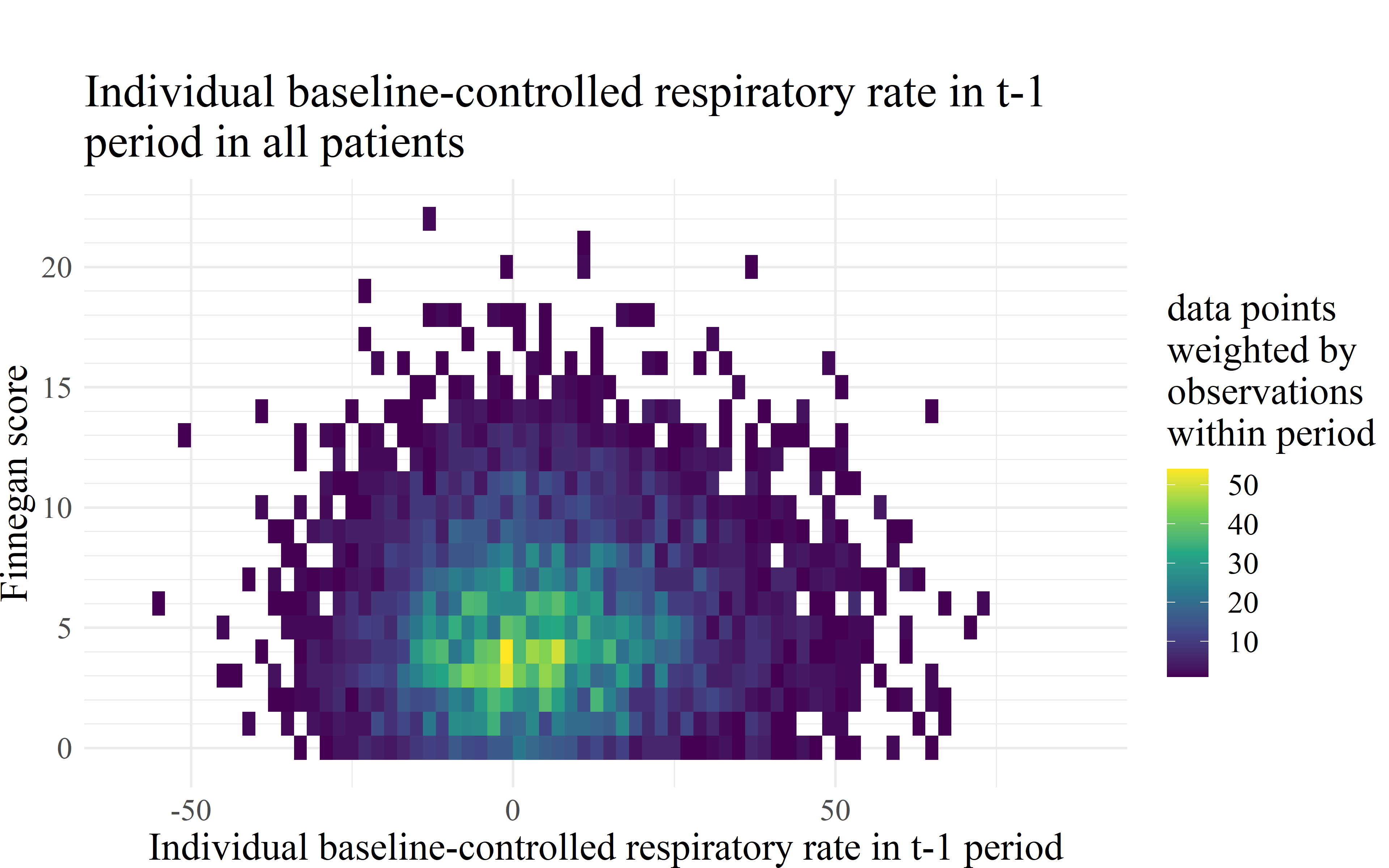

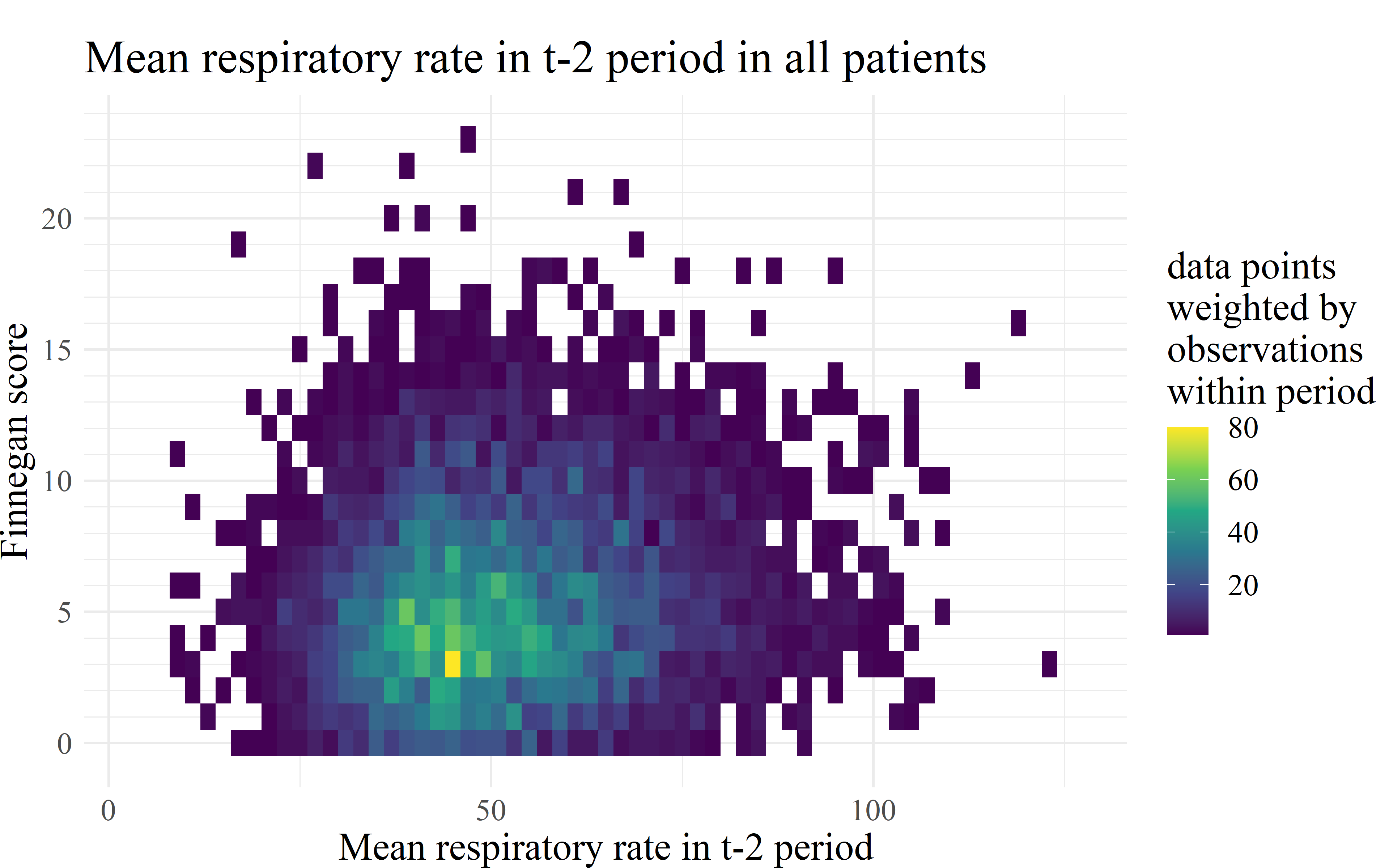

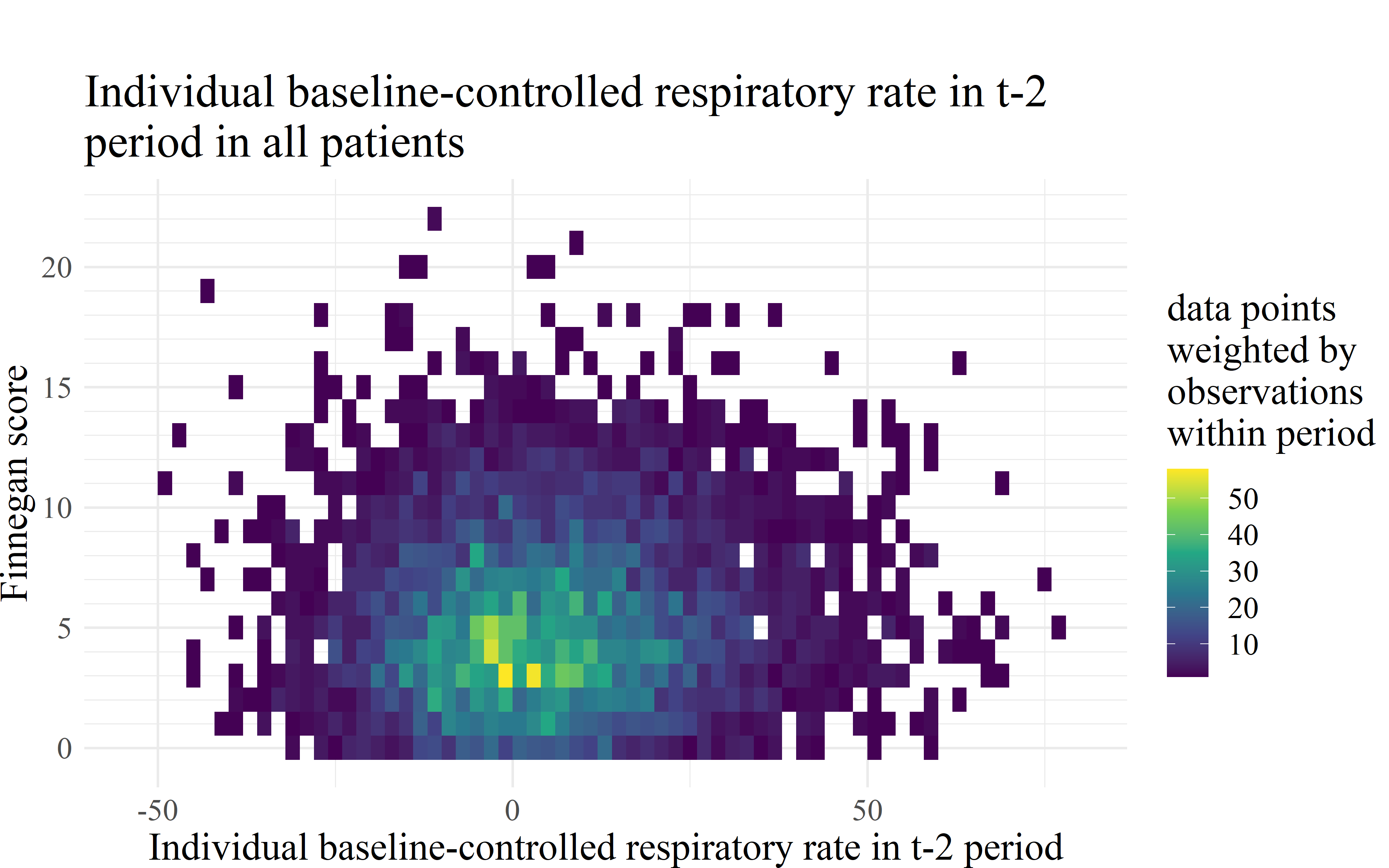


### Multimedia Appendix 4: Graphs on peripheral oxygen saturation


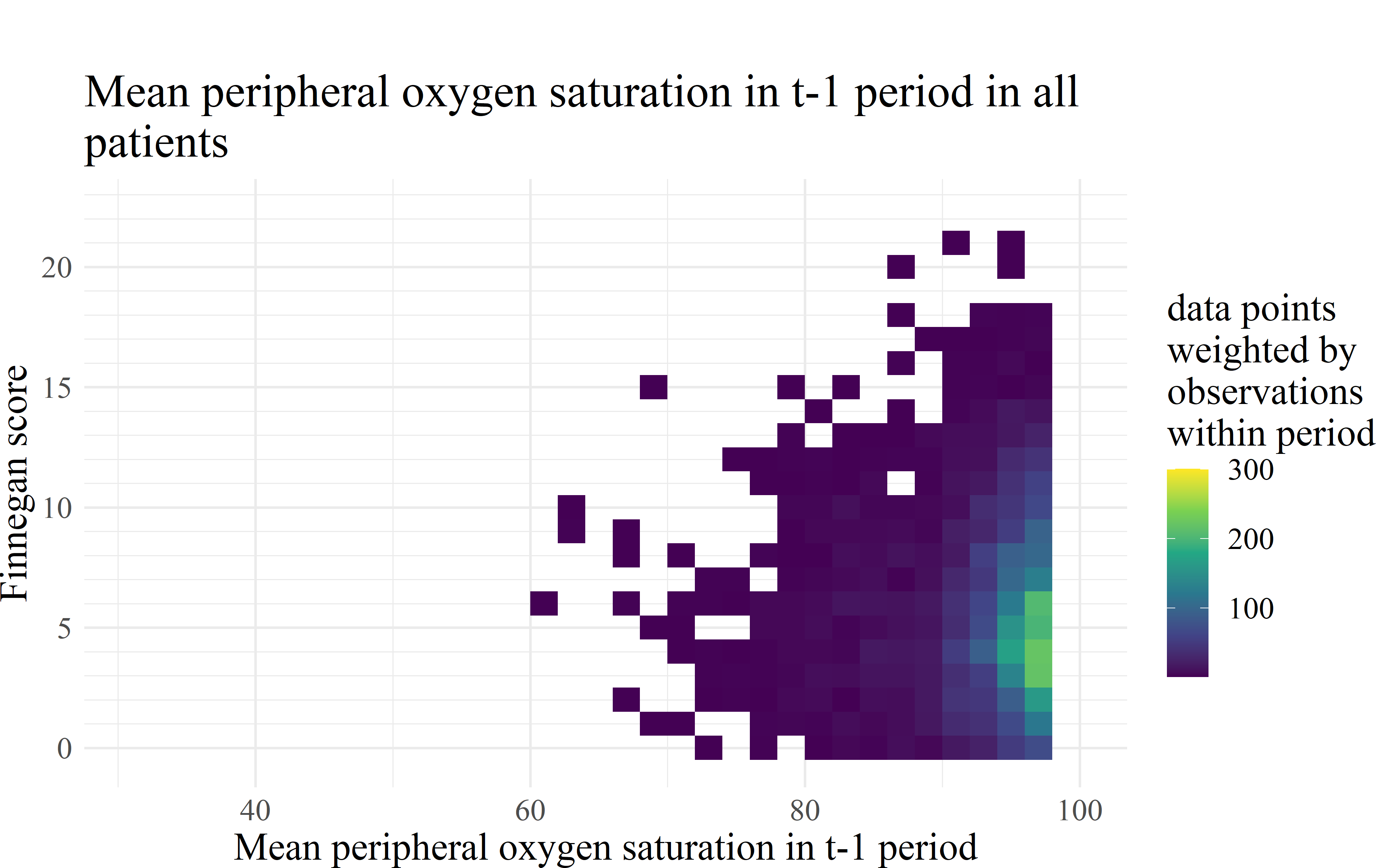

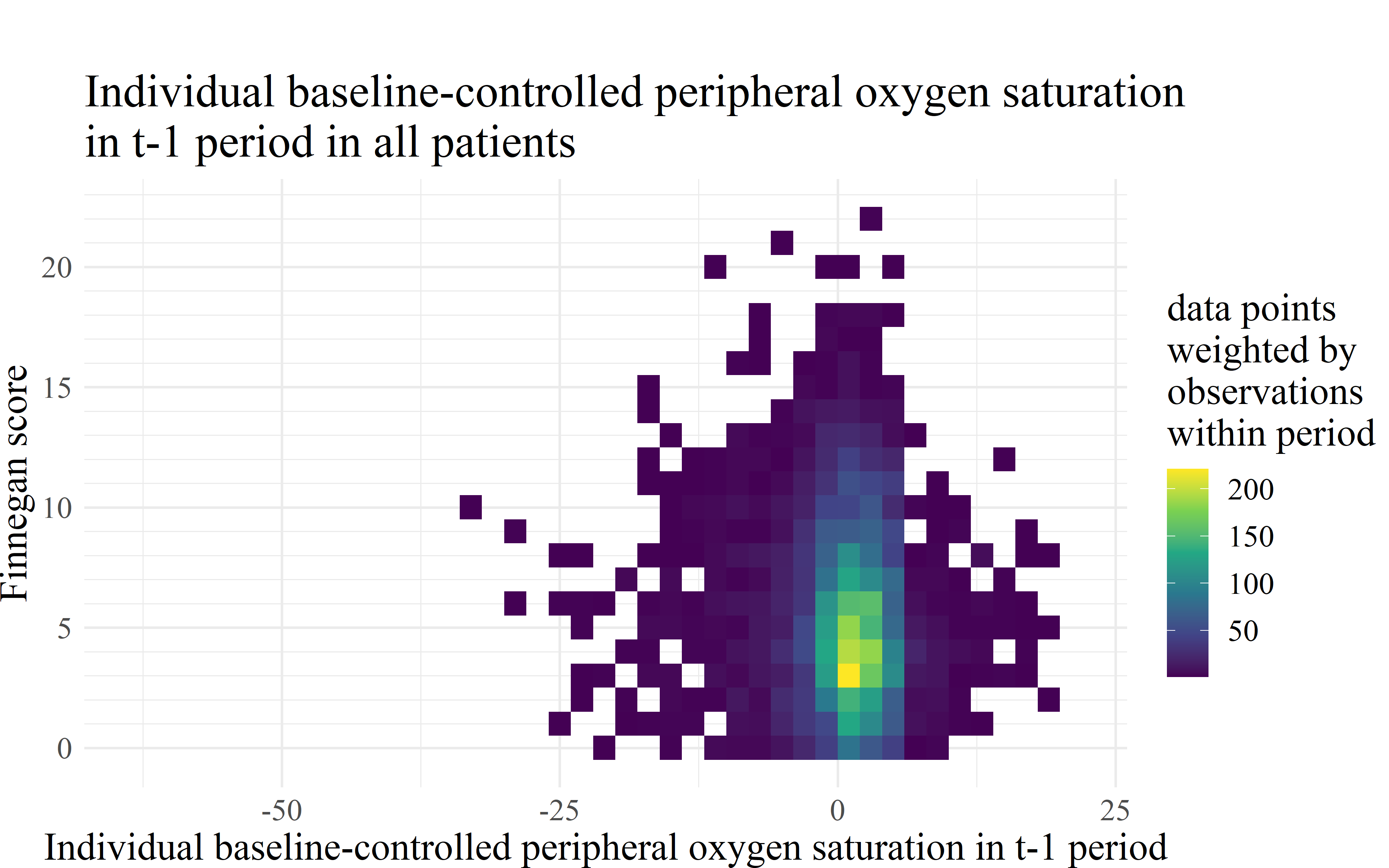

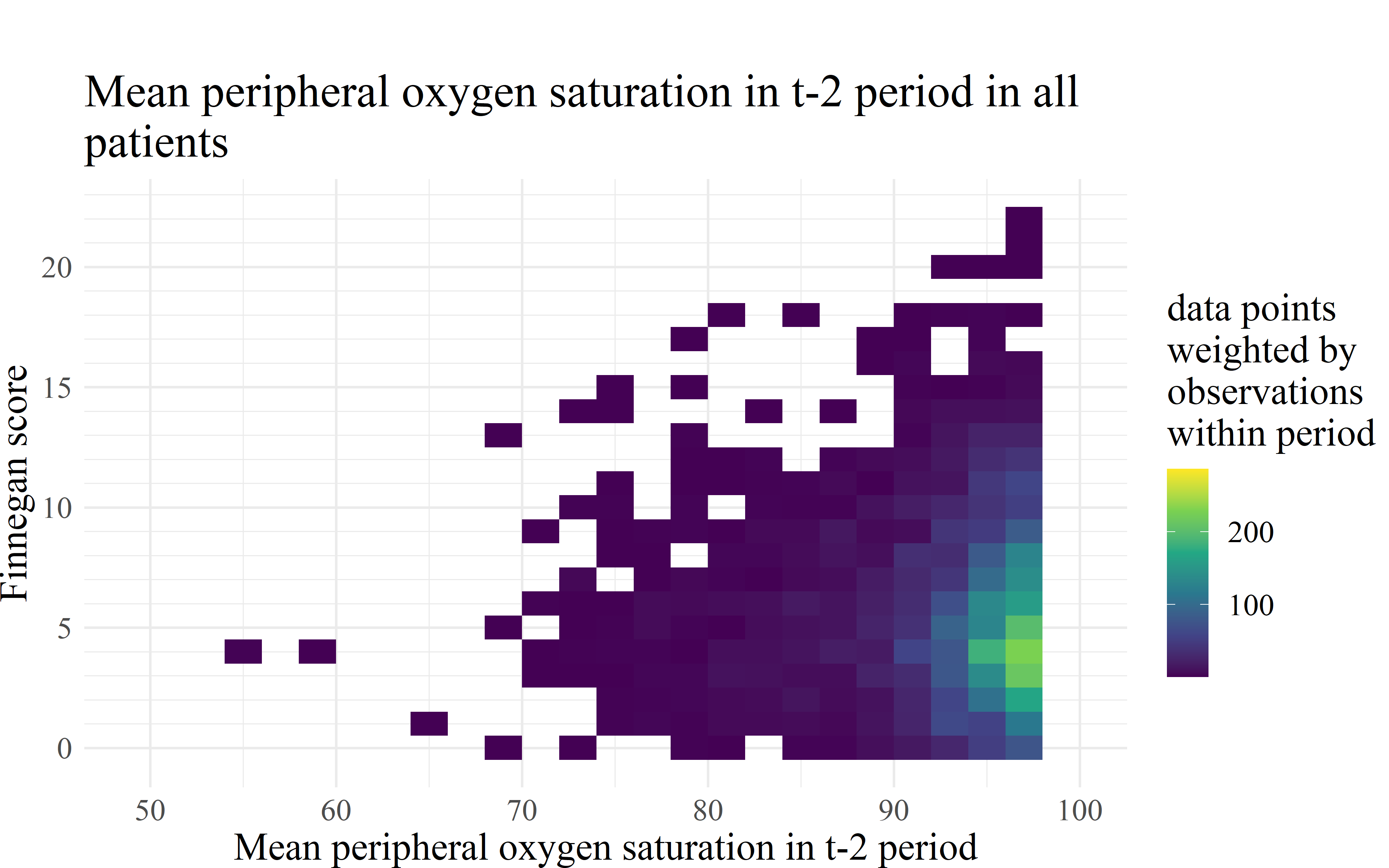

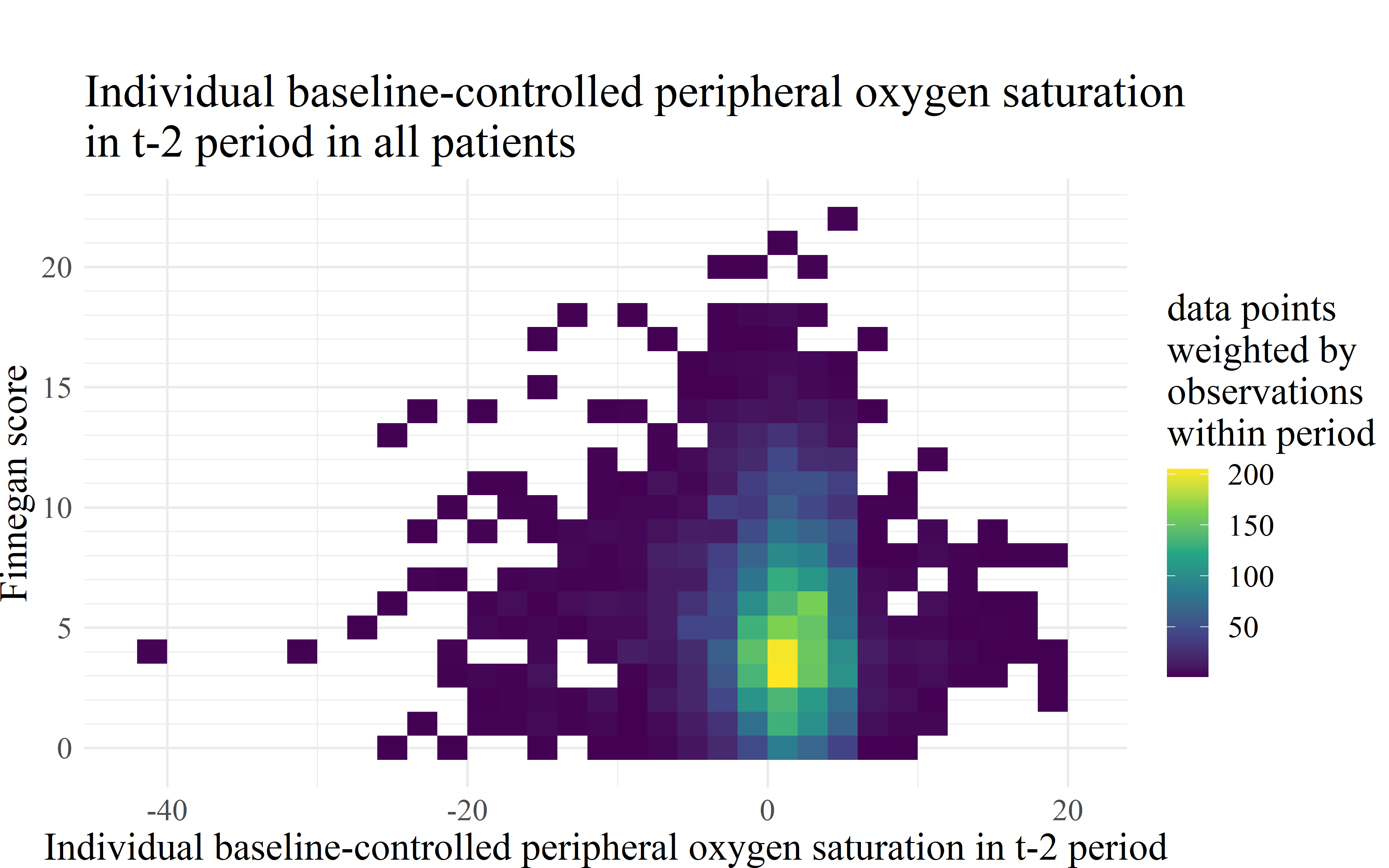


### Multimedia Appendix 5: Graphs on mean arterial bloodpressure


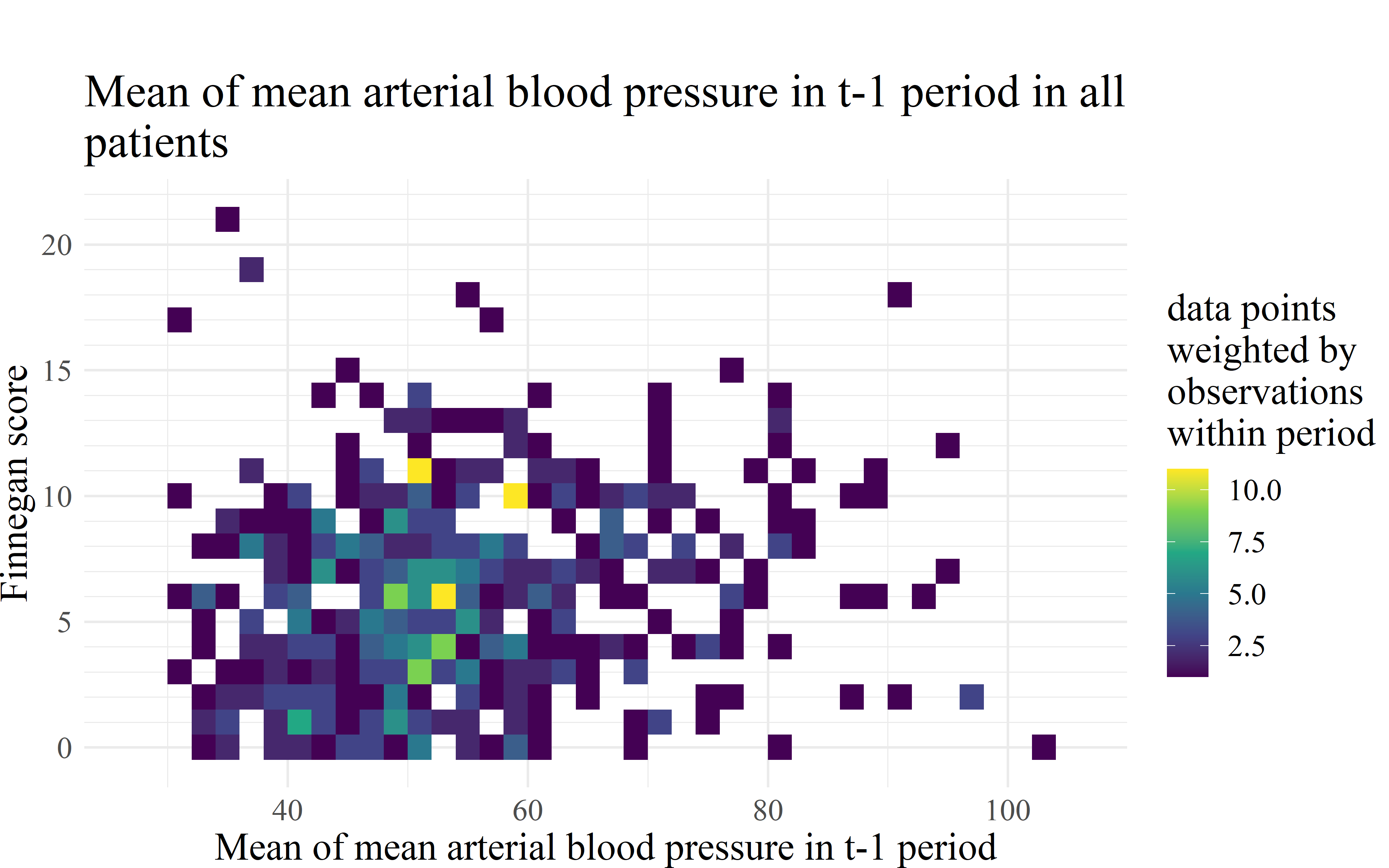

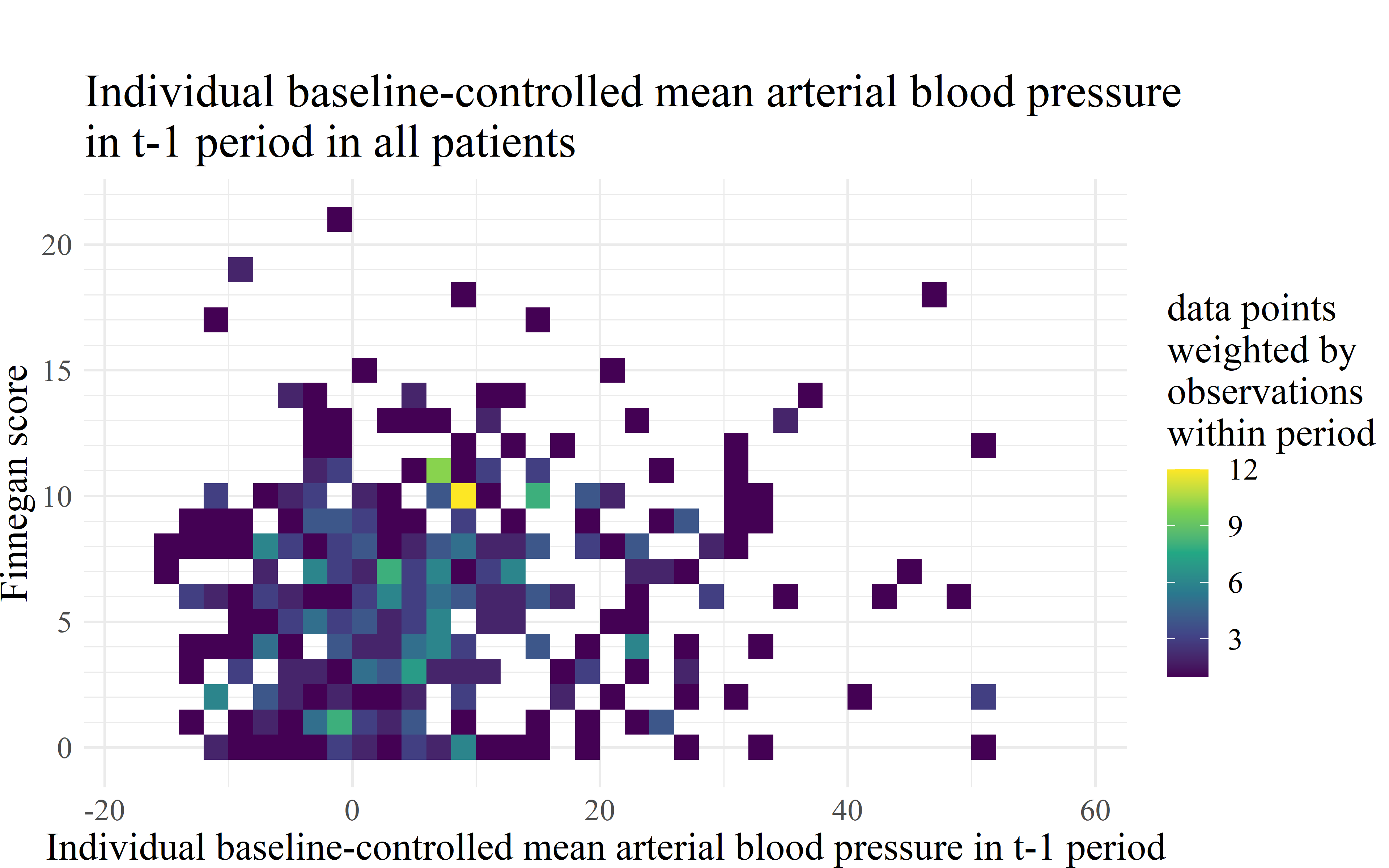

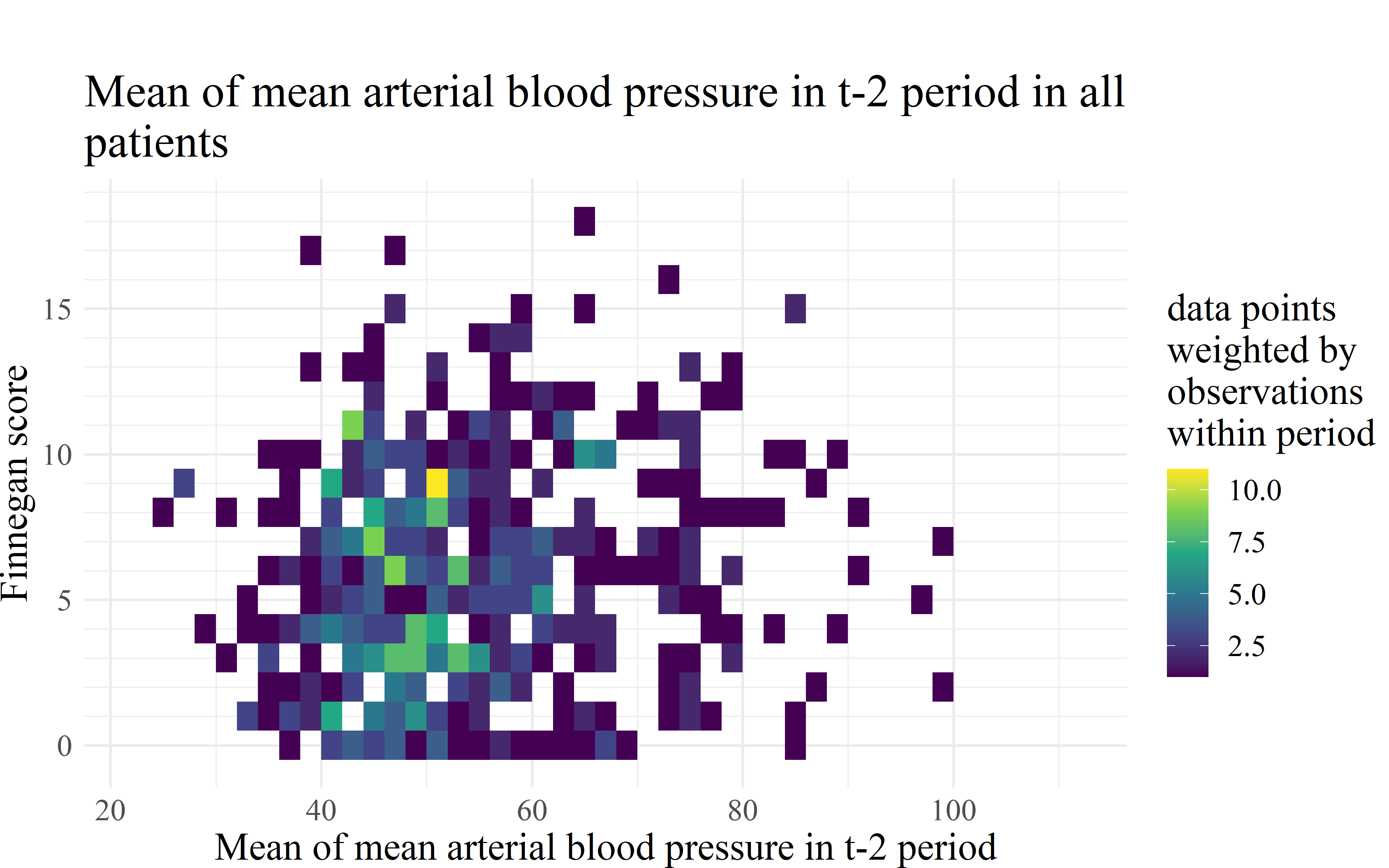

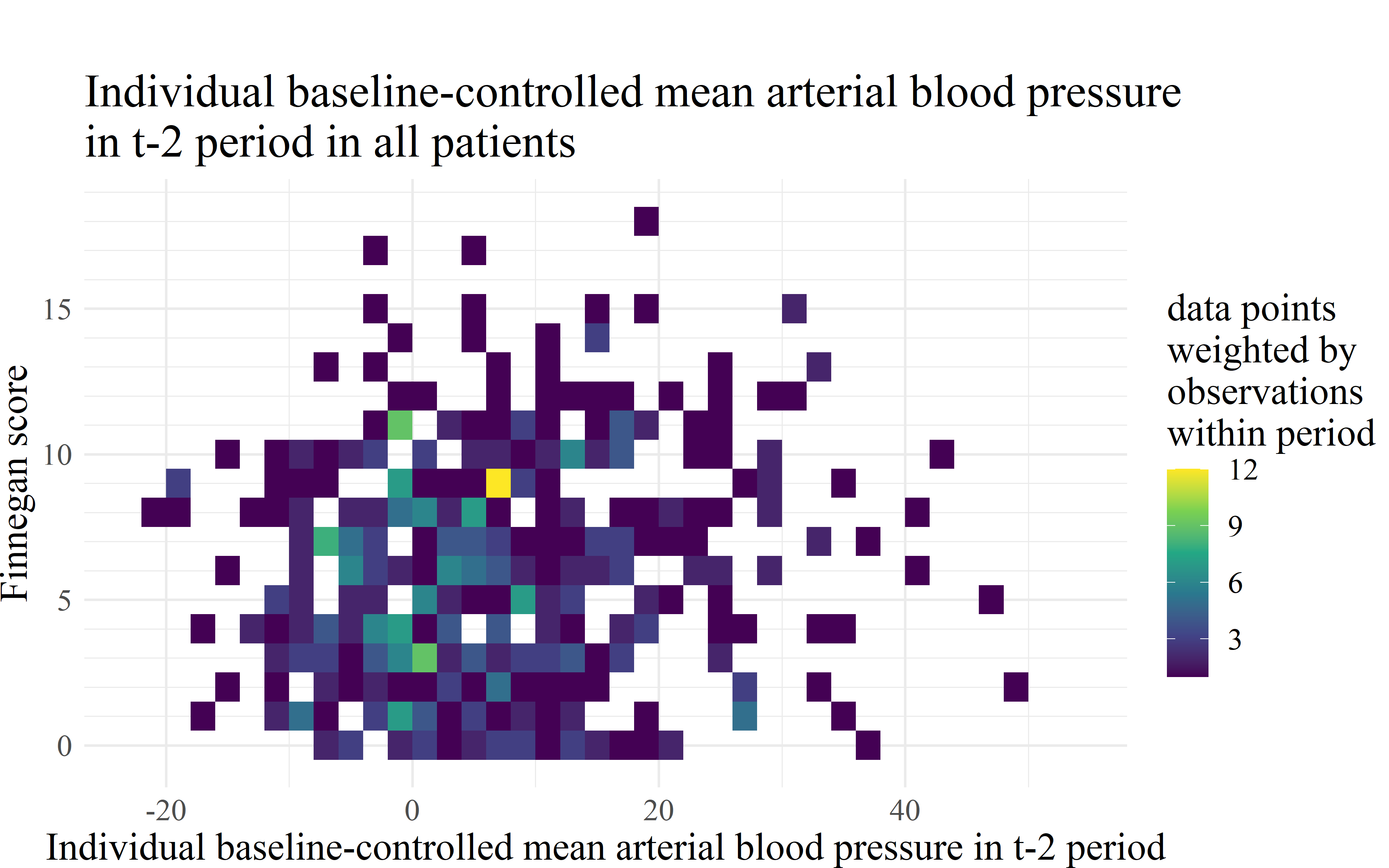


## Multimedia Appendix 6: Mixed effects models with full set of variables

### Multimedia Appendix 6.1: Variable set: Mean

#### t-1

| Table S1: Estimates for Mixed effects model on FS for t-1 using mean; full set of variables | | | | |
| --- | --- | --- | --- | --- |
| formula : Value of Finnegan-Score ~ Mean heart rate in t-1 period + Mean peripheral oxygen saturation in t-1 period + Mean respiratory rate in t-1 period + Mean of mean arterial blood pressure in t-1 period + Mean body temperature within 1 day before Finnegan-Score + Hours between last medication as specified and Finnegan-Score + Percentage of birth weight + Gestational age + NAS-type + ( 1 \| newid ) | | | | |
|  | Estimate | Std. Error | 95%-CI | p-value |
| (Intercept) | -10.61 | 14.80 | [-39.76, 17.61] | 0.474 |
| Mean heart rate in t-1 period | 0.04 | 0.01 | [0.01, 0.06] | 0.001 |
| Mean peripheral oxygen saturation in t-1 period | -0.06 | 0.04 | [-0.14, 0.02] | 0.148 |
| Mean respiratory rate in t-1 period | 0.00 | 0.01 | [-0.02, 0.03] | 0.707 |
| Mean of mean arterial blood pressure in t-1 period | 0.06 | 0.02 | [0.03, 0.09] | 0.000 |
| Mean body temperature within 1 day before Finnegan-Score | 0.46 | 0.40 | [-0.3, 1.22] | 0.248 |
| Hours between last medication as specified and Finnegan-Score | 0.00 | 0.00 | [0.00, 0.00] | 0.307 |
| Percentage of birth weight | -0.01 | 0.00 | [-0.01, 0.00] | 0.053 |
| Gestational age | -0.04 | 0.05 | [-0.15, 0.07] | 0.455 |
| Iatrogenic NAS (vs. primary) | -0.34 | 1.34 | [-2.89, 2.27] | 0.802 |

| Table S2: Goodness to fit for Mixed effects model on FS for t-1 using mean; full set of variables | |
| --- | --- |
|  |  |
| R2m | 0.100 |
| R2c | 0.370 |
| AIC | 2,308.997 |
| BIC | 2,357.423 |
| Patients | 102.000 |
| Finnegan Scores | 418.000 |

#### t-2

| Table S3: Estimates for Mixed effects model on FS for t-2 using mean; full set of variables | | | | |
| --- | --- | --- | --- | --- |
| formula : Value of Finnegan-Score ~ Mean heart rate in t-2 period + Mean peripheral oxygen saturation in t-2 period + Mean respiratory rate in t-2 period + Mean of mean arterial blood pressure in t-2 period + Mean body temperature within 1 day before Finnegan-Score + Hours between last medication as specified and Finnegan-Score + Percentage of birth weight + Gestational age + NAS-type + ( 1 \| newid ) | | | | |
|  | Estimate | Std. Error | 95%-CI | p-value |
| (Intercept) | -44.65 | 16.63 | [-77.01, -13] | 0.008 |
| Mean heart rate in t-2 period | 0.02 | 0.01 | [0.00, 0.04] | 0.022 |
| Mean peripheral oxygen saturation in t-2 period | 0.00 | 0.03 | [-0.06, 0.07] | 0.973 |
| Mean respiratory rate in t-2 period | 0.02 | 0.01 | [-0.01, 0.04] | 0.166 |
| Mean of mean arterial blood pressure in t-2 period | 0.06 | 0.02 | [0.03, 0.09] | 0.000 |
| Mean body temperature within 1 day before Finnegan-Score | 1.19 | 0.45 | [0.34, 2.06] | 0.008 |
| Hours between last medication as specified and Finnegan-Score | 0.00 | 0.00 | [0.00, 0.00] | 0.932 |
| Percentage of birth weight | 0.00 | 0.00 | [-0.01, 0.00] | 0.053 |
| Gestational age | 0.02 | 0.05 | [-0.08, 0.11] | 0.757 |
| Iatrogenic NAS (vs. primary) | -0.61 | 1.17 | [-2.84, 1.66] | 0.601 |

| Table S4: Goodness to fit for Mixed effects model on FS for t-2 using mean; full set of variables | |
| --- | --- |
|  |  |
| R2m | 0.100 |
| R2c | 0.310 |
| AIC | 2,340.328 |
| BIC | 2,388.925 |
| Patients | 101.000 |
| Finnegan Scores | 424.000 |

### Multimedia Appendix 6.2: Variable set: Individual baseline-controlled mean

#### t-1

Included in the publication as Table 2

| Table S5: Estimates for Mixed effects model on FS for t-1 using baseline-controlled mean; full set of variables | | | | |
| --- | --- | --- | --- | --- |
| formula : Value of Finnegan-Score ~ Individual baseline-controlled heart rate in t-1 period + Individual baseline-controlled peripheral oxygen saturation in t-1 period + Individual baseline-controlled respiratory rate in t-1 period + Individual baseline-controlled mean arterial blood pressure in t-1 period + Individual baseline-controlled body temperature within 1 day before Finnegan-Score + Hours between last medication as specified and Finnegan-Score + Percentage of birth weight + Gestational age + NAS-type + ( 1 \| newid ) | | | | |
|  | Estimate | Std. Error | 95%-CI | p-value |
| (Intercept) | 9.31 | 2.88 | [3.51, 14.92] | 0.002 |
| Individual baseline-controlled heart rate in t-1 period | 0.04 | 0.01 | [0.02, 0.07] | 0.000 |
| Individual baseline-controlled peripheral oxygen saturation in t-1 period | -0.09 | 0.05 | [-0.19, 0.01] | 0.086 |
| Individual baseline-controlled respiratory rate in t-1 period | 0.00 | 0.01 | [-0.02, 0.03] | 0.839 |
| Individual baseline-controlled mean arterial blood pressure in t-1 period | 0.05 | 0.02 | [0.01, 0.08] | 0.005 |
| Individual baseline-controlled body temperature within 1 day before Finnegan-Score | 0.33 | 0.49 | [-0.63, 1.28] | 0.505 |
| Hours between last medication as specified and Finnegan-Score | 0.00 | 0.00 | [0.00, 0.00] | 0.460 |
| Percentage of birth weight | 0.00 | 0.00 | [-0.01, 0.00] | 0.189 |
| Gestational age | -0.06 | 0.06 | [-0.18, 0.06] | 0.348 |
| Iatrogenic NAS (vs. primary) | -0.27 | 1.64 | [-3.41, 2.92] | 0.869 |

| Table S6: Goodness to fit for Mixed effects model on FS for t-1 using baseline-controlled mean; full set of variables | |
| --- | --- |
|  |  |
| R2m | 0.110 |
| R2c | 0.430 |
| AIC | 1,999.361 |
| BIC | 2,045.894 |
| Patients | 84.000 |
| Finnegan Scores | 357.000 |

#### t-2

| Table S7: Estimates for Mixed effects model on FS for t-2 using baseline-controlled mean; full set of variables | | | | |
| --- | --- | --- | --- | --- |
| formula : Value of Finnegan-Score ~ Individual baseline-controlled heart rate in t-2 period + Individual baseline-controlled peripheral oxygen saturation in t-2 period + Individual baseline-controlled respiratory rate in t-2 period + Individual baseline-controlled mean arterial blood pressure in t-2 period + Individual baseline-controlled body temperature within 1 day before Finnegan-Score + Hours between last medication as specified and Finnegan-Score + Percentage of birth weight + Gestational age + NAS-type + ( 1 \| newid ) | | | | |
|  | Estimate | Std. Error | 95%-CI | p-value |
| (Intercept) | 6.01 | 2.75 | [0.61, 11.33] | 0.032 |
| Individual baseline-controlled heart rate in t-2 period | 0.03 | 0.01 | [0.01, 0.05] | 0.007 |
| Individual baseline-controlled peripheral oxygen saturation in t-2 period | -0.03 | 0.04 | [-0.1, 0.05] | 0.498 |
| Individual baseline-controlled respiratory rate in t-2 period | 0.01 | 0.01 | [-0.01, 0.03] | 0.395 |
| Individual baseline-controlled mean arterial blood pressure in t-2 period | 0.06 | 0.02 | [0.03, 0.1] | 0.000 |
| Individual baseline-controlled body temperature within 1 day before Finnegan-Score | 0.60 | 0.59 | [-0.55, 1.73] | 0.314 |
| Hours between last medication as specified and Finnegan-Score | 0.00 | 0.00 | [0.00, 0.00] | 0.800 |
| Percentage of birth weight | 0.00 | 0.00 | [-0.01, 0.00] | 0.078 |
| Gestational age | 0.00 | 0.06 | [-0.11, 0.12] | 0.954 |
| Iatrogenic NAS (vs. primary) | 0.81 | 1.62 | [-2.32, 3.95] | 0.620 |

| Table S8: Goodness to fit for Mixed effects model on FS for t-2 using baseline-controlled mean; full set of variables | |
| --- | --- |
|  |  |
| R2m | 0.090 |
| R2c | 0.380 |
| AIC | 2,010.923 |
| BIC | 2,057.589 |
| Patients | 83.000 |
| Finnegan Scores | 361.000 |

## Multimedia Appendix 7: Mixed effects model without body temperature

### Multimedia Appendix 7.1: Variable set: Mean

#### t-1

| Table S9: Estimates for Mixed effects model on FS for t-1 using mean; body temperature removed from set of variables | | | | |
| --- | --- | --- | --- | --- |
| formula : Value of Finnegan-Score ~ Mean heart rate in t-1 period + Mean peripheral oxygen saturation in t-1 period + Mean respiratory rate in t-1 period + Mean of mean arterial blood pressure in t-1 period + Hours between last medication as specified and Finnegan-Score + Percentage of birth weight + Gestational age + NAS- type + ( 1 \| newid ) | | | | |
|  | Estimate | Std. Error | 95%-CI | p-value |
| (Intercept) | 5.60 | 4.90 | [-4.35, 15.05] | 0.254 |
| Mean heart rate in t-1 period | 0.04 | 0.01 | [0.02, 0.06] | 0.000 |
| Mean peripheral oxygen saturation in t-1 period | -0.06 | 0.04 | [-0.14, 0.02] | 0.139 |
| Mean respiratory rate in t-1 period | 0.00 | 0.01 | [-0.02, 0.03] | 0.685 |
| Mean of mean arterial blood pressure in t-1 period | 0.06 | 0.02 | [0.03, 0.09] | 0.000 |
| Hours between last medication as specified and Finnegan-Score | 0.00 | 0.00 | [0.00, 0.00] | 0.330 |
| Percentage of birth weight | -0.01 | 0.00 | [-0.01, 0.00] | 0.053 |
| Gestational age | -0.04 | 0.05 | [-0.15, 0.07] | 0.460 |
| Iatrogenic NAS (vs. primary) | 0.27 | 1.23 | [-2.09, 2.68] | 0.824 |

| Table S10: Goodness to fit for Mixed effects model on FS for t-1 using mean; body temperature removed from set of variables | |
| --- | --- |
|  |  |
| R2m | 0.10 |
| R2c | 0.37 |
| AIC | 2,308.32 |
| BIC | 2,352.71 |
| Patients | 102.00 |
| Finnegan Scores | 418.00 |

#### t-2

| Table S11: Estimates for Mixed effects model on FS for t-2 using mean; body temperature removed from set of variables | | | | |
| --- | --- | --- | --- | --- |
| formula : Value of Finnegan-Score ~ Mean heart rate in t-2 period + Mean peripheral oxygen saturation in t-2 period + Mean respiratory rate in t-2 period + Mean of mean arterial blood pressure in t-2 period + Hours between last medication as specified and Finnegan-Score + Percentage of birth weight + Gestational age + NAS- type + ( 1 \| newid ) | | | | |
|  | Estimate | Std. Error | 95%-CI | p-value |
| (Intercept) | -1.58 | 4.22 | [-9.94, 6.51] | 0.709 |
| Mean heart rate in t-2 period | 0.03 | 0.01 | [0.01, 0.05] | 0.006 |
| Mean peripheral oxygen saturation in t-2 period | 0.00 | 0.03 | [-0.07, 0.06] | 0.933 |
| Mean respiratory rate in t-2 period | 0.01 | 0.01 | [-0.01, 0.04] | 0.203 |
| Mean of mean arterial blood pressure in t-2 period | 0.06 | 0.02 | [0.03, 0.09] | 0.000 |
| Hours between last medication as specified and Finnegan-Score | 0.00 | 0.00 | [0.00, 0.00] | 0.906 |
| Percentage of birth weight | 0.00 | 0.00 | [-0.01, 0.00] | 0.072 |
| Gestational age | 0.02 | 0.05 | [-0.08, 0.12] | 0.737 |
| Iatrogenic NAS (vs. primary) | 0.15 | 1.16 | [-2.09, 2.42] | 0.896 |

| Table S12: Goodness to fit for Mixed effects model on FS for t-2 using mean; body temperature removed from set of variables | |
| --- | --- |
|  |  |
| R2m | 0.080 |
| R2c | 0.320 |
| AIC | 2,345.508 |
| BIC | 2,390.055 |
| Patients | 101.000 |
| Finnegan Scores | 424.000 |

### Multimedia Appendix 7.2: Variable set: Individual baseline-controlled mean

#### t-1

| Table S13: Estimates for Mixed effects model on FS for t-1 using baseline-controlled mean; body temperature removed from set of variables | | | | |
| --- | --- | --- | --- | --- |
| formula : Value of Finnegan-Score ~ Individual baseline-controlled heart rate in t-1 period + Individual baseline-controlled peripheral oxygen saturation in t-1 period + Individual baseline-controlled respiratory rate in t-1 period + Individual baseline-controlled mean arterial blood pressure in t-1 period + Hours between last medication as specified and Finnegan-Score + Percentage of birth weight + Gestational age + NAS-type + ( 1 \| newid ) | | | | |
|  | Estimate | Std. Error | 95%-CI | p-value |
| (Intercept) | 9.07 | 2.84 | [3.35, 14.63] | 0.002 |
| Individual baseline-controlled heart rate in t-1 period | 0.05 | 0.01 | [0.02, 0.07] | 0.000 |
| Individual baseline-controlled peripheral oxygen saturation in t-1 period | -0.08 | 0.05 | [-0.18, 0.02] | 0.105 |
| Individual baseline-controlled respiratory rate in t-1 period | 0.00 | 0.01 | [-0.02, 0.03] | 0.854 |
| Individual baseline-controlled mean arterial blood pressure in t-1 period | 0.05 | 0.02 | [0.01, 0.08] | 0.005 |
| Hours between last medication as specified and Finnegan-Score | 0.00 | 0.00 | [0.00, 0.00] | 0.476 |
| Percentage of birth weight | 0.00 | 0.00 | [-0.01, 0.00] | 0.182 |
| Gestational age | -0.05 | 0.06 | [-0.17, 0.07] | 0.368 |
| Iatrogenic NAS (vs. primary) | -0.14 | 1.62 | [-3.25, 3.01] | 0.930 |

| Table S14: Goodness to fit for Mixed effects model on FS for t-1 using baseline-controlled mean; body temperature removed from set of variables | |
| --- | --- |
|  |  |
| R2m | 0.110 |
| R2c | 0.420 |
| AIC | 2,008.566 |
| BIC | 2,051.283 |
| Patients | 85.000 |
| Finnegan Scores | 359.000 |

#### t-2

| Table S15: Estimates for Mixed effects model on FS for t-1 using baseline-controlled mean; body temperature removed from set of variables | | | | |
| --- | --- | --- | --- | --- |
| formula : Value of Finnegan-Score ~ Individual baseline-controlled heart rate in t-2 period + Individual baseline-controlled peripheral oxygen saturation in t-2 period + Individual baseline-controlled respiratory rate in t-2 period + Individual baseline-controlled mean arterial blood pressure in t-2 period + Hours between last medication as specified and Finnegan-Score + Percentage of birth weight + Gestational age + NAS-type + ( 1 \| newid ) | | | | |
|  | Estimate | Std. Error | 95%-CI | p-value |
| (Intercept) | 6.11 | 2.73 | [0.75, 11.4] | 0.028 |
| Individual baseline-controlled heart rate in t-2 period | 0.03 | 0.01 | [0.01, 0.06] | 0.004 |
| Individual baseline-controlled peripheral oxygen saturation in t-2 period | -0.02 | 0.04 | [-0.1, 0.05] | 0.528 |
| Individual baseline-controlled respiratory rate in t-2 period | 0.01 | 0.01 | [-0.01, 0.03] | 0.368 |
| Individual baseline-controlled mean arterial blood pressure in t-2 period | 0.06 | 0.02 | [0.03, 0.1] | 0.000 |
| Hours between last medication as specified and Finnegan-Score | 0.00 | 0.00 | [0.00, 0.00] | 0.787 |
| Percentage of birth weight | 0.00 | 0.00 | [-0.01, 0.00] | 0.075 |
| Gestational age | 0.00 | 0.06 | [-0.11, 0.11] | 0.944 |
| Iatrogenic NAS (vs. primary) | 0.71 | 1.61 | [-2.4, 3.83] | 0.658 |

| Table S16: Goodness to fit for Mixed effects model on FS for t-1 using baseline-controlled mean; body temperature removed from set of variables | |
| --- | --- |
|  |  |
| R2m | 0.090 |
| R2c | 0.380 |
| AIC | 2,024.581 |
| BIC | 2,067.450 |
| Patients | 84.000 |
| Finnegan Scores | 364.000 |

## Multimedia Appendix 8: Mixed effects model without bloodpressure, peripheral_oxygen_saturation

### Multimedia Appendix 8.1: Variable set: Mean

#### t-1

| Table S17: Estimates for Mixed effects model on FS for t-1 using mean; blood pressure and peripheral oxygen saturation removed from set of variables | | | | |
| --- | --- | --- | --- | --- |
| formula : Value of Finnegan-Score ~ Mean heart rate in t-1 period + Mean respiratory rate in t-1 period + Mean body temperature within 1 day before Finnegan-Score + Hours between last medication as specified and Finnegan- Score + Percentage of birth weight + Gestational age + NAS-type + ( 1 \| newid ) | | | | |
|  | Estimate | Std. Error | 95%-CI | p-value |
| (Intercept) | -3.82 | 3.64 | [-11.04, 3.29] | 0.294 |
| Mean heart rate in t-1 period | 0.03 | 0.00 | [0.03, 0.04] | 0.000 |
| Mean respiratory rate in t-1 period | 0.01 | 0.00 | [0.00, 0.02] | 0.000 |
| Mean body temperature within 1 day before Finnegan-Score | 0.22 | 0.10 | [0.02, 0.41] | 0.029 |
| Hours between last medication as specified and Finnegan-Score | 0.00 | 0.00 | [0.00, 0.00] | 0.003 |
| Percentage of birth weight | 0.00 | 0.00 | [0.00, 0.00] | 0.015 |
| Gestational age | -0.08 | 0.02 | [-0.11, -0.04] | 0.000 |
| Iatrogenic NAS (vs. primary) | 0.32 | 0.47 | [-0.61, 1.24] | 0.504 |

| Table S18: Goodness to fit for Mixed effects model on FS for t-1 using mean; blood pressure and peripheral oxygen saturation removed from set of variables | |
| --- | --- |
|  |  |
| R2m | 0.06 |
| R2c | 0.25 |
| AIC | 31,753.24 |
| BIC | 31,820.37 |
| Patients | 199.00 |
| Finnegan Scores | 6,083.00 |

#### t-2

| Table S19: Estimates for Mixed effects model on FS for t-2 using mean; blood pressure and peripheral oxygen saturation removed from set of variables | | | | |
| --- | --- | --- | --- | --- |
| formula : Value of Finnegan-Score ~ Mean heart rate in t-2 period + Mean respiratory rate in t-2 period + Mean body temperature within 1 day before Finnegan-Score + Hours between last medication as specified and Finnegan- Score + Percentage of birth weight + Gestational age + NAS-type + ( 1 \| newid ) | | | | |
|  | Estimate | Std. Error | 95%-CI | p-value |
| (Intercept) | -4.57 | 3.65 | [-11.78, 2.56] | 0.211 |
| Mean heart rate in t-2 period | 0.03 | 0.00 | [0.02, 0.03] | 0.000 |
| Mean respiratory rate in t-2 period | 0.01 | 0.00 | [0.01, 0.02] | 0.000 |
| Mean body temperature within 1 day before Finnegan-Score | 0.25 | 0.10 | [0.06, 0.45] | 0.011 |
| Hours between last medication as specified and Finnegan-Score | 0.00 | 0.00 | [0.00, 0.00] | 0.006 |
| Percentage of birth weight | 0.00 | 0.00 | [0.00, 0.00] | 0.036 |
| Gestational age | -0.08 | 0.02 | [-0.12, -0.05] | 0.000 |
| Iatrogenic NAS (vs. primary) | 0.34 | 0.47 | [-0.58, 1.27] | 0.471 |

| Table S20: Goodness to fit for Mixed effects model on FS for t-2 using mean; blood pressure and peripheral oxygen saturation removed from set of variables | |
| --- | --- |
|  |  |
| R2m | 0.05 |
| R2c | 0.25 |
| AIC | 31,878.80 |
| BIC | 31,945.97 |
| Patients | 199.00 |
| Finnegan Scores | 6,105.00 |

### Multimedia Appendix 8.2: Variable set: Individual baseline-controlled mean

#### t-1

| Table S21: Estimates for Mixed effects model on FS for t-1 using baseline-controlled mean; blood pressure and peripheral oxygen saturation removed from set of variables | | | | |
| --- | --- | --- | --- | --- |
| formula : Value of Finnegan-Score ~ Individual baseline-controlled heart rate in t-1 period + Individual baseline-controlled respiratory rate in t-1 period + Individual baseline-controlled body temperature within 1 day before Finnegan-Score + Hours between last medication as specified and Finnegan-Score + Percentage of birth weight + Gestational age + NAS-type + ( 1 \| newid ) | | | | |
|  | Estimate | Std. Error | 95%-CI | p-value |
| (Intercept) | 8.87 | 0.92 | [7.01, 10.72] | 0.000 |
| Individual baseline-controlled heart rate in t-1 period | 0.03 | 0.00 | [0.03, 0.04] | 0.000 |
| Individual baseline-controlled respiratory rate in t-1 period | 0.01 | 0.00 | [0.00, 0.01] | 0.008 |
| Individual baseline-controlled body temperature within 1 day before Finnegan-Score | 0.15 | 0.10 | [-0.05, 0.36] | 0.142 |
| Hours between last medication as specified and Finnegan-Score | 0.00 | 0.00 | [0.00, 0.00] | 0.000 |
| Percentage of birth weight | 0.00 | 0.00 | [0.00, 0.00] | 0.136 |
| Gestational age | -0.08 | 0.02 | [-0.12, -0.05] | 0.000 |
| Iatrogenic NAS (vs. primary) | 0.53 | 0.63 | [-0.69, 1.76] | 0.398 |

| Table S22: Goodness to fit for Mixed effects model on FS for t-1 using baseline-controlled mean; blood pressure and peripheral oxygen saturation removed from set of variables | |
| --- | --- |
|  |  |
| R2m | 0.07 |
| R2c | 0.27 |
| AIC | 25,992.72 |
| BIC | 26,057.83 |
| Patients | 158.00 |
| Finnegan Scores | 4,971.00 |

#### t-2

| Table S23: Estimates for Mixed effects model on FS for t-1 using baseline-controlled mean; blood pressure and peripheral oxygen saturation removed from set of variables | | | | |
| --- | --- | --- | --- | --- |
| formula : Value of Finnegan-Score ~ Individual baseline-controlled heart rate in t-2 period + Individual baseline-controlled respiratory rate in t-2 period + Individual baseline-controlled body temperature within 1 day before Finnegan-Score + Hours between last medication as specified and Finnegan-Score + Percentage of birth weight + Gestational age + NAS-type + ( 1 \| newid ) | | | | |
|  | Estimate | Std. Error | 95%-CI | p-value |
| (Intercept) | 8.99 | 0.93 | [7.11, 10.84] | 0.000 |
| Individual baseline-controlled heart rate in t-2 period | 0.03 | 0.00 | [0.02, 0.03] | 0.000 |
| Individual baseline-controlled respiratory rate in t-2 period | 0.01 | 0.00 | [0.01, 0.02] | 0.000 |
| Individual baseline-controlled body temperature within 1 day before Finnegan-Score | 0.21 | 0.10 | [0.00, 0.41] | 0.050 |
| Hours between last medication as specified and Finnegan-Score | 0.00 | 0.00 | [0.00, 0.00] | 0.001 |
| Percentage of birth weight | 0.00 | 0.00 | [0.00, 0.00] | 0.260 |
| Gestational age | -0.09 | 0.02 | [-0.12, -0.05] | 0.000 |
| Iatrogenic NAS (vs. primary) | 0.58 | 0.63 | [-0.64, 1.81] | 0.354 |

| Table S24: Goodness to fit for Mixed effects model on FS for t-1 using baseline-controlled mean; blood pressure and peripheral oxygen saturation removed from set of variables | |
| --- | --- |
|  |  |
| R2m | 0.06 |
| R2c | 0.26 |
| AIC | 26,170.33 |
| BIC | 26,235.49 |
| Patients | 158.00 |
| Finnegan Scores | 4,995.00 |

## Multimedia Appendix 9: Mixed effects models without bloodpressure, peripheral_oxygen_saturation, gest_age, body temperature

### Multimedia Appendix 9.1: Variable set: Mean

#### t-1

| Table S25: Estimates for Mixed effects model on FS for t-1 using mean; blood pressure, peripheral oxygen saturation, gestational age and body temperature removed from set of variables | | | | |
| --- | --- | --- | --- | --- |
| formula : Value of Finnegan-Score ~ Mean heart rate in t-1 period + Mean respiratory rate in t-1 period + Hours between last medication as specified and Finnegan-Score + Percentage of birth weight + ( 1 \| NAS_type ) + ( 1 \| newid ) | | | | |
|  | Estimate | Std. Error | 95%-CI | p-value |
| (Intercept) | 1.16 | 0.38 | [0.43, 1.91] | 0.002 |
| Mean heart rate in t-1 period | 0.03 | 0.00 | [0.03, 0.04] | 0.000 |
| Mean respiratory rate in t-1 period | 0.01 | 0.00 | [0.00, 0.02] | 0.000 |
| Hours between last medication as specified and Finnegan-Score | 0.00 | 0.00 | [0.00, 0.00] | 0.001 |
| Percentage of birth weight | 0.00 | 0.00 | [0.00, 0.00] | 0.034 |

| Table S26: Goodness to fit for Mixed effects model on FS for t-1 using mean; blood pressure, peripheral oxygen saturation, gestational age and body temperature removed from set of variables | |
| --- | --- |
|  |  |
| R2m | 0.04 |
| R2c | 0.21 |
| AIC | 33,317.52 |
| BIC | 33,371.62 |
| Patients | 204.00 |
| Finnegan Scores | 6,390.00 |

#### t-2

| Table S27: Estimates for Mixed effects model on FS for t-2 using mean; blood pressure, peripheral oxygen saturation, gestational age and body temperature removed from set of variables | | | | |
| --- | --- | --- | --- | --- |
| formula : Value of Finnegan-Score ~ Mean heart rate in t-2 period + Mean respiratory rate in t-2 period + Hours between last medication as specified and Finnegan-Score + Percentage of birth weight + ( 1 \| NAS_type ) + ( 1 \| newid ) | | | | |
|  | Estimate | Std. Error | 95%-CI | p-value |
| (Intercept) | 1.50 | 0.39 | [0.74, 2.27] | 0.000 |
| Mean heart rate in t-2 period | 0.03 | 0.00 | [0.02, 0.03] | 0.000 |
| Mean respiratory rate in t-2 period | 0.01 | 0.00 | [0.01, 0.02] | 0.000 |
| Hours between last medication as specified and Finnegan-Score | 0.00 | 0.00 | [0.00, 0.00] | 0.002 |
| Percentage of birth weight | 0.00 | 0.00 | [0.00, 0.00] | 0.078 |

| Table S28: Goodness to fit for Mixed effects model on FS for t-2 using mean; blood pressure, peripheral oxygen saturation, gestational age and body temperature removed from set of variables | |
| --- | --- |
|  |  |
| R2m | 0.03 |
| R2c | 0.21 |
| AIC | 33,439.78 |
| BIC | 33,493.91 |
| Patients | 204.00 |
| Finnegan Scores | 6,411.00 |

### Multimedia Appendix 9.2: Variable set: Individual baseline-controlled mean

#### t-1

| Table S29: Estimates for Mixed effects model on FS for t-1 using baseline-controlled mean; blood pressure, peripheral oxygen saturation, gestational age and body temperature removed from set of variables | | | | |
| --- | --- | --- | --- | --- |
| formula : Value of Finnegan-Score ~ Individual baseline-controlled heart rate in t-1 period + Individual baseline-controlled respiratory rate in t-1 period + Hours between last medication as specified and Finnegan- Score + Percentage of birth weight + ( 1 \| NAS_type ) + ( 1 \| newid ) | | | | |
|  | Estimate | Std. Error | 95%-CI | p-value |
| (Intercept) | 5.93 | 0.17 | [5.42, 6.3] | 0.000 |
| Individual baseline-controlled heart rate in t-1 period | 0.03 | 0.00 | [0.03, 0.04] | 0.000 |
| Individual baseline-controlled respiratory rate in t-1 period | 0.01 | 0.00 | [0.00, 0.01] | 0.006 |
| Hours between last medication as specified and Finnegan-Score | 0.00 | 0.00 | [0.00, 0.00] | 0.000 |
| Percentage of birth weight | 0.00 | 0.00 | [0.00, 0.00] | 0.278 |

| Table S30: Goodness to fit for Mixed effects model on FS for t-1 using baseline-controlled mean; blood pressure, peripheral oxygen saturation, gestational age and body temperature removed from set of variables | |
| --- | --- |
|  |  |
| R2m | 0.05 |
| R2c | 0.22 |
| AIC | 27,623.80 |
| BIC | 27,676.39 |
| Patients | 164.00 |
| Finnegan Scores | 5,292.00 |

#### t-2

| Table S31: Estimates for Mixed effects model on FS for t-2 using baseline-controlled mean; blood pressure, peripheral oxygen saturation, gestational age and body temperature removed from set of variables | | | | |
| --- | --- | --- | --- | --- |
| formula : Value of Finnegan-Score ~ Individual baseline-controlled heart rate in t-2 period + Individual baseline-controlled respiratory rate in t-2 period + Hours between last medication as specified and Finnegan- Score + Percentage of birth weight + ( 1 \| NAS_type ) + ( 1 \| newid ) | | | | |
|  | Estimate | Std. Error | 95%-CI | p-value |
| (Intercept) | 5.91 | 0.17 | [5.4, 6.29] | 0.000 |
| Individual baseline-controlled heart rate in t-2 period | 0.03 | 0.00 | [0.03, 0.04] | 0.000 |
| Individual baseline-controlled respiratory rate in t-2 period | 0.01 | 0.00 | [0.01, 0.02] | 0.000 |
| Hours between last medication as specified and Finnegan-Score | 0.00 | 0.00 | [0.00, 0.00] | 0.000 |
| Percentage of birth weight | 0.00 | 0.00 | [0.00, 0.00] | 0.467 |

| Table S32: Goodness to fit for Mixed effects model on FS for t-2 using baseline-controlled mean; blood pressure, peripheral oxygen saturation, gestational age and body temperature removed from set of variables | |
| --- | --- |
|  |  |
| R2m | 0.04 |
| R2c | 0.21 |
| AIC | 27,809.52 |
| BIC | 27,862.15 |
| Patients | 164.00 |
| Finnegan Scores | 5,318.00 |

## Multimedia Appendix 10: Mixed effects models without bloodpressure, peripheral_oxygen_saturation, gestational age, body temperature, percentage of body weight

### Multimedia Appendix 10.1: Variable set: Mean

#### t-1

| Table S33: Estimates for Mixed effects model on FS for t-1 using mean; blood pressure, peripheral oxygen saturation, gestational age, body temperature and percentage of birth weight removed from set of variables | | | | |
| --- | --- | --- | --- | --- |
| formula : Value of Finnegan-Score ~ Mean heart rate in t-1 period + Mean respiratory rate in t-1 period + Hours between last medication as specified and Finnegan-Score + ( 1 \| NAS_type ) + ( 1 \| newid ) | | | | |
|  | Estimate | Std. Error | 95%-CI | p-value |
| (Intercept) | 0.91 | 0.37 | [0.19, 1.63] | 0.013 |
| Mean heart rate in t-1 period | 0.03 | 0.00 | [0.03, 0.04] | 0.000 |
| Mean respiratory rate in t-1 period | 0.01 | 0.00 | [0.00, 0.02] | 0.000 |
| Hours between last medication as specified and Finnegan-Score | 0.00 | 0.00 | [0.00, 0.00] | 0.000 |

| Table S34: Goodness to fit for Mixed effects model on FS for t-1 using mean; blood pressure, peripheral oxygen saturation, gestational age, body temperature and percentage of birth weight removed from set of variables | |
| --- | --- |
|  |  |
| R2m | 0.04 |
| R2c | 0.22 |
| AIC | 34,495.20 |
| BIC | 34,542.78 |
| Patients | 204.00 |
| Finnegan Scores | 6,623.00 |

#### t-2

| Table S35: Estimates for Mixed effects model on FS for t-2 using mean; blood pressure, peripheral oxygen saturation, gestational age, body temperature and percentage of birth weight removed from set of variables | | | | |
| --- | --- | --- | --- | --- |
| formula : Value of Finnegan-Score ~ Mean heart rate in t-2 period + Mean respiratory rate in t-2 period + Hours between last medication as specified and Finnegan-Score + ( 1 \| NAS_type ) + ( 1 \| newid ) | | | | |
|  | Estimate | Std. Error | 95%-CI | p-value |
| (Intercept) | 1.34 | 0.38 | [0.61, 2.09] | 0.000 |
| Mean heart rate in t-2 period | 0.03 | 0.00 | [0.02, 0.03] | 0.000 |
| Mean respiratory rate in t-2 period | 0.01 | 0.00 | [0.01, 0.02] | 0.000 |
| Hours between last medication as specified and Finnegan-Score | 0.00 | 0.00 | [0.00, 0.00] | 0.001 |

| Table S36: Goodness to fit for Mixed effects model on FS for t-2 using mean; blood pressure, peripheral oxygen saturation, gestational age, body temperature and percentage of birth weight removed from set of variables | |
| --- | --- |
|  |  |
| R2m | 0.03 |
| R2c | 0.21 |
| AIC | 34,648.38 |
| BIC | 34,696.00 |
| Patients | 204.00 |
| Finnegan Scores | 6,646.00 |

### Multimedia Appendix 10.2: Variable set: Individual baseline-controlled mean

#### t-1

| Table S37: Estimates for Mixed effects model on FS for t-1 using baseline-controlled mean; blood pressure, peripheral oxygen saturation, gestational age, body temperature and percentage of birth weight removed from set of variables | | | | |
| --- | --- | --- | --- | --- |
| formula : Value of Finnegan-Score ~ Individual baseline-controlled heart rate in t-1 period + Individual baseline-controlled respiratory rate in t-1 period + Hours between last medication as specified and Finnegan-Score + ( 1 \| NAS_type ) + ( 1 \| newid ) | | | | |
|  | Estimate | Std. Error | 95%-CI | p-value |
| (Intercept) | 5.81 | 0.14 | [5.33, 6.17] | 0.000 |
| Individual baseline-controlled heart rate in t-1 period | 0.04 | 0.00 | [0.03, 0.04] | 0.000 |
| Individual baseline-controlled respiratory rate in t-1 period | 0.01 | 0.00 | [0.00, 0.01] | 0.004 |
| Hours between last medication as specified and Finnegan-Score | 0.00 | 0.00 | [0.00, 0.00] | 0.000 |

| Table S38: Goodness to fit for Mixed effects model on FS for t-1 using baseline-controlled mean; blood pressure, peripheral oxygen saturation, gestational age, body temperature and percentage of birth weight removed from set of variables | |
| --- | --- |
|  |  |
| R2m | 0.05 |
| R2c | 0.22 |
| AIC | 28,573.20 |
| BIC | 28,619.46 |
| Patients | 164.00 |
| Finnegan Scores | 5,482.00 |

#### t-2

| Table S39: Estimates for Mixed effects model on FS for t-2 using mean; blood pressure, peripheral oxygen saturation, gestational age, body temperature and percentage of birth weight removed from set of variables | | | | |
| --- | --- | --- | --- | --- |
| formula : Value of Finnegan-Score ~ Individual baseline-controlled heart rate in t-2 period + Individual baseline-controlled respiratory rate in t-2 period + Hours between last medication as specified and Finnegan-Score + ( 1 \| NAS_type ) + ( 1 \| newid ) | | | | |
|  | Estimate | Std. Error | 95%-CI | p-value |
| (Intercept) | 5.83 | 0.14 | [5.35, 6.19] | 0 |
| Individual baseline-controlled heart rate in t-2 period | 0.03 | 0.00 | [0.03, 0.04] | 0 |
| Individual baseline-controlled respiratory rate in t-2 period | 0.01 | 0.00 | [0.01, 0.02] | 0 |
| Hours between last medication as specified and Finnegan-Score | 0.00 | 0.00 | [0.00, 0.00] | 0 |

| Table S40: Goodness to fit for Mixed effects model on FS for t-2 using mean; blood pressure, peripheral oxygen saturation, gestational age, body temperature and percentage of birth weight removed from set of variables | |
| --- | --- |
|  |  |
| R2m | 0.04 |
| R2c | 0.21 |
| AIC | 28,797.27 |
| BIC | 28,843.57 |
| Patients | 164.00 |
| Finnegan Scores | 5,512.00 |

## Multimedia Appendix 11: Model fit comparison including reworked analysis

| Table S41: Goodness to fit parameters, patient and score counts of mixed effects models | | | | | | |
| --- | --- | --- | --- | --- | --- | --- |
|  | R2m | R2c | AIC | BIC | Patients | Finnegan Scores |
| Mixed effects model on FS for t-1 using mean; full set of variables | 0.10 | 0.37 | 2,308.997 | 2,357.423 | 102 | 418 |
| Mixed effects model on FS for t-2 using mean; full set of variables | 0.10 | 0.31 | 2,340.328 | 2,388.925 | 101 | 424 |
| Mixed effects model on FS for t-1 using baseline-controlled mean; full set of variables | 0.11 | 0.43 | 1,999.361 | 2,045.894 | 84 | 357 |
| Mixed effects model on FS for t-2 using baseline-controlled mean; full set of variables | 0.09 | 0.38 | 2,010.923 | 2,057.589 | 83 | 361 |
| Mixed effects model on FS for t-1 using mean; body temperature removed from set of variables | 0.10 | 0.37 | 2,308.320 | 2,352.710 | 102 | 418 |
| Mixed effects model on FS for t-2 using mean; body temperature removed from set of variables | 0.08 | 0.32 | 2,345.508 | 2,390.055 | 101 | 424 |
| Mixed effects model on FS for t-1 using baseline-controlled mean; body temperature removed from set of variables | 0.11 | 0.42 | 2,008.566 | 2,051.283 | 85 | 359 |
| Mixed effects model on FS for t-1 using baseline-controlled mean; body temperature removed from set of variables. | 0.09 | 0.38 | 2,024.581 | 2,067.450 | 84 | 364 |
| Mixed effects model on FS for t-1 using mean; blood pressure and peripheral oxygen saturation removed from set of variables | 0.06 | 0.25 | 31,753.239 | 31,820.371 | 199 | 6,083 |
| Mixed effects model on FS for t-2 using mean; blood pressure and peripheral oxygen saturation removed from set of variables | 0.05 | 0.25 | 31,878.797 | 31,945.966 | 199 | 6,105 |
| Mixed effects model on FS for t-1 using baseline-controlled mean; blood pressure and peripheral oxygen saturation removed from set of variables | 0.07 | 0.27 | 25,992.717 | 26,057.831 | 158 | 4,971 |
| Mixed effects model on FS for t-1 using baseline-controlled mean; blood pressure and peripheral oxygen saturation removed from set of variables. | 0.06 | 0.26 | 26,170.331 | 26,235.492 | 158 | 4,995 |
| Mixed effects model on FS for t-1 using mean; blood pressure, peripheral oxygen saturation, gestational age and body temperature removed from set of variables | 0.04 | 0.21 | 33,317.520 | 33,371.620 | 204 | 6,390 |
| Mixed effects model on FS for t-2 using mean; blood pressure, peripheral oxygen saturation, gestational age and body temperature removed from set of variables | 0.03 | 0.21 | 33,439.782 | 33,493.908 | 204 | 6,411 |
| Mixed effects model on FS for t-1 using baseline-controlled mean; blood pressure, peripheral oxygen saturation, gestational age and body temperature removed from set of variables | 0.05 | 0.22 | 27,623.796 | 27,676.388 | 164 | 5,292 |
| Mixed effects model on FS for t-2 using baseline-controlled mean; blood pressure, peripheral oxygen saturation, gestational age and body temperature removed from set of variables | 0.04 | 0.21 | 27,809.515 | 27,862.146 | 164 | 5,318 |
| Mixed effects model on FS for t-1 using mean; blood pressure, peripheral oxygen saturation, gestational age, body temperature and percentage of birth weight removed from set of variables | 0.04 | 0.22 | 34,495.196 | 34,542.784 | 204 | 6,623 |
| Mixed effects model on FS for t-2 using mean; blood pressure, peripheral oxygen saturation, gestational age, body temperature and percentage of birth weight removed from set of variables | 0.03 | 0.21 | 34,648.383 | 34,695.995 | 204 | 6,646 |
| Mixed effects model on FS for t-1 using baseline-controlled mean; blood pressure, peripheral oxygen saturation, gestational age, body temperature and percentage of birth weight removed from set of variables | 0.05 | 0.22 | 28,573.200 | 28,619.464 | 164 | 5,482 |
| Mixed effects model on FS for t-2 using mean; blood pressure, peripheral oxygen saturation, gestational age, body temperature and percentage of birth weight removed from set of variables. | 0.04 | 0.21 | 28,797.271 | 28,843.574 | 164 | 5,512 |
