# Supplementary material for: The Finnegan Score for Neonatal Opioid Withdrawal Revisited With Routine Electronic Data: Retrospective Study
Source: JMIR Pediatr Parent. 2024 Feb 28;7:e50575. doi: 10.2196/50575 (PMC11004517; doi:10.2196/50575)
Supplement: Multimedia Appendix 2 [file pediatrics-v7-e50575-s002.docx]

Multimedia Appendix to:
Finnegan score revisited by routine electronic data - sensitivity analysis

Till Rech, Kerstin Rubarth, Christoph Bührer, Felix Balzer, Christof Dame

2023-10-24

Table of Contents

[Multimedia Appendix 1: Extended patient characteristics table 3](#_Toc151383130)

[Multimedia Appendix 2: Analysis 6](#_Toc151383131)

[Multimedia Appendix 2.1: Diagrams 6](#_Toc151383132)

[Variable counts 6](#_Toc151383133)

[Graphs on heart rate 7](#_Toc151383134)

[Graphs on respiratory rate 9](#_Toc151383135)

[Graphs on peripheral oxygen saturation 11](#_Toc151383136)

[Graphs on mean arterial bloodpressure 13](#_Toc151383137)

[Multimedia Appendix 2.2: Mixed effects models with full set of variables 15](#_Toc151383138)

[Variable set: Mean 15](#_Toc151383139)

[t-1 15](#_Toc151383140)

[t-2 16](#_Toc151383141)

[Variable set: Individual baseline-controlled mean 17](#_Toc151383142)

[t-1 17](#_Toc151383143)

[t-2 19](#_Toc151383144)

[Multimedia Appendix 2.3: Mixed effects model without body temperature 21](#_Toc151383145)

[Variable set: Mean 21](#_Toc151383146)

[t-1 21](#_Toc151383147)

[t-2 22](#_Toc151383148)

[Variable set: Individual baseline-controlled mean 23](#_Toc151383149)

[t-1 23](#_Toc151383150)

[t-2 24](#_Toc151383151)

[Multimedia Appendix 2.4: Mixed effects model without bloodpressure, peripheral_oxygen_saturation 25](#_Toc151383152)

[Variable set: Mean 25](#_Toc151383153)

[t-1 25](#_Toc151383154)

[t-2 26](#_Toc151383155)

[Variable set: Individual baseline-controlled mean 27](#_Toc151383156)

[t-1 27](#_Toc151383157)

[t-2 28](#_Toc151383158)

[Multimedia Appendix 2.5: Mixed effects models without bloodpressure, peripheral_oxygen_saturation, gest_age, body temperature 29](#_Toc151383159)

[Variable set: Mean 29](#_Toc151383160)

[t-1 29](#_Toc151383161)

[t-2 30](#_Toc151383162)

[Variable set: Individual baseline-controlled mean 31](#_Toc151383163)

[t-1 31](#_Toc151383164)

[t-2 32](#_Toc151383165)

[Multimedia Appendix 2.6: Mixed effects models without bloodpressure, peripheral_oxygen_saturation, gestational age, body temperature, percentage of body weight 33](#_Toc151383166)

[Variable set: Mean 33](#_Toc151383167)

[t-1 33](#_Toc151383168)

[t-2 34](#_Toc151383169)

[Variable set: Individual baseline-controlled mean 35](#_Toc151383170)

[t-1 35](#_Toc151383171)

[t-2 36](#_Toc151383172)

[Multimedia Appendix 2.7: Model fit comparison including reworked analysis 37](#_Toc151383173)

# Multimedia Appendix 1: Extended patient characteristics table

| Table S1: patient characteristics | | |
| --- | --- | --- |
|  | level | Overall |
| n |  | 491 |
| Sex ( % ) | F | 219 (44.6) |
|  | M | 272 (55.4) |
| Year of birth ( % ) | 2015 | 1 ( 0.2) |
|  | 2016 | 15 ( 3.1) |
|  | 2017 | 89 (18.1) |
|  | 2018 | 74 (15.1) |
|  | 2019 | 113 (23.0) |
|  | 2020 | 113 (23.0) |
|  | 2021 | 85 (17.3) |
|  | 2022 | 1 ( 0.2) |
| Gestational age ( median [IQR] ) |  | 37+5 [34+4.75, 39+3.25] |
| Birthweight ( median [IQR] ) |  | 2,760.00 [2,000.00, 3,210.00] |
| Mode of delivery ( % ) | caesarean section | 256 (52.1) |
|  | vaginal delivery | 186 (37.9) |
|  | Data n/a | 49 (10.0) |
| Caesarean section subcategories ( % ) | unspecified caesarean section | 94 (36.7) |
|  | secondary C-Section | 71 (27.7) |
|  | elective/primary C-Section | 55 (21.5) |
|  | emergency C-Section | 24 ( 9.4) |
|  | urgent C-Section | 12 ( 4.7) |
| Vaginal delivery subcategories ( % ) | spontanous delivery | 153 (82.3) |
|  | Vacuum extraction | 28 (15.1) |
|  | spontanous delivery after labor induction | 4 ( 2.2) |
|  | forceps extraction | 1 ( 0.5) |
| Umbilical cord blood pH ( median [IQR] ) |  | 7.26 [7.18, 7.30] |
| Infant's number of Finnegan score assessments ( median [IQR] ) |  | 19.00 [8.00, 45.50] |
| Infant's average Finnegan score ( median [IQR] ) |  | 6.37 [5.10, 7.73] |
| ICD-10 coded for primary NAS ( % ) | FALSE | 424 (86.4) |
|  | TRUE | 67 (13.6) |
| ICD-10 coded for iatrogenic NAS ( % ) | FALSE | 379 (77.2) |
|  | TRUE | 112 (22.8) |
| ICD-10 coded for any NAS ( % ) | FALSE | 318 (64.8) |
|  | TRUE | 173 (35.2) |
| Time frame for individual baseline ( % ) | definable | 395 (80.4) |
|  | not definable | 96 (19.6) |
| Individual baseline data for heart rate ( % ) | available | 327 (82.8) |
|  | n/a | 68 (17.2) |
| Individual baseline data for respiratory rate ( % ) | available | 323 (81.8) |
|  | n/a | 72 (18.2) |
| Individual baseline data for peripheral oxygen saturation ( % ) | available | 321 (81.3) |
|  | n/a | 74 (18.7) |
| Individual baseline data for mean blood pressure ( % ) | available | 316 (80.0) |
|  | n/a | 79 (20.0) |
| Individual baseline data for diastolic blood pressure ( % ) | available | 316 (80.0) |
|  | n/a | 79 (20.0) |
| Individual baseline data for systolic blood pressure ( % ) | available | 316 (80.0) |
|  | n/a | 79 (20.0) |
| Individual baseline data for body height ( % ) | n/a | 287 (72.7) |
|  | available | 108 (27.3) |
| Individual baseline data for body temperature ( % ) | available | 323 (81.8) |
|  | n/a | 72 (18.2) |
| Individual baseline data for body weight ( % ) | available | 230 (58.2) |
|  | n/a | 165 (41.8) |
| Individual baseline data for FiO2 ( % ) | available | 277 (70.1) |
|  | n/a | 118 (29.9) |

# Multimedia Appendix 2: Analysis

## Multimedia Appendix 2.1: Diagrams

### Variable counts


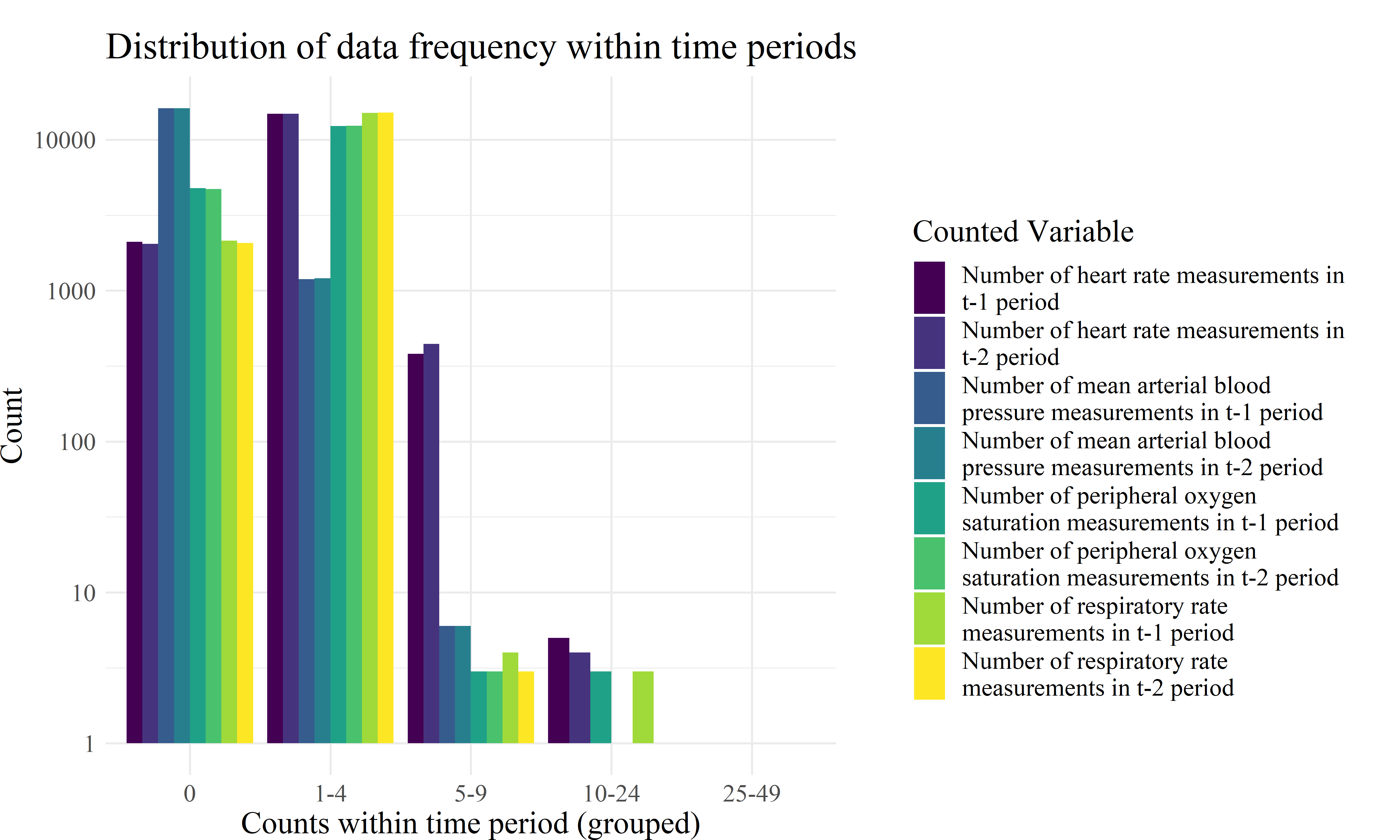


### Graphs on heart rate


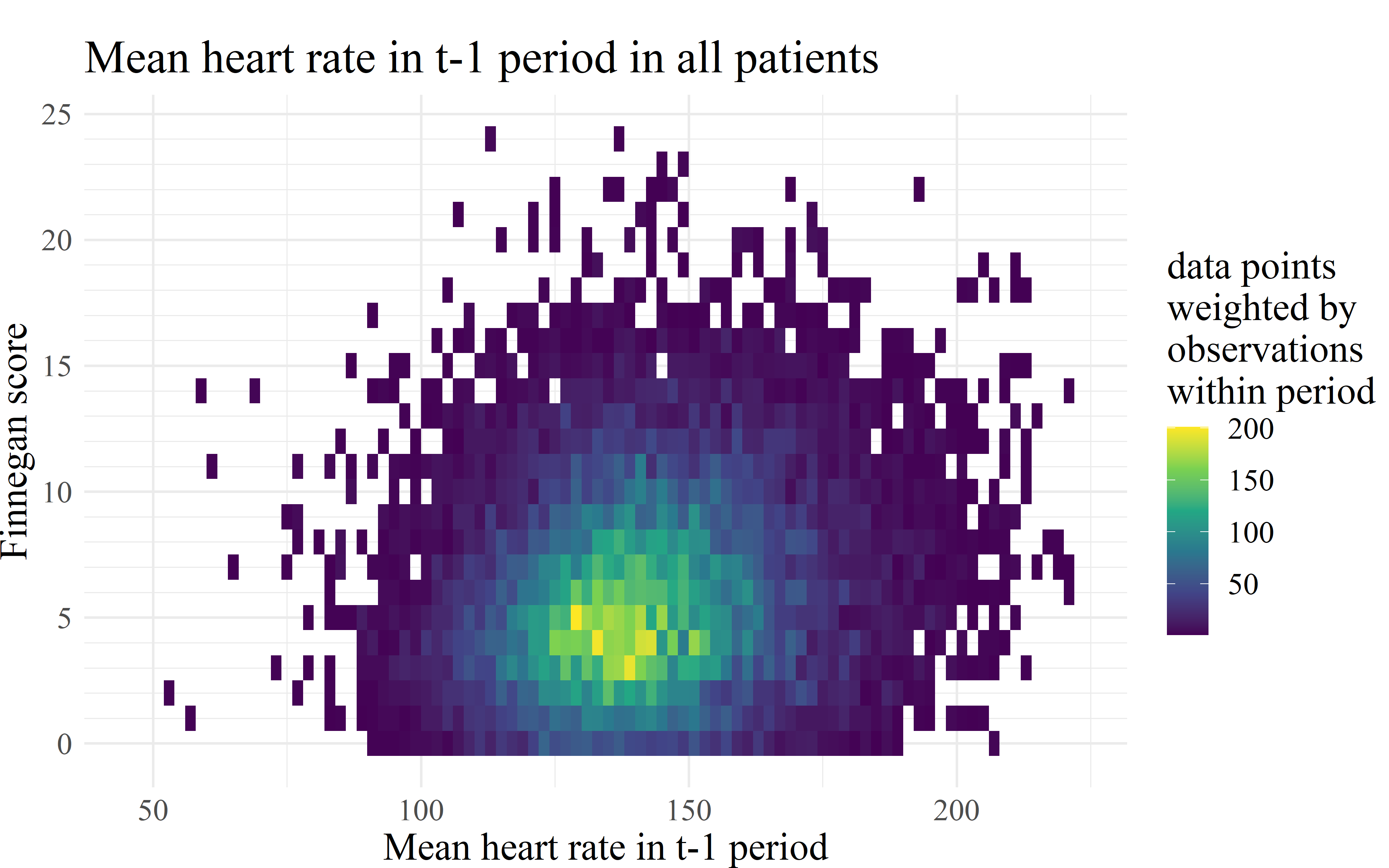

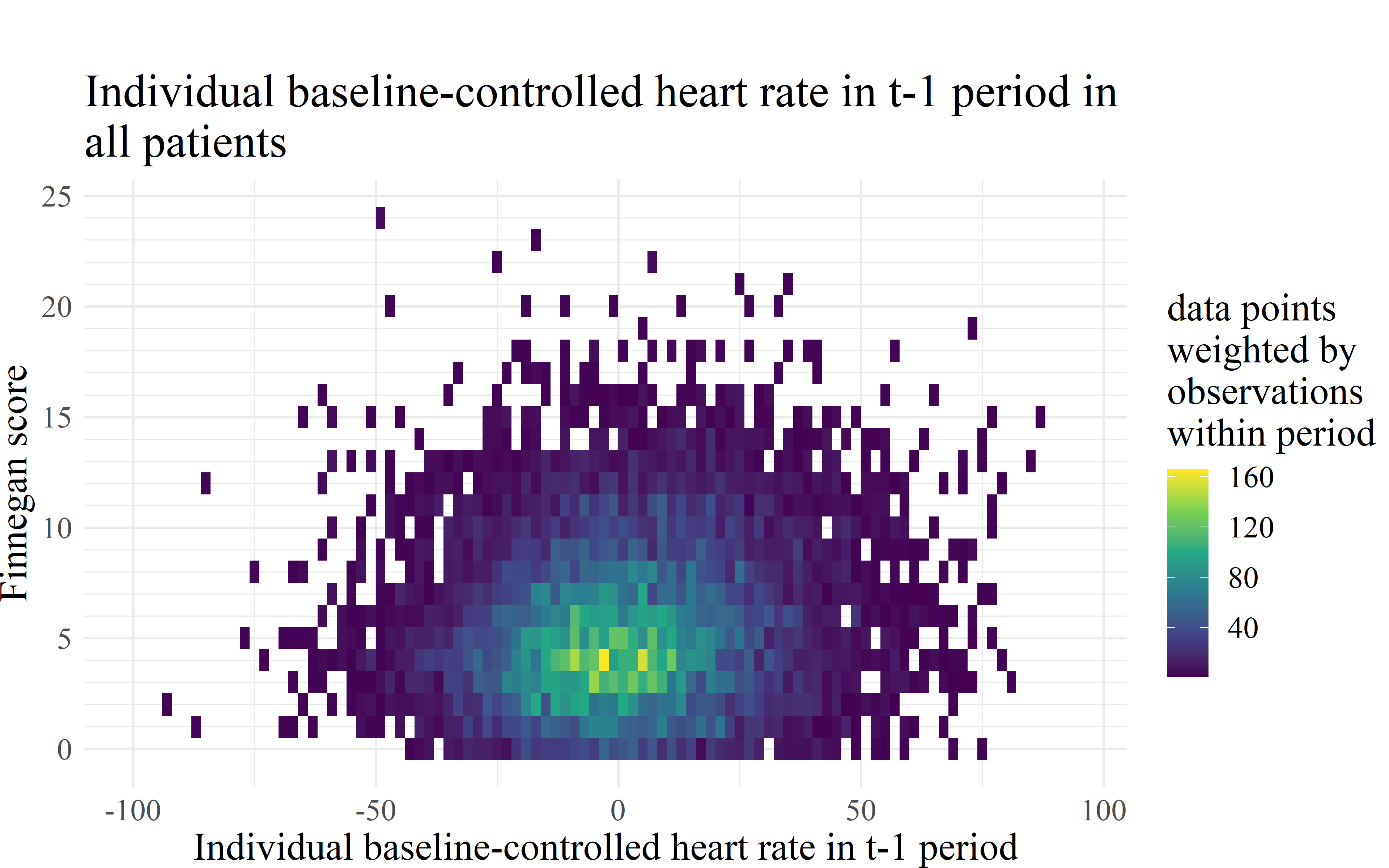

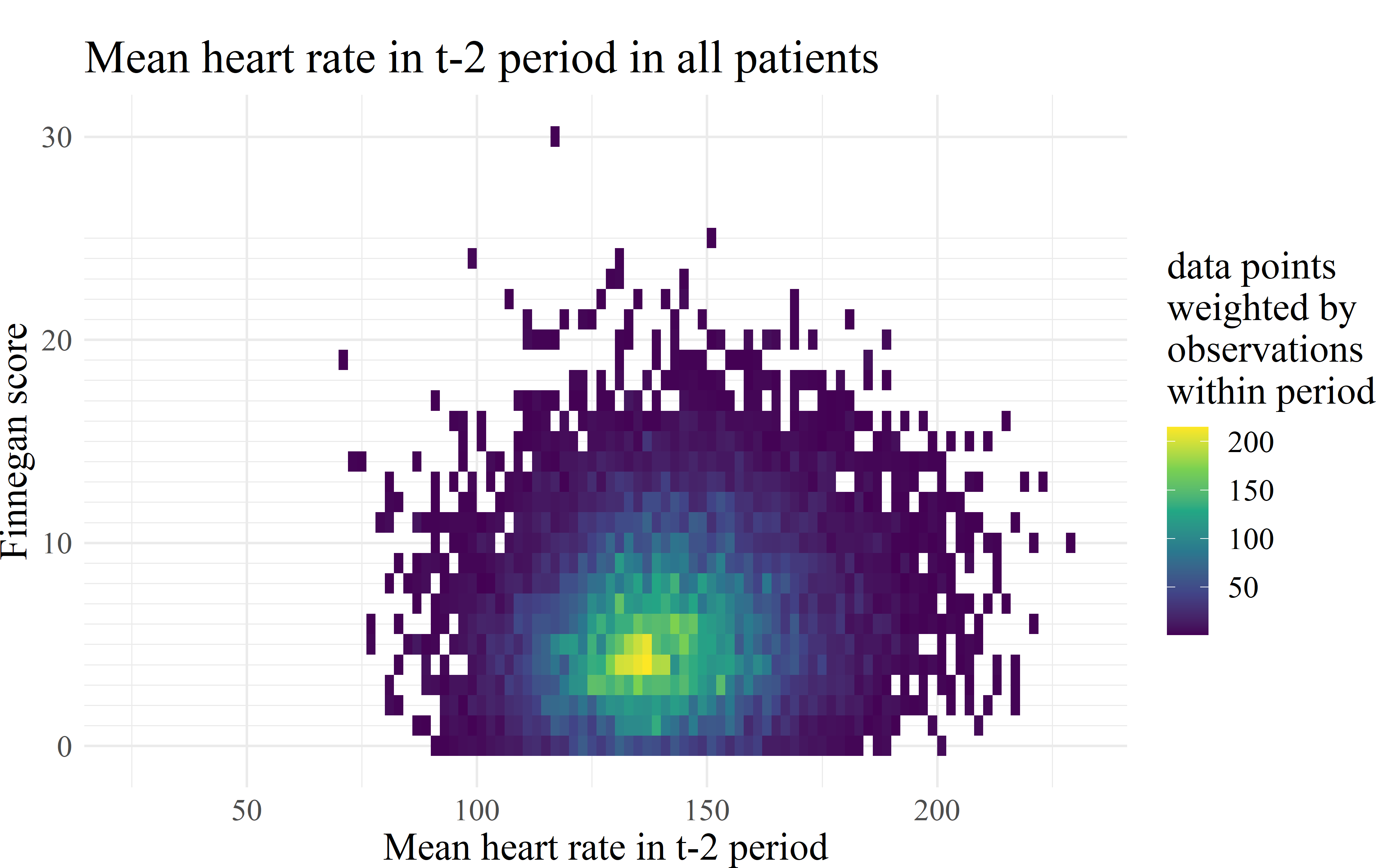

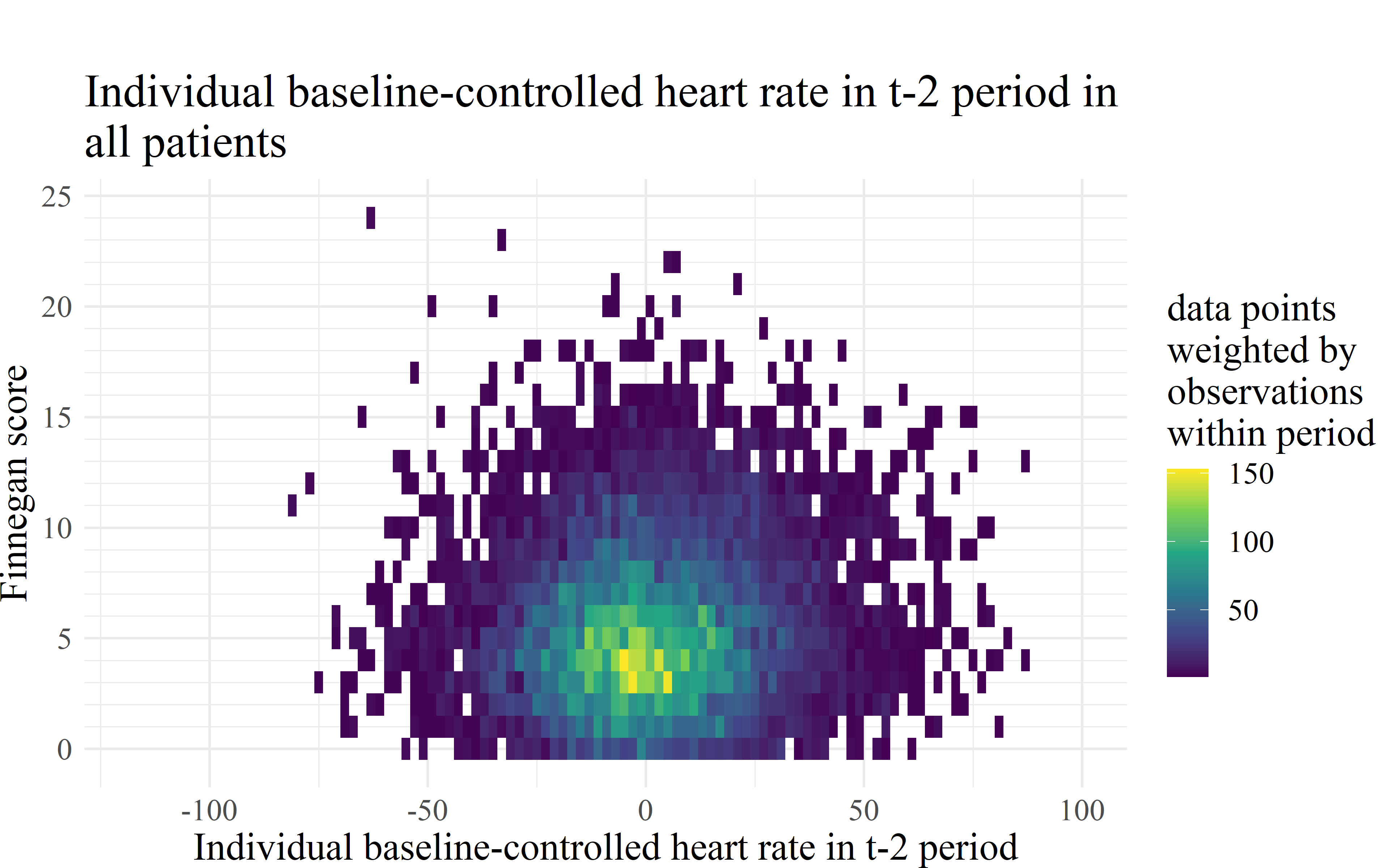


### Graphs on respiratory rate


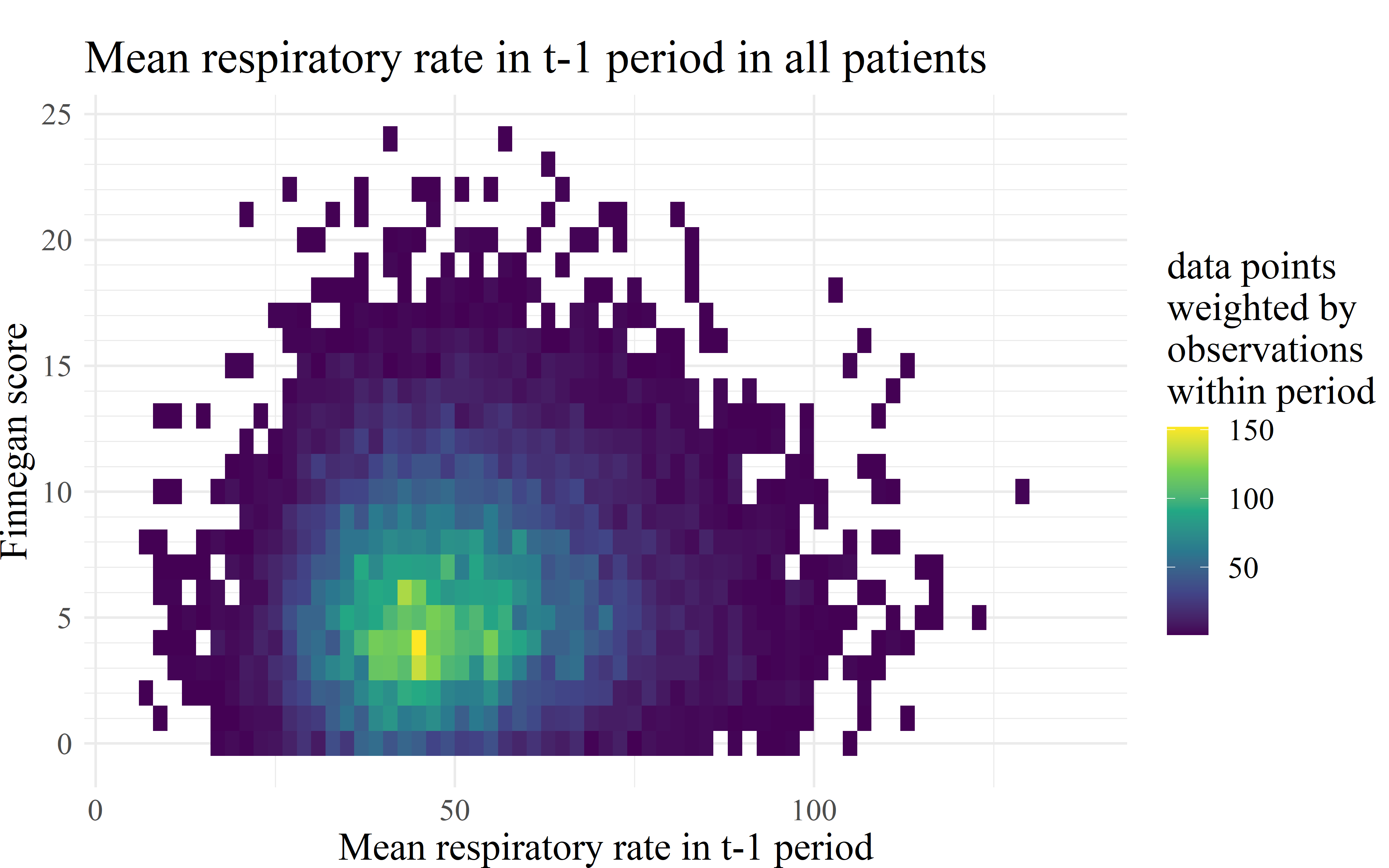

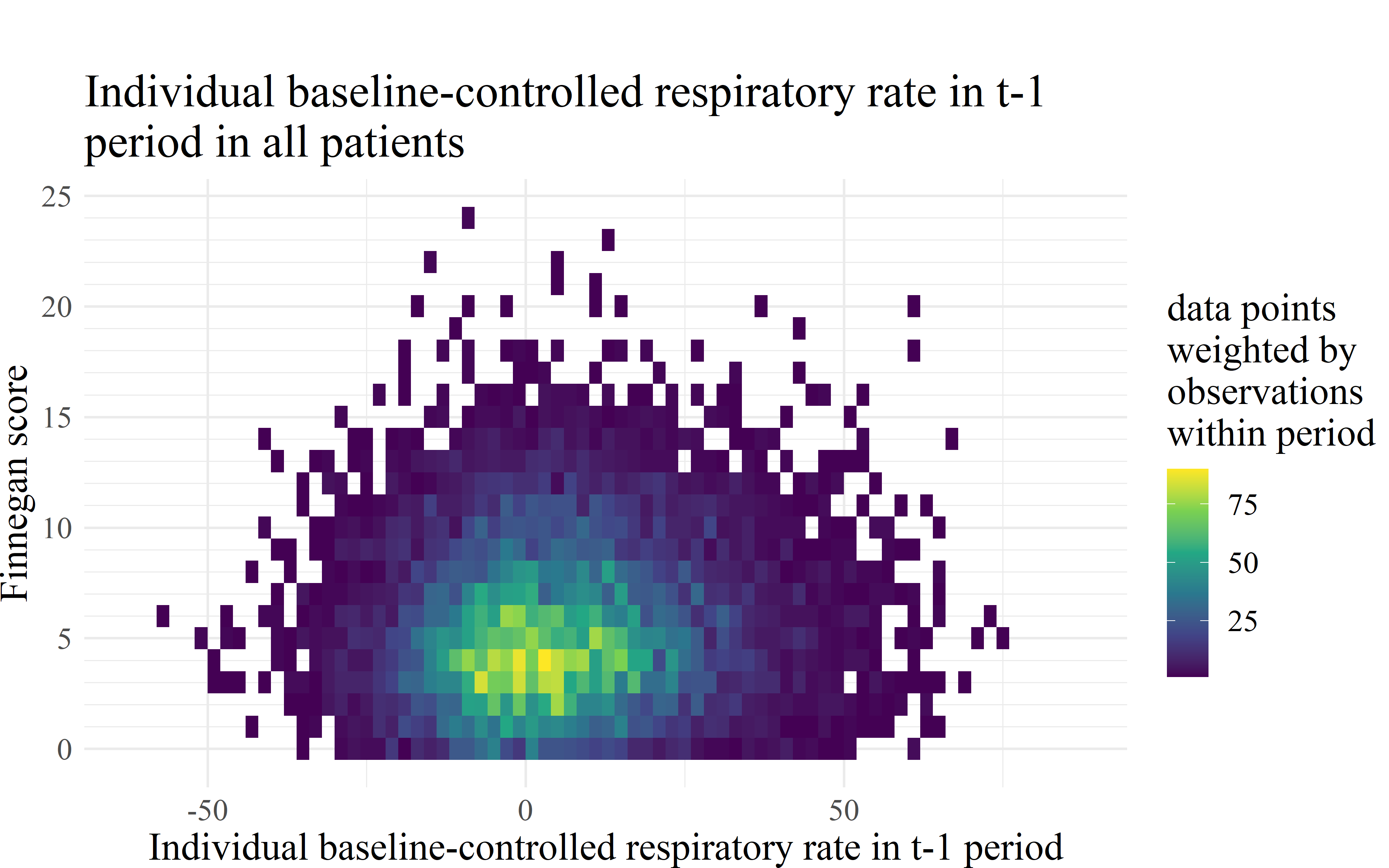

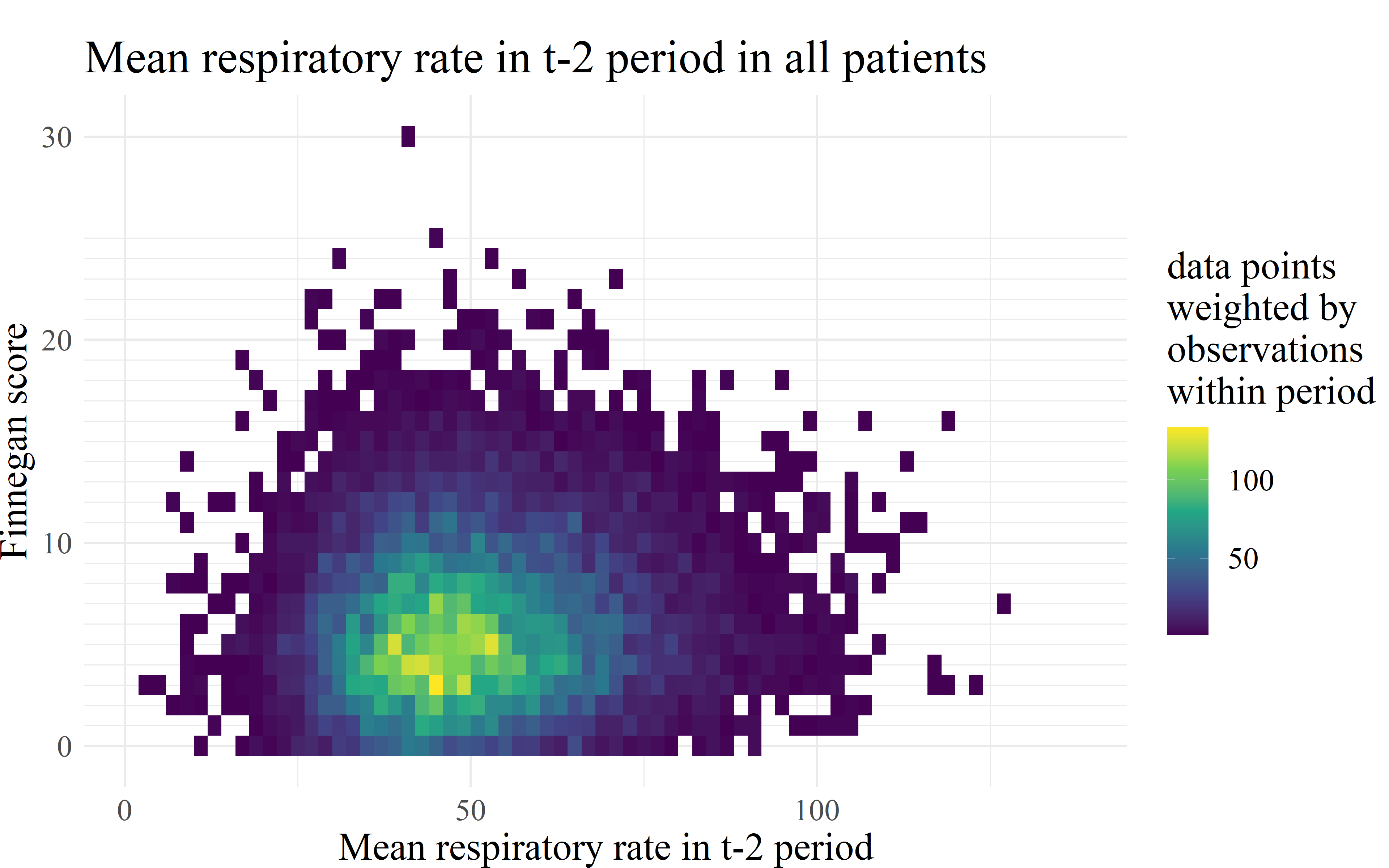

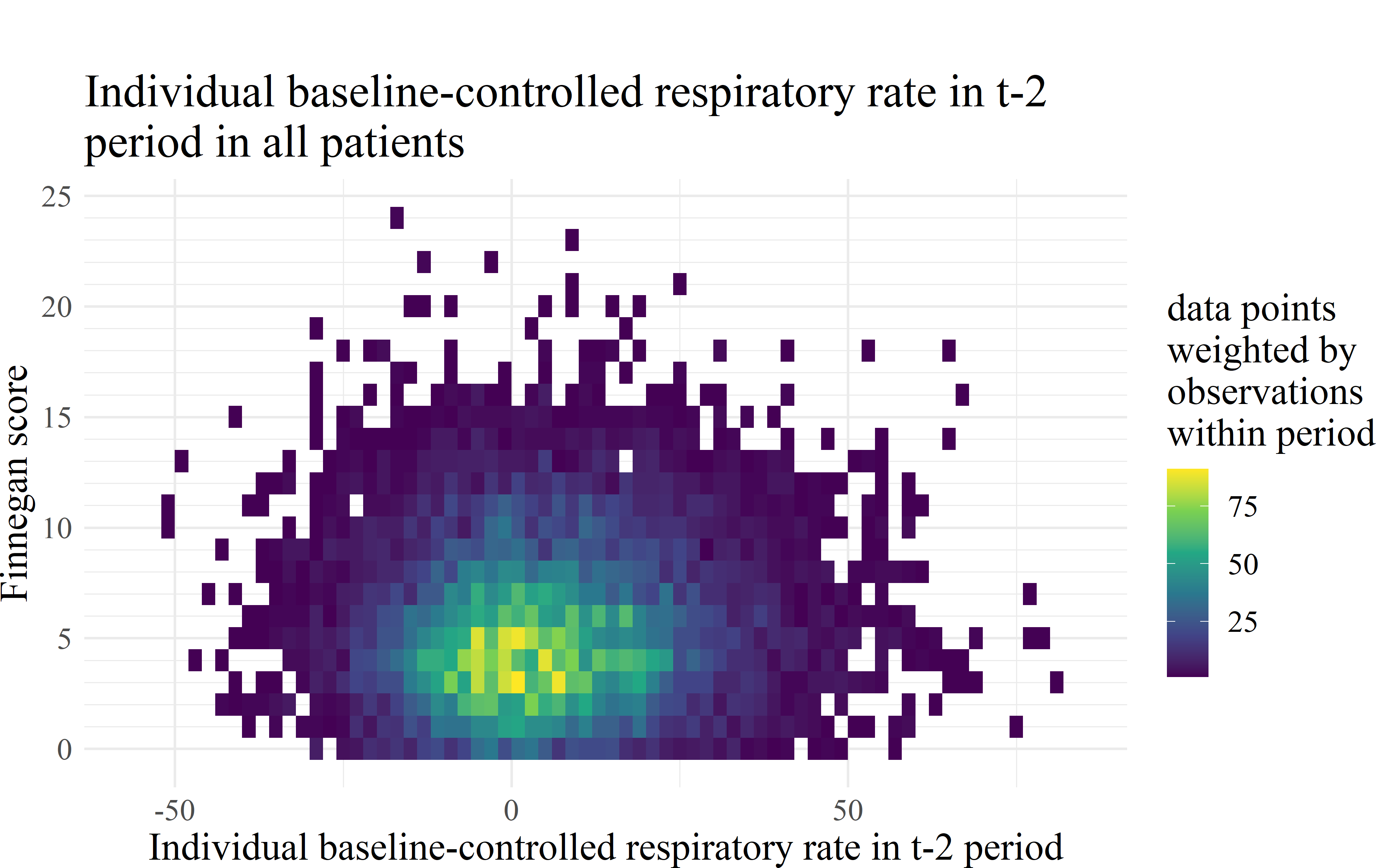


### Graphs on peripheral oxygen saturation


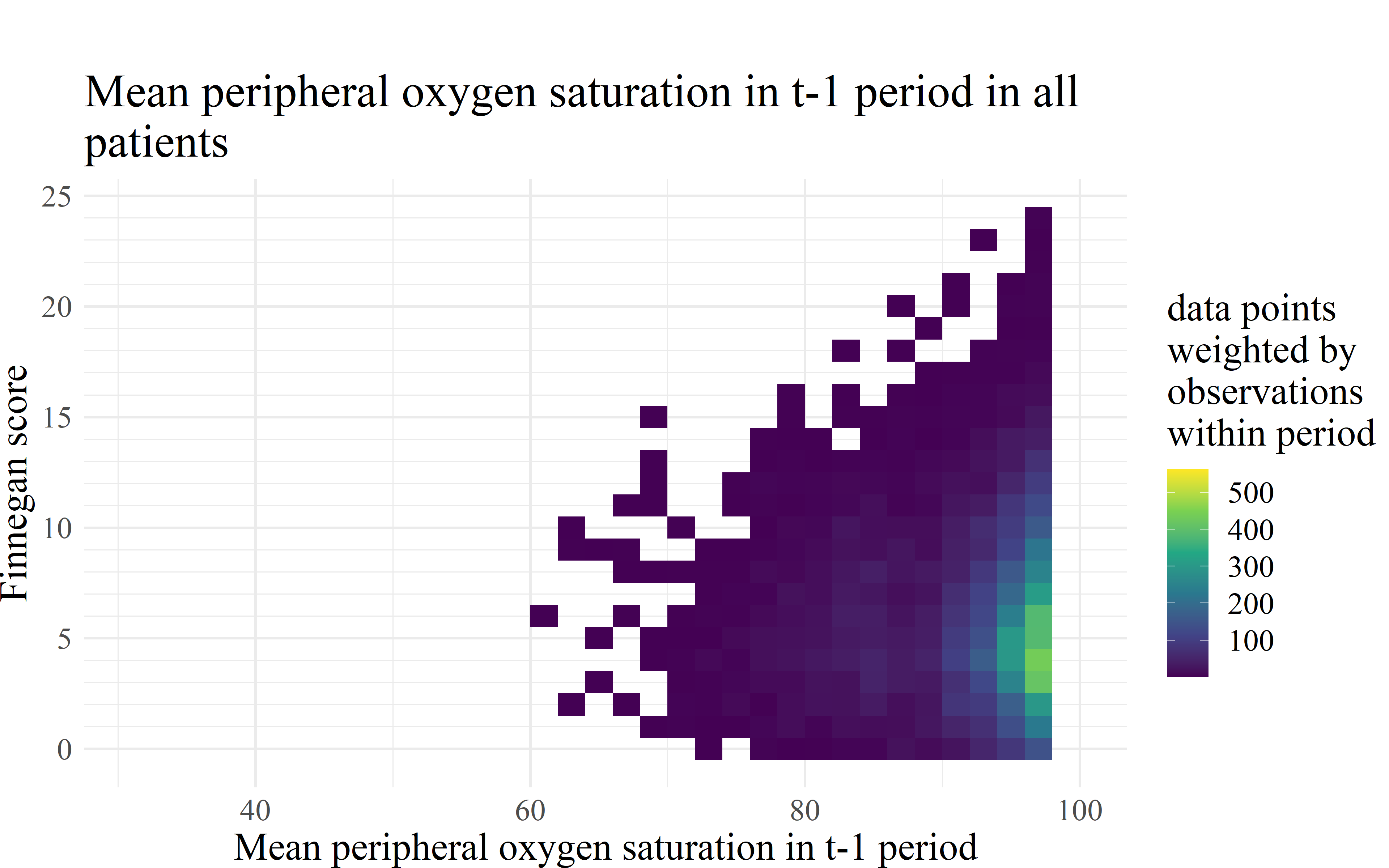

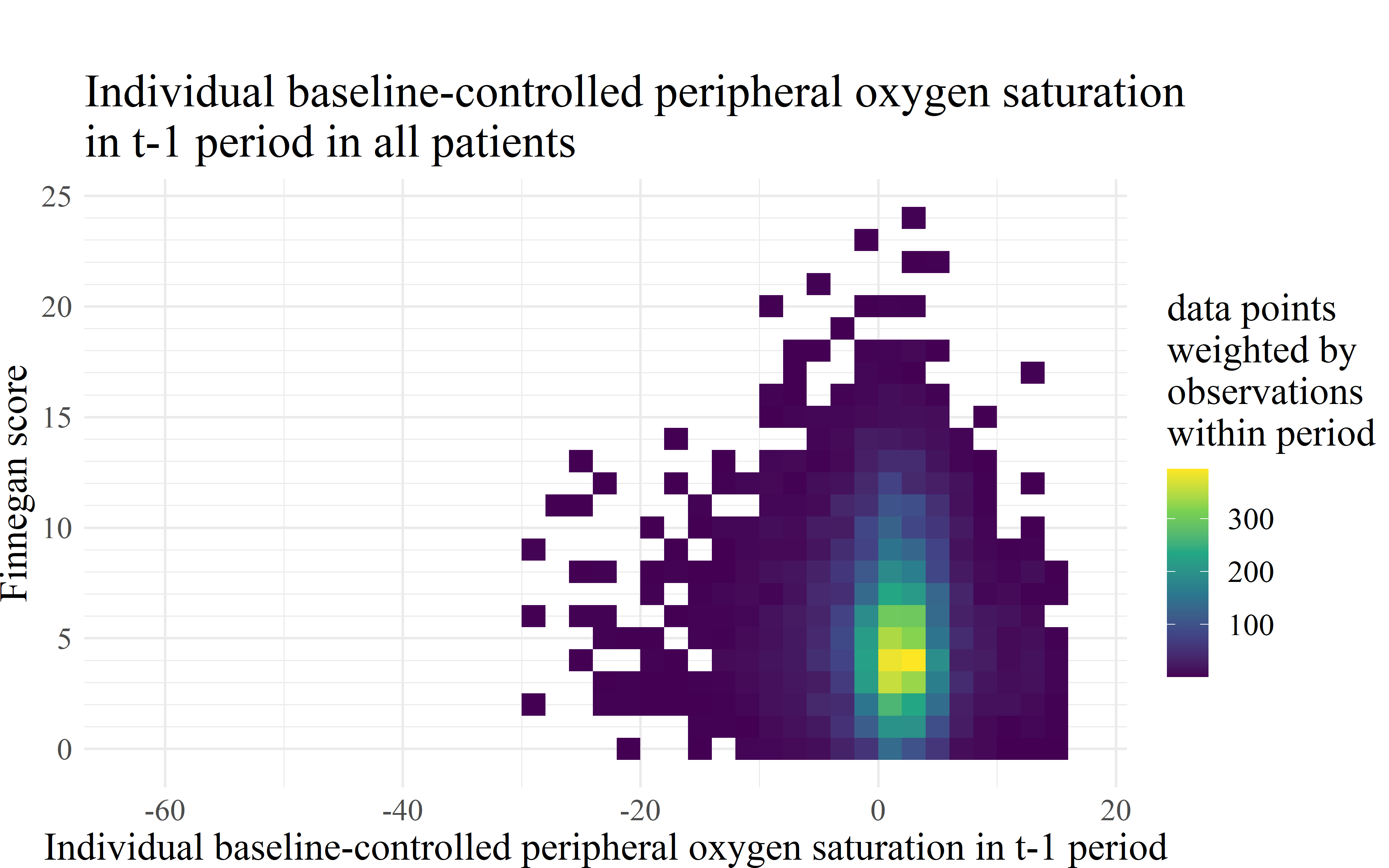

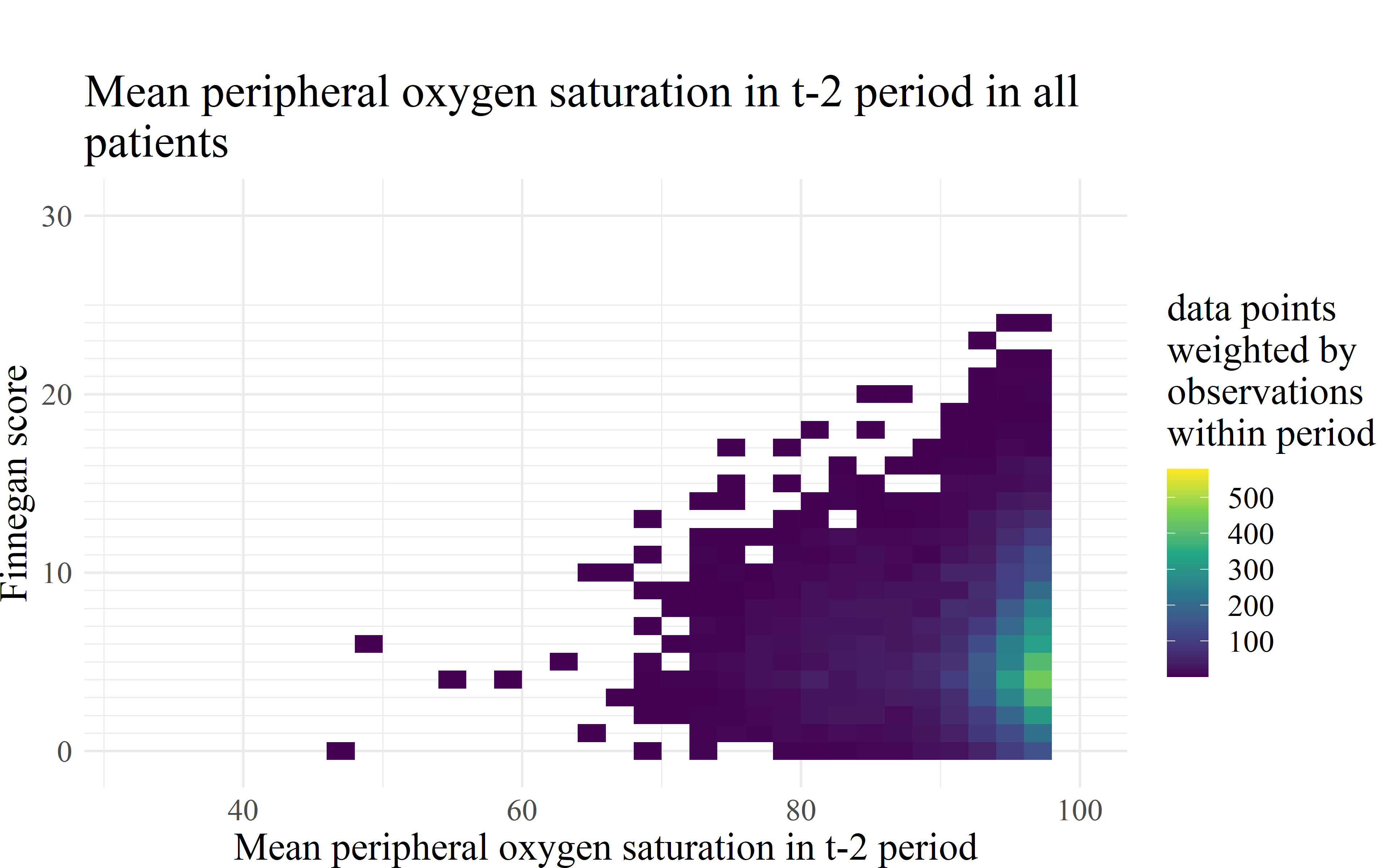

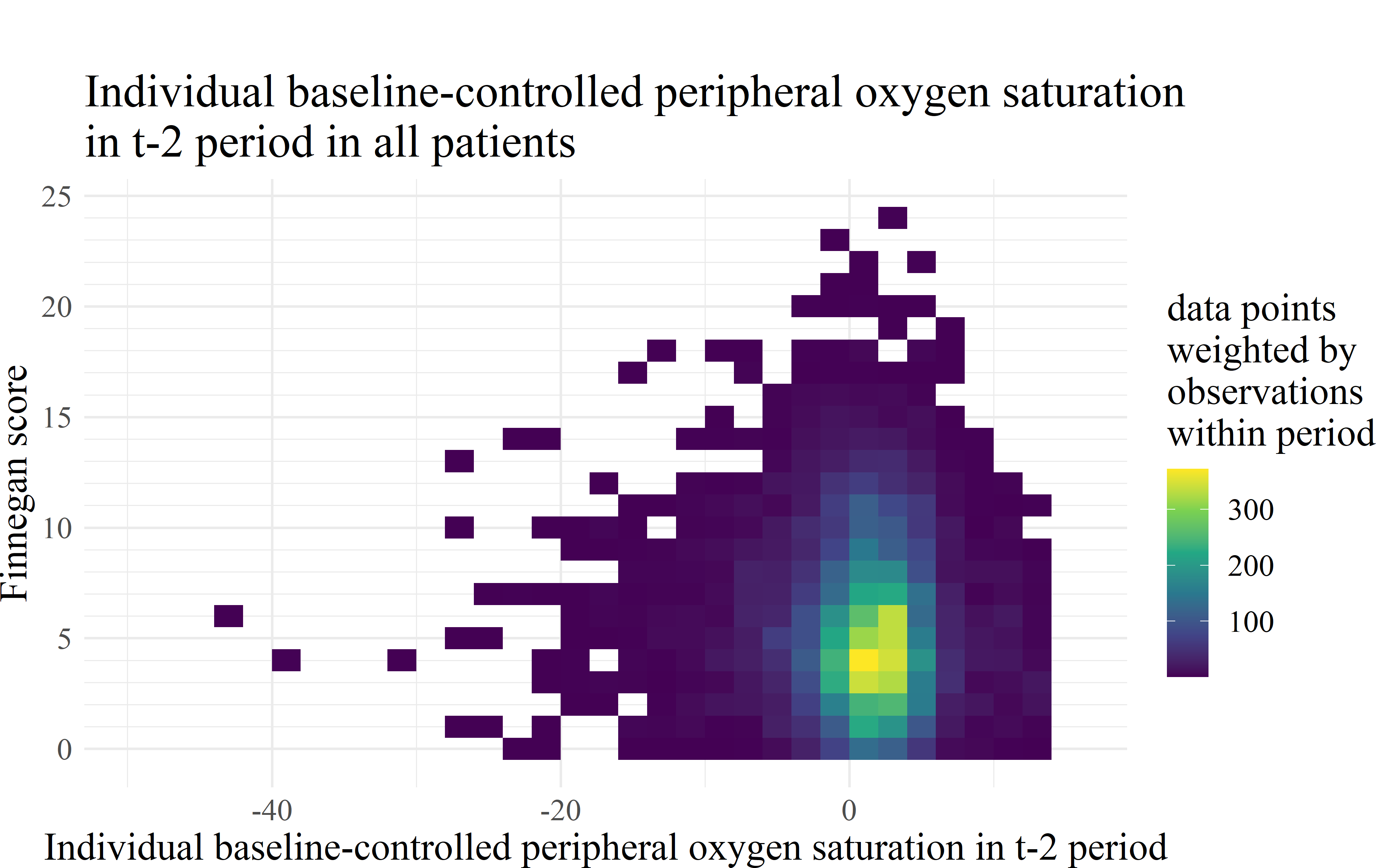


### Graphs on mean arterial bloodpressure


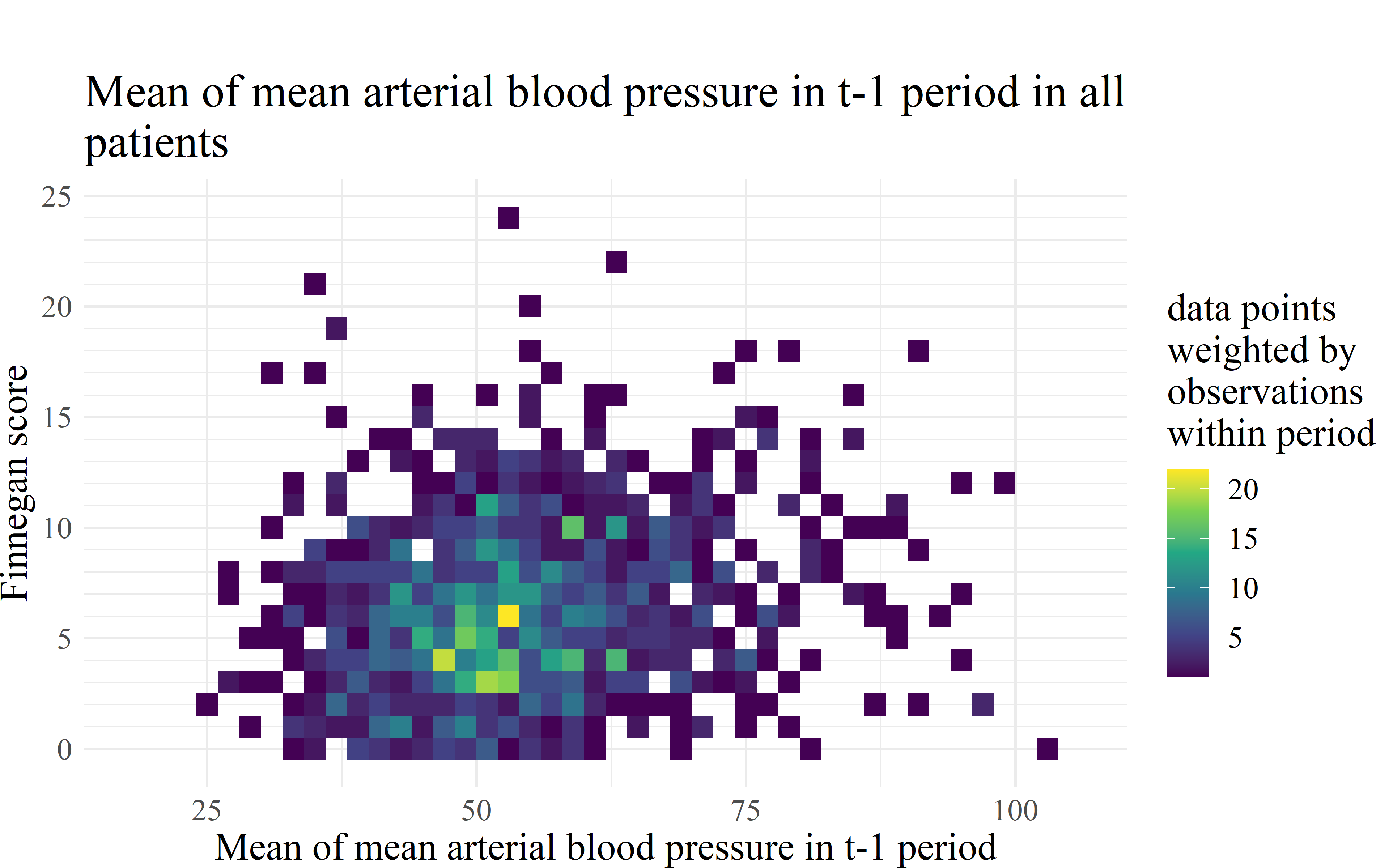

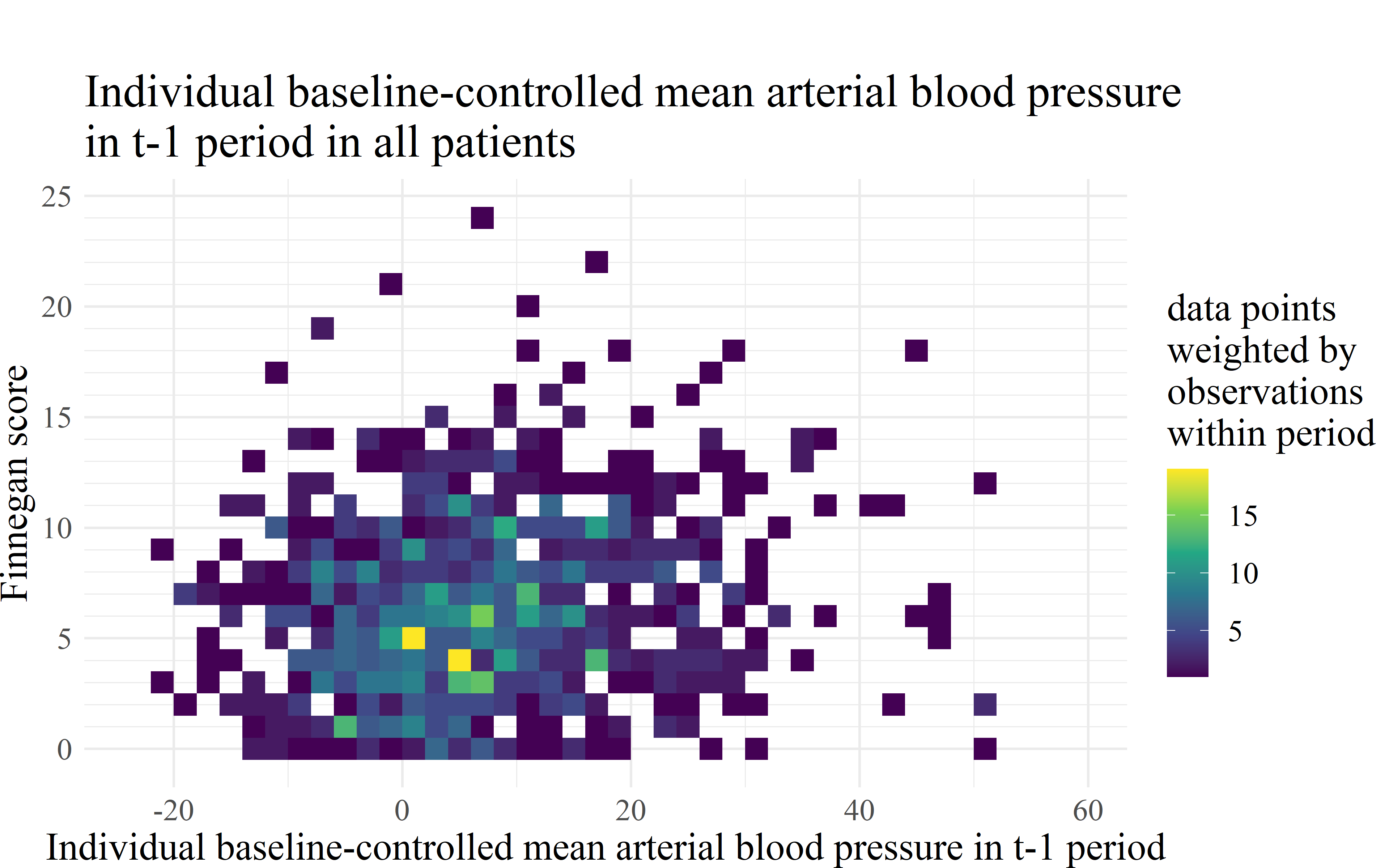

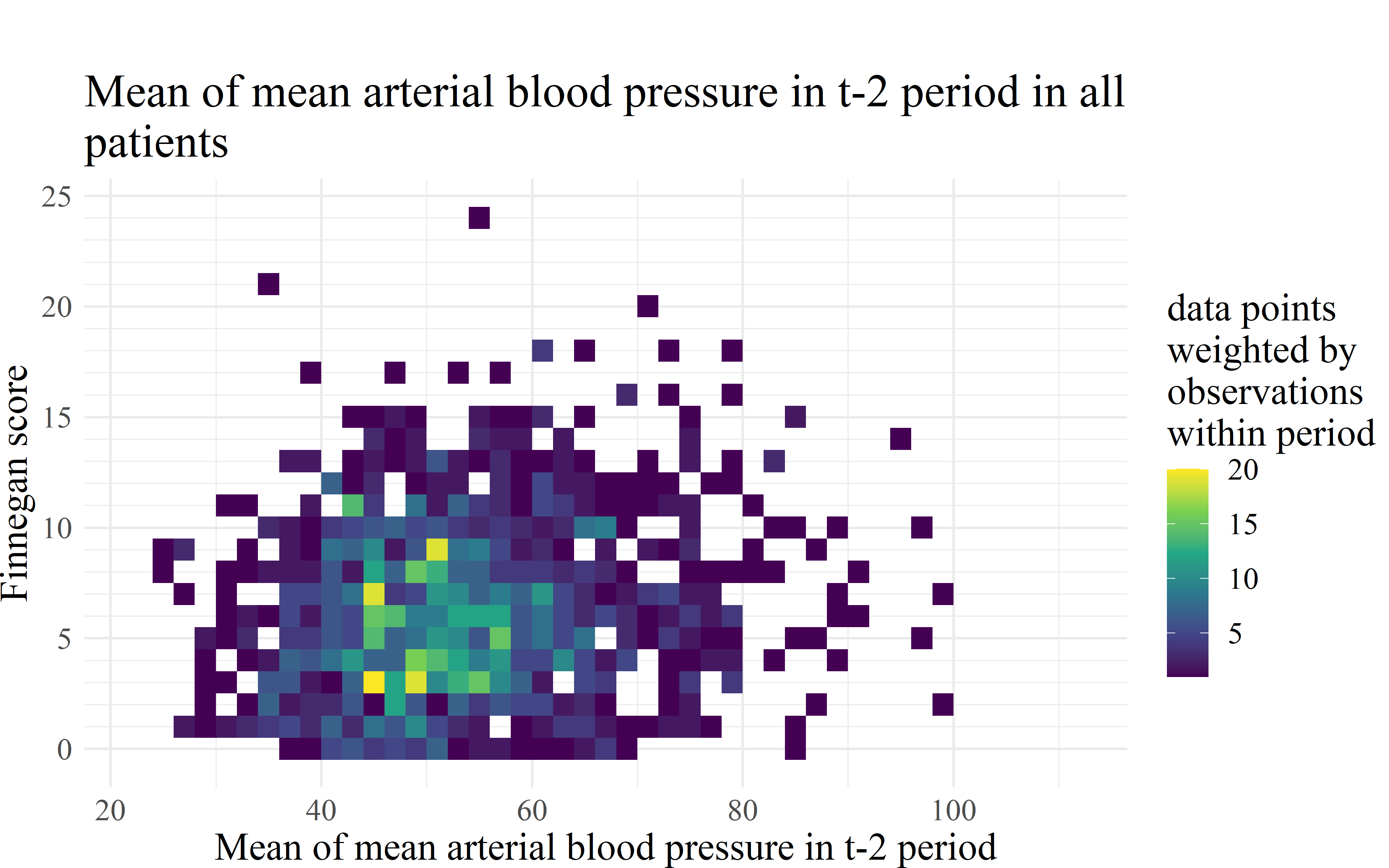

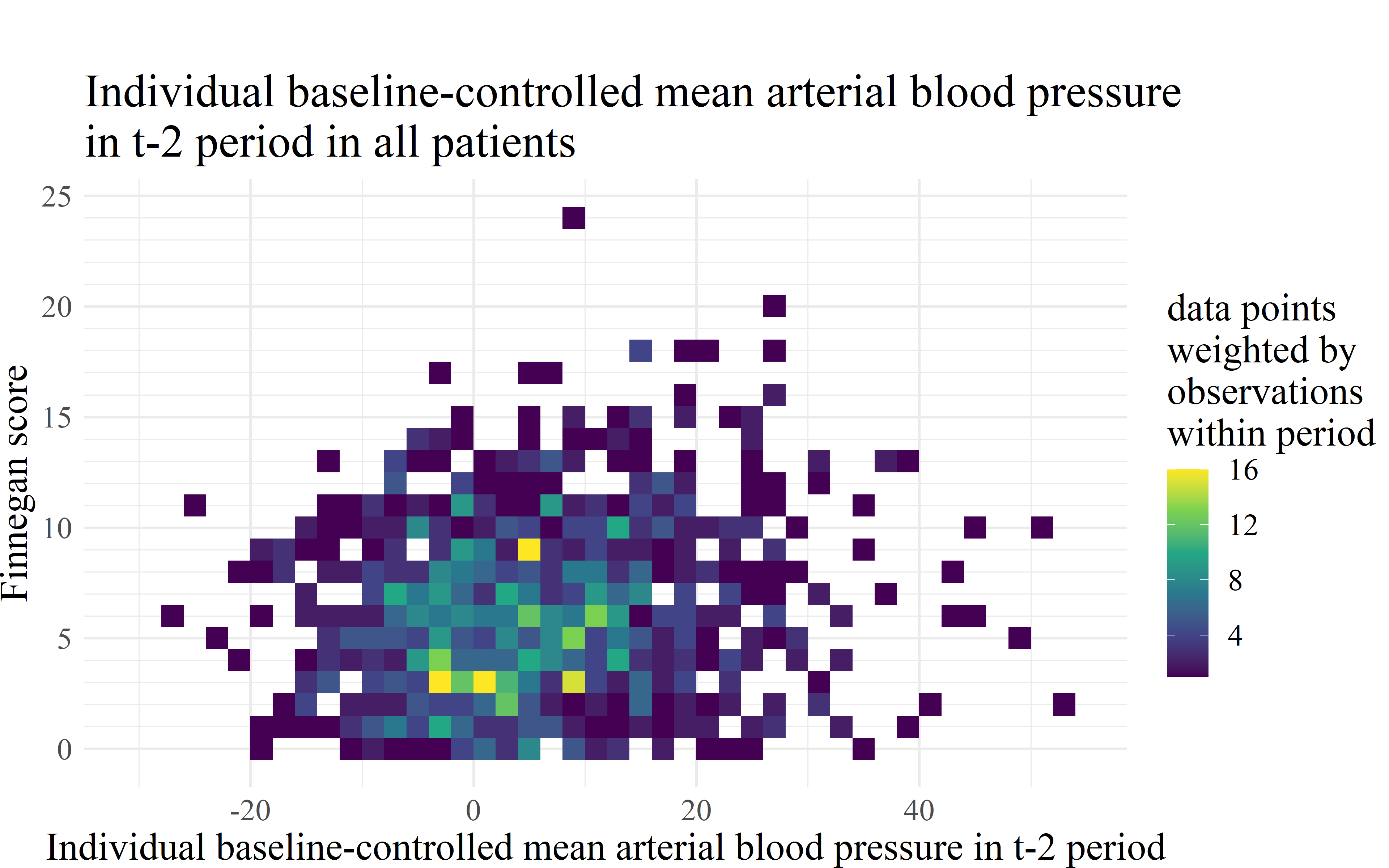


## Multimedia Appendix 2.2: Mixed effects models with full set of variables

### Variable set: Mean

#### t-1

| Table S2: Estimates for Mixed effects model on FS for t-1 using mean; full set of variables | | | | |
| --- | --- | --- | --- | --- |
| formula : Value of Finnegan-Score ~ Mean heart rate in t-1 period + Mean peripheral oxygen saturation in t-1 period + Mean respiratory rate in t-1 period + Mean of mean arterial blood pressure in t-1 period + Mean body temperature within 1 day before Finnegan-Score + Hours between last medication as specified and Finnegan-Score + Percentage of birth weight + Gestational age + ( 1 \| newid ) | | | | |
|  | Estimate | Std. Error | 95%-CI | p-value |
| (Intercept) | -22.88 | 11.16 | [-45.12, -1.25] | 0.041 |
| Mean heart rate in t-1 period | 0.03 | 0.01 | [0.01, 0.05] | 0.000 |
| Mean peripheral oxygen saturation in t-1 period | -0.01 | 0.03 | [-0.07, 0.04] | 0.614 |
| Mean respiratory rate in t-1 period | 0.01 | 0.01 | [0.00, 0.03] | 0.127 |
| Mean of mean arterial blood pressure in t-1 period | 0.05 | 0.01 | [0.03, 0.07] | 0.000 |
| Mean body temperature within 1 day before Finnegan-Score | 0.58 | 0.29 | [0.01, 1.16] | 0.049 |
| Hours between last medication as specified and Finnegan-Score | 0.00 | 0.00 | [0.00, 0.00] | 0.422 |
| Percentage of birth weight | 0.00 | 0.00 | [0.00, 0.00] | 0.313 |
| Gestational age | 0.06 | 0.04 | [-0.02, 0.13] | 0.157 |

| Table S3: Goodness to fit for Mixed effects model on FS for t-1 using mean; full set of variables | |
| --- | --- |
| R2m | 0.070 |
| R2c | 0.220 |
| AIC | 4,399.359 |
| BIC | 4,450.890 |
| Patients | 162.000 |
| Finnegan Scores | 800.000 |

#### t-2

| Table S4: Estimates for Mixed effects model on FS for t-2 using mean; full set of variables | | | | |
| --- | --- | --- | --- | --- |
| formula : Value of Finnegan-Score ~ Mean heart rate in t-2 period + Mean peripheral oxygen saturation in t-2 period + Mean respiratory rate in t-2 period + Mean of mean arterial blood pressure in t-2 period + Mean body temperature within 1 day before Finnegan-Score + Hours between last medication as specified and Finnegan-Score + Percentage of birth weight + Gestational age + ( 1 \| newid ) | | | | |
|  | Estimate | Std. Error | 95%-CI | p-value |
| (Intercept) | -36.31 | 12.97 | [-61.95, -11.2] | 0.005 |
| Mean heart rate in t-2 period | 0.02 | 0.01 | [0.01, 0.04] | 0.006 |
| Mean peripheral oxygen saturation in t-2 period | 0.02 | 0.02 | [-0.03, 0.07] | 0.432 |
| Mean respiratory rate in t-2 period | 0.02 | 0.01 | [0.00, 0.03] | 0.082 |
| Mean of mean arterial blood pressure in t-2 period | 0.05 | 0.01 | [0.03, 0.07] | 0.000 |
| Mean body temperature within 1 day before Finnegan-Score | 0.85 | 0.35 | [0.18, 1.54] | 0.014 |
| Hours between last medication as specified and Finnegan-Score | 0.00 | 0.00 | [0.00, 0.00] | 0.406 |
| Percentage of birth weight | 0.00 | 0.00 | [-0.01, 0.00] | 0.075 |
| Gestational age | 0.09 | 0.04 | [0.01, 0.17] | 0.027 |

| Table S5: Goodness to fit for Mixed effects model on FS for t-2 using mean; full set of variables | |
| --- | --- |
| R2m | 0.080 |
| R2c | 0.250 |
| AIC | 4,350.899 |
| BIC | 4,402.319 |
| Patients | 162.000 |
| Finnegan Scores | 792.000 |

###

### Variable set: Individual baseline-controlled mean

#### t-1

| Table S6: Estimates for Mixed effects model on FS for t-1 using baseline-controlled mean calculated with spacer period length of one day; full set of variables | | | | |
| --- | --- | --- | --- | --- |
| formula : Value of Finnegan-Score ~ Individual baseline-controlled heart rate in t-1 period + Individual baseline-controlled peripheral oxygen saturation in t-1 period + Individual baseline-controlled respiratory rate in t-1 period + Individual baseline-controlled mean arterial blood pressure in t-1 period + Individual baseline-controlled body temperature within 1 day before Finnegan-Score + Hours between last medication as specified and Finnegan-Score + Percentage of birth weight + Gestational age + ( 1 \| newid ) | | | | |
|  | Estimate | Std. Error | 95%-CI | p-value |
| (Intercept) | 2.22 | 1.94 | [-1.55, 6.08] | 0.252 |
| Individual baseline-controlled heart rate in t-1 period | 0.03 | 0.01 | [0.01, 0.05] | 0.001 |
| Individual baseline-controlled peripheral oxygen saturation in t-1 period | -0.07 | 0.04 | [-0.15, 0.00] | 0.063 |
| Individual baseline-controlled respiratory rate in t-1 period | 0.00 | 0.01 | [-0.01, 0.02] | 0.657 |
| Individual baseline-controlled mean arterial blood pressure in t-1 period | 0.04 | 0.01 | [0.02, 0.07] | 0.003 |
| Individual baseline-controlled body temperature within 1 day before Finnegan-Score | 0.00 | 0.32 | [-0.63, 0.63] | 0.989 |
| Hours between last medication as specified and Finnegan-Score | 0.00 | 0.00 | [0.00, 0.00] | 0.313 |
| Percentage of birth weight | 0.00 | 0.00 | [0.00, 0.00] | 0.692 |
| Gestational age | 0.11 | 0.05 | [0.01, 0.19] | 0.023 |

| Table S7: Goodness to fit for Mixed effects model on FS for t-1 using baseline-controlled mean; full set of variables | |
| --- | --- |
| R2m | 0.070 |
| R2c | 0.230 |
| AIC | 3,449.673 |
| BIC | 3,498.347 |
| Patients | 132.000 |
| Finnegan Scores | 617.000 |

#### t-2

| Table S8: Estimates for Mixed effects model on FS for t-2 using baseline-controlled mean calculated with spacer period length of one day; full set of variables | | | | |
| --- | --- | --- | --- | --- |
| formula : Value of Finnegan-Score ~ Individual baseline-controlled heart rate in t-2 period + Individual baseline-controlled peripheral oxygen saturation in t-2 period + Individual baseline-controlled respiratory rate in t-2 period + Individual baseline-controlled mean arterial blood pressure in t-2 period + Individual baseline-controlled body temperature within 1 day before Finnegan-Score + Hours between last medication as specified and Finnegan-Score + Percentage of birth weight + Gestational age + ( 1 \| newid ) | | | | |
|  | Estimate | Std. Error | 95%-CI | p-value |
| (Intercept) | 1.80 | 2.02 | [-2.14, 5.79] | 0.375 |
| Individual baseline-controlled heart rate in t-2 period | 0.03 | 0.01 | [0.01, 0.04] | 0.003 |
| Individual baseline-controlled peripheral oxygen saturation in t-2 period | 0.00 | 0.03 | [-0.07, 0.06] | 0.939 |
| Individual baseline-controlled respiratory rate in t-2 period | 0.00 | 0.01 | [-0.02, 0.02] | 0.889 |
| Individual baseline-controlled mean arterial blood pressure in t-2 period | 0.05 | 0.01 | [0.02, 0.08] | 0.000 |
| Individual baseline-controlled body temperature within 1 day before Finnegan-Score | -0.31 | 0.42 | [-1.13, 0.5] | 0.456 |
| Hours between last medication as specified and Finnegan-Score | 0.00 | 0.00 | [0.00, 0.00] | 0.795 |
| Percentage of birth weight | 0.00 | 0.00 | [-0.01, 0.00] | 0.277 |
| Gestational age | 0.12 | 0.05 | [0.03, 0.21] | 0.011 |

| Table S9: Goodness to fit for Mixed effects model on FS for t-2 using baseline-controlled mean; full set of variables | |
| --- | --- |
| R2m | 0.080 |
| R2c | 0.260 |
| AIC | 3,423.502 |
| BIC | 3,472.121 |
| Patients | 128.000 |
| Finnegan Scores | 614.000 |

## Multimedia Appendix 2.3: Mixed effects model without body temperature

### Variable set: Mean

#### t-1

| Table S10: Estimates for Mixed effects model on FS for t-1 using mean; body temperature removed from set of variables | | | | |
| --- | --- | --- | --- | --- |
| formula : Value of Finnegan-Score ~ Mean heart rate in t-1 period + Mean peripheral oxygen saturation in t-1 period + Mean respiratory rate in t-1 period + Mean of mean arterial blood pressure in t-1 period + Hours between last medication as specified and Finnegan-Score + Percentage of birth weight + Gestational age + ( 1 \| newid ) | | | | |
|  | Estimate | Std. Error | 95%-CI | p-value |
| (Intercept) | -1.85 | 3.41 | [-8.55, 4.76] | 0.587 |
| Mean heart rate in t-1 period | 0.03 | 0.01 | [0.02, 0.05] | 0.000 |
| Mean peripheral oxygen saturation in t-1 period | -0.02 | 0.03 | [-0.07, 0.04] | 0.535 |
| Mean respiratory rate in t-1 period | 0.01 | 0.01 | [0.00, 0.03] | 0.132 |
| Mean of mean arterial blood pressure in t-1 period | 0.05 | 0.01 | [0.03, 0.07] | 0.000 |
| Hours between last medication as specified and Finnegan-Score | 0.00 | 0.00 | [0.00, 0.00] | 0.532 |
| Percentage of birth weight | 0.00 | 0.00 | [0.00, 0.00] | 0.347 |
| Gestational age | 0.06 | 0.04 | [-0.01, 0.14] | 0.109 |

| Table S11: Goodness to fit for Mixed effects model on FS for t-1 using mean; body temperature removed from set of variables | |
| --- | --- |
| R2m | 0.070 |
| R2c | 0.230 |
| AIC | 4,406.121 |
| BIC | 4,452.980 |
| Patients | 162.000 |
| Finnegan Scores | 801.000 |

#### t-2

| Table S12: Estimates for Mixed effects model on FS for t-2 using mean; body temperature removed from set of variables | | | | |
| --- | --- | --- | --- | --- |
| formula : Value of Finnegan-Score ~ Mean heart rate in t-2 period + Mean peripheral oxygen saturation in t-2 period + Mean respiratory rate in t-2 period + Mean of mean arterial blood pressure in t-2 period + Hours between last medication as specified and Finnegan-Score + Percentage of birth weight + Gestational age + ( 1 \| newid ) | | | | |
|  | Estimate | Std. Error | 95%-CI | p-value |
| (Intercept) | -5.43 | 3.22 | [-11.68, 0.84] | 0.093 |
| Mean heart rate in t-2 period | 0.03 | 0.01 | [0.01, 0.04] | 0.002 |
| Mean peripheral oxygen saturation in t-2 period | 0.02 | 0.02 | [-0.03, 0.07] | 0.491 |
| Mean respiratory rate in t-2 period | 0.01 | 0.01 | [0.00, 0.03] | 0.104 |
| Mean of mean arterial blood pressure in t-2 period | 0.05 | 0.01 | [0.03, 0.08] | 0.000 |
| Hours between last medication as specified and Finnegan-Score | 0.00 | 0.00 | [0.00, 0.00] | 0.278 |
| Percentage of birth weight | 0.00 | 0.00 | [-0.01, 0.00] | 0.112 |
| Gestational age | 0.10 | 0.04 | [0.02, 0.18] | 0.017 |

| Table S13: Goodness to fit for Mixed effects model on FS for t-2 using mean; body temperature removed from set of variables | |
| --- | --- |
| R2m | 0.080 |
| R2c | 0.270 |
| AIC | 4,364.504 |
| BIC | 4,411.275 |
| Patients | 162.000 |
| Finnegan Scores | 794.000 |

### Variable set: Individual baseline-controlled mean

#### t-1

| Table S14: Estimates for Mixed effects model on FS for t-1 using baseline-controlled mean calculated with spacer period length of one day; body temperature removed from set of variables | | | | |
| --- | --- | --- | --- | --- |
| formula : Value of Finnegan-Score ~ Individual baseline-controlled heart rate in t-1 period + Individual baseline-controlled peripheral oxygen saturation in t-1 period + Individual baseline-controlled respiratory rate in t-1 period + Individual baseline-controlled mean arterial blood pressure in t-1 period + Hours between last medication as specified and Finnegan-Score + Percentage of birth weight + Gestational age + ( 1 \| newid ) | | | | |
|  | Estimate | Std. Error | 95%-CI | p-value |
| (Intercept) | 2.15 | 1.93 | [-1.63, 5.99] | 0.267 |
| Individual baseline-controlled heart rate in t-1 period | 0.03 | 0.01 | [0.01, 0.05] | 0.001 |
| Individual baseline-controlled peripheral oxygen saturation in t-1 period | -0.07 | 0.04 | [-0.15, 0.00] | 0.065 |
| Individual baseline-controlled respiratory rate in t-1 period | 0.00 | 0.01 | [-0.02, 0.02] | 0.730 |
| Individual baseline-controlled mean arterial blood pressure in t-1 period | 0.04 | 0.01 | [0.02, 0.07] | 0.003 |
| Hours between last medication as specified and Finnegan-Score | 0.00 | 0.00 | [0.00, 0.00] | 0.312 |
| Percentage of birth weight | 0.00 | 0.00 | [0.00, 0.00] | 0.707 |
| Gestational age | 0.11 | 0.05 | [0.02, 0.2] | 0.020 |

| Table S15: Goodness to fit for Mixed effects model on FS for t-1 using baseline-controlled mean; body temperature removed from set of variables | |
| --- | --- |
| R2m | 0.070 |
| R2c | 0.230 |
| AIC | 3,452.569 |
| BIC | 3,496.834 |
| Patients | 132.000 |
| Finnegan Scores | 618.000 |

#### t-2

| Table S16: Estimates for Mixed effects model on FS for t-2 using baseline-controlled mean calculated with spacer period length of one day; body temperature removed from set of variables | | | | |
| --- | --- | --- | --- | --- |
| formula : Value of Finnegan-Score ~ Individual baseline-controlled heart rate in t-2 period + Individual baseline-controlled peripheral oxygen saturation in t-2 period + Individual baseline-controlled respiratory rate in t-2 period + Individual baseline-controlled mean arterial blood pressure in t-2 period + Hours between last medication as specified and Finnegan-Score + Percentage of birth weight + Gestational age + ( 1 \| newid ) | | | | |
|  | Estimate | Std. Error | 95%-CI | p-value |
| (Intercept) | 1.71 | 2.02 | [-2.24, 5.69] | 0.399 |
| Individual baseline-controlled heart rate in t-2 period | 0.03 | 0.01 | [0.01, 0.04] | 0.004 |
| Individual baseline-controlled peripheral oxygen saturation in t-2 period | 0.00 | 0.03 | [-0.07, 0.06] | 0.949 |
| Individual baseline-controlled respiratory rate in t-2 period | 0.00 | 0.01 | [-0.02, 0.02] | 0.961 |
| Individual baseline-controlled mean arterial blood pressure in t-2 period | 0.05 | 0.01 | [0.02, 0.08] | 0.000 |
| Hours between last medication as specified and Finnegan-Score | 0.00 | 0.00 | [0.00, 0.00] | 0.821 |
| Percentage of birth weight | 0.00 | 0.00 | [-0.01, 0.00] | 0.302 |
| Gestational age | 0.12 | 0.05 | [0.03, 0.21] | 0.010 |

| Table S17: Goodness to fit for Mixed effects model on FS for t-2 using baseline-controlled mean; body temperature removed from set of variables | |
| --- | --- |
| R2m | 0.080 |
| R2c | 0.260 |
| AIC | 3,427.435 |
| BIC | 3,471.651 |
| Patients | 128.000 |
| Finnegan Scores | 615.000 |

## Multimedia Appendix 2.4: Mixed effects model without bloodpressure, peripheral_oxygen_saturation

### Variable set: Mean

#### t-1

| Table S18: Estimates for Mixed effects model on FS for t-1 using mean; blood pressure and peripheral oxygen saturation removed from set of variables | | | | |
| --- | --- | --- | --- | --- |
| formula : Value of Finnegan-Score ~ Mean heart rate in t-1 period + Mean respiratory rate in t-1 period + Mean body temperature within 1 day before Finnegan-Score + Hours between last medication as specified and Finnegan-Score + Percentage of birth weight + Gestational age + ( 1 \| newid ) | | | | |
|  | Estimate | Std. Error | 95%-CI | p-value |
| (Intercept) | -16.89 | 3.26 | [-23.33, -10.49] | 0.000 |
| Mean heart rate in t-1 period | 0.03 | 0.00 | [0.02, 0.03] | 0.000 |
| Mean respiratory rate in t-1 period | 0.02 | 0.00 | [0.01, 0.02] | 0.000 |
| Mean body temperature within 1 day before Finnegan-Score | 0.62 | 0.09 | [0.45, 0.79] | 0.000 |
| Hours between last medication as specified and Finnegan-Score | 0.00 | 0.00 | [0.00, 0.00] | 0.022 |
| Percentage of birth weight | 0.00 | 0.00 | [0.00, 0.00] | 0.002 |
| Gestational age | -0.10 | 0.01 | [-0.13, -0.08] | 0.000 |

| Table S19: Goodness to fit for Mixed effects model on FS for t-1 using mean; blood pressure and peripheral oxygen saturation removed from set of variables | |
| --- | --- |
| R2m | 0.06 |
| R2c | 0.28 |
| AIC | 61,874.11 |
| BIC | 61,940.51 |
| Patients | 327.00 |
| Finnegan Scores | 11,824.00 |

#### t-2

| Table S20: Estimates for Mixed effects model on FS for t-2 using mean; blood pressure and peripheral oxygen saturation removed from set of variables | | | | |
| --- | --- | --- | --- | --- |
| formula : Value of Finnegan-Score ~ Mean heart rate in t-2 period + Mean respiratory rate in t-2 period + Mean body temperature within 1 day before Finnegan-Score + Hours between last medication as specified and Finnegan-Score + Percentage of birth weight + Gestational age + ( 1 \| newid ) | | | | |
|  | Estimate | Std. Error | 95%-CI | p-value |
| (Intercept) | -17.70 | 3.31 | [-24.23, -11.21] | 0.000 |
| Mean heart rate in t-2 period | 0.02 | 0.00 | [0.02, 0.03] | 0.000 |
| Mean respiratory rate in t-2 period | 0.01 | 0.00 | [0.01, 0.02] | 0.000 |
| Mean body temperature within 1 day before Finnegan-Score | 0.66 | 0.09 | [0.49, 0.84] | 0.000 |
| Hours between last medication as specified and Finnegan-Score | 0.00 | 0.00 | [0.00, 0.00] | 0.037 |
| Percentage of birth weight | 0.00 | 0.00 | [0.00, 0.00] | 0.001 |
| Gestational age | -0.11 | 0.01 | [-0.14, -0.08] | 0.000 |

| Table S21: Goodness to fit for Mixed effects model on FS for t-2 using mean; blood pressure and peripheral oxygen saturation removed from set of variables | |
| --- | --- |
| R2m | 0.05 |
| R2c | 0.27 |
| AIC | 62,713.24 |
| BIC | 62,779.71 |
| Patients | 327.00 |
| Finnegan Scores | 11,919.00 |

### Variable set: Individual baseline-controlled mean

#### t-1

| Table S22: Estimates for Mixed effects model on FS for t-1 using baseline-controlled mean calculated with spacer period length of one day; blood pressure and peripheral oxygen saturation removed from set of variables | | | | |
| --- | --- | --- | --- | --- |
| formula : Value of Finnegan-Score ~ Individual baseline-controlled heart rate in t-1 period + Individual baseline-controlled respiratory rate in t-1 period + Individual baseline-controlled body temperature within 1 day before Finnegan-Score + Hours between last medication as specified and Finnegan-Score + Percentage of birth weight + Gestational age + ( 1 \| newid ) | | | | |
|  | Estimate | Std. Error | 95%-CI | p-value |
| (Intercept) | 8.21 | 0.62 | [6.96, 9.46] | 0.000 |
| Individual baseline-controlled heart rate in t-1 period | 0.03 | 0.00 | [0.02, 0.03] | 0.000 |
| Individual baseline-controlled respiratory rate in t-1 period | 0.01 | 0.00 | [0.00, 0.01] | 0.000 |
| Individual baseline-controlled body temperature within 1 day before Finnegan-Score | 0.02 | 0.08 | [-0.15, 0.18] | 0.808 |
| Hours between last medication as specified and Finnegan-Score | 0.00 | 0.00 | [0.00, 0.00] | 0.000 |
| Percentage of birth weight | 0.00 | 0.00 | [0.00, 0.00] | 0.129 |
| Gestational age | -0.06 | 0.01 | [-0.09, -0.03] | 0.000 |

| Table S23: Goodness to fit for Mixed effects model on FS for t-1 using baseline-controlled mean; blood pressure and peripheral oxygen saturation removed from set of variables | |
| --- | --- |
| R2m | 0.05 |
| R2c | 0.25 |
| AIC | 41,877.55 |
| BIC | 41,940.51 |
| Patients | 251.00 |
| Finnegan Scores | 8,065.00 |

#### t-2

| Table S24: Estimates for Mixed effects model on FS for t-2 using baseline-controlled mean calculated with spacer period length of one day; blood pressure and peripheral oxygen saturation removed from set of variables | | | | |
| --- | --- | --- | --- | --- |
| formula : Value of Finnegan-Score ~ Individual baseline-controlled heart rate in t-2 period + Individual baseline-controlled respiratory rate in t-2 period + Individual baseline-controlled body temperature within 1 day before Finnegan-Score + Hours between last medication as specified and Finnegan-Score + Percentage of birth weight + Gestational age + ( 1 \| newid ) | | | | |
|  | Estimate | Std. Error | 95%-CI | p-value |
| (Intercept) | 8.46 | 0.62 | [7.2, 9.71] | 0.000 |
| Individual baseline-controlled heart rate in t-2 period | 0.02 | 0.00 | [0.02, 0.03] | 0.000 |
| Individual baseline-controlled respiratory rate in t-2 period | 0.01 | 0.00 | [0.00, 0.01] | 0.000 |
| Individual baseline-controlled body temperature within 1 day before Finnegan-Score | 0.05 | 0.08 | [-0.12, 0.21] | 0.564 |
| Hours between last medication as specified and Finnegan-Score | 0.00 | 0.00 | [0.00, 0.00] | 0.000 |
| Percentage of birth weight | 0.00 | 0.00 | [0.00, 0.00] | 0.069 |
| Gestational age | -0.06 | 0.01 | [-0.09, -0.03] | 0.000 |

| Table S25: Goodness to fit for Mixed effects model on FS for t-2 using baseline-controlled mean; blood pressure and peripheral oxygen saturation removed from set of variables | |
| --- | --- |
| R2m | 0.04 |
| R2c | 0.24 |
| AIC | 42,224.85 |
| BIC | 42,287.86 |
| Patients | 251.00 |
| Finnegan Scores | 8,111.00 |

## Multimedia Appendix 2.5: Mixed effects models without bloodpressure, peripheral_oxygen_saturation, gest_age, body temperature

### Variable set: Mean

#### t-1

| Table S26: Estimates for Mixed effects model on FS for t-1 using mean; blood pressure, peripheral oxygen saturation, gestational age and body temperature removed from set of variables | | | | |
| --- | --- | --- | --- | --- |
| formula : Value of Finnegan-Score ~ Mean heart rate in t-1 period + Mean respiratory rate in t-1 period + Hours between last medication as specified and Finnegan-Score + Percentage of birth weight + ( 1 \| newid ) | | | | |
|  | Estimate | Std. Error | 95%-CI | p-value |
| (Intercept) | 1.58 | 0.28 | [1.02, 2.13] | 0.000 |
| Mean heart rate in t-1 period | 0.03 | 0.00 | [0.03, 0.03] | 0.000 |
| Mean respiratory rate in t-1 period | 0.02 | 0.00 | [0.01, 0.02] | 0.000 |
| Hours between last medication as specified and Finnegan-Score | 0.00 | 0.00 | [0.00, 0.00] | 0.029 |
| Percentage of birth weight | 0.00 | 0.00 | [0.00, 0.00] | 0.036 |

| Table S27: Goodness to fit for Mixed effects model on FS for t-1 using mean; blood pressure, peripheral oxygen saturation, gestational age and body temperature removed from set of variables | |
| --- | --- |
| R2m | 0.03 |
| R2c | 0.24 |
| AIC | 64,088.06 |
| BIC | 64,139.95 |
| Patients | 334.00 |
| Finnegan Scores | 12,239.00 |

#### t-2

| Table S28: Estimates for Mixed effects model on FS for t-2 using mean; blood pressure, peripheral oxygen saturation, gestational age and body temperature removed from set of variables | | | | |
| --- | --- | --- | --- | --- |
| formula : Value of Finnegan-Score ~ Mean heart rate in t-2 period + Mean respiratory rate in t-2 period + Hours between last medication as specified and Finnegan-Score + Percentage of birth weight + ( 1 \| newid ) | | | | |
|  | Estimate | Std. Error | 95%-CI | p-value |
| (Intercept) | 2.18 | 0.29 | [1.61, 2.76] | 0.000 |
| Mean heart rate in t-2 period | 0.03 | 0.00 | [0.02, 0.03] | 0.000 |
| Mean respiratory rate in t-2 period | 0.01 | 0.00 | [0.01, 0.02] | 0.000 |
| Hours between last medication as specified and Finnegan-Score | 0.00 | 0.00 | [0.00, 0.00] | 0.055 |
| Percentage of birth weight | 0.00 | 0.00 | [0.00, 0.00] | 0.023 |

| Table S29: Goodness to fit for Mixed effects model on FS for t-2 using mean; blood pressure, peripheral oxygen saturation, gestational age and body temperature removed from set of variables | |
| --- | --- |
| R2m | 0.02 |
| R2c | 0.23 |
| AIC | 64,910.29 |
| BIC | 64,962.23 |
| Patients | 334.00 |
| Finnegan Scores | 12,331.00 |

### Variable set: Individual baseline-controlled mean

#### t-1

| Table S30: Estimates for Mixed effects model on FS for t-1 using baseline-controlled mean calculated with spacer period length of one day; blood pressure, peripheral oxygen saturation, gestational age and body temperature removed from set of variables | | | | |
| --- | --- | --- | --- | --- |
| formula : Value of Finnegan-Score ~ Individual baseline-controlled heart rate in t-1 period + Individual baseline-controlled respiratory rate in t-1 period + Hours between last medication as specified and Finnegan-Score + Percentage of birth weight + ( 1 \| newid ) | | | | |
|  | Estimate | Std. Error | 95%-CI | p-value |
| (Intercept) | 5.86 | 0.14 | [5.58, 6.13] | 0.000 |
| Individual baseline-controlled heart rate in t-1 period | 0.03 | 0.00 | [0.02, 0.03] | 0.000 |
| Individual baseline-controlled respiratory rate in t-1 period | 0.01 | 0.00 | [0.00, 0.01] | 0.000 |
| Hours between last medication as specified and Finnegan-Score | 0.00 | 0.00 | [0.00, 0.00] | 0.000 |
| Percentage of birth weight | 0.00 | 0.00 | [0.00, 0.00] | 0.472 |

| Table S31: Goodness to fit for Mixed effects model on FS for t-1 using baseline-controlled mean; blood pressure, peripheral oxygen saturation, gestational age and body temperature removed from set of variables | |
| --- | --- |
| R2m | 0.04 |
| R2c | 0.22 |
| AIC | 43,472.18 |
| BIC | 43,521.41 |
| Patients | 256.00 |
| Finnegan Scores | 8,378.00 |

#### t-2

| Table S32: Estimates for Mixed effects model on FS for t-2 using baseline-controlled mean calculated with spacer period length of one day; blood pressure, peripheral oxygen saturation, gestational age and body temperature removed from set of variables | | | | |
| --- | --- | --- | --- | --- |
| formula : Value of Finnegan-Score ~ Individual baseline-controlled heart rate in t-2 period + Individual baseline-controlled respiratory rate in t-2 period + Hours between last medication as specified and Finnegan-Score + Percentage of birth weight + ( 1 \| newid ) | | | | |
|  | Estimate | Std. Error | 95%-CI | p-value |
| (Intercept) | 5.91 | 0.14 | [5.64, 6.18] | 0.0 |
| Individual baseline-controlled heart rate in t-2 period | 0.03 | 0.00 | [0.02, 0.03] | 0.0 |
| Individual baseline-controlled respiratory rate in t-2 period | 0.01 | 0.00 | [0.00, 0.01] | 0.0 |
| Hours between last medication as specified and Finnegan-Score | 0.00 | 0.00 | [0.00, 0.00] | 0.0 |
| Percentage of birth weight | 0.00 | 0.00 | [0.00, 0.00] | 0.3 |

| Table S33: Goodness to fit for Mixed effects model on FS for t-2 using baseline-controlled mean; blood pressure, peripheral oxygen saturation, gestational age and body temperature removed from set of variables | |
| --- | --- |
| R2m | 0.03 |
| R2c | 0.21 |
| AIC | 43,808.58 |
| BIC | 43,857.85 |
| Patients | 256.00 |
| Finnegan Scores | 8,422.00 |

## Multimedia Appendix 2.6: Mixed effects models without bloodpressure, peripheral_oxygen_saturation, gestational age, body temperature, percentage of body weight

### Variable set: Mean

#### t-1

| Table S34: Estimates for Mixed effects model on FS for t-1 using mean; blood pressure, peripheral oxygen saturation, gestational age, body temperature and percentage of birth weight removed from set of variables | | | | |
| --- | --- | --- | --- | --- |
| formula : Value of Finnegan-Score ~ Mean heart rate in t-1 period + Mean respiratory rate in t-1 period + Hours between last medication as specified and Finnegan-Score + ( 1 \| newid ) | | | | |
|  | Estimate | Std. Error | 95%-CI | p-value |
| (Intercept) | 1.42 | 0.27 | [0.88, 1.96] | 0.000 |
| Mean heart rate in t-1 period | 0.03 | 0.00 | [0.03, 0.03] | 0.000 |
| Mean respiratory rate in t-1 period | 0.02 | 0.00 | [0.01, 0.02] | 0.000 |
| Hours between last medication as specified and Finnegan-Score | 0.00 | 0.00 | [0.00, 0.00] | 0.038 |

| Table S35: Goodness to fit for Mixed effects model on FS for t-1 using mean; blood pressure, peripheral oxygen saturation, gestational age, body temperature and percentage of birth weight removed from set of variables | |
| --- | --- |
| R2m | 0.03 |
| R2c | 0.25 |
| AIC | 64,677.50 |
| BIC | 64,722.03 |
| Patients | 336.00 |
| Finnegan Scores | 12,347.00 |

#### t-2

| Table S36: Estimates for Mixed effects model on FS for t-2 using mean; blood pressure, peripheral oxygen saturation, gestational age, body temperature and percentage of birth weight removed from set of variables | | | | |
| --- | --- | --- | --- | --- |
| formula : Value of Finnegan-Score ~ Mean heart rate in t-2 period + Mean respiratory rate in t-2 period + Hours between last medication as specified and Finnegan-Score + ( 1 \| newid ) | | | | |
|  | Estimate | Std. Error | 95%-CI | p-value |
| (Intercept) | 2.03 | 0.29 | [1.47, 2.59] | 0.000 |
| Mean heart rate in t-2 period | 0.03 | 0.00 | [0.02, 0.03] | 0.000 |
| Mean respiratory rate in t-2 period | 0.01 | 0.00 | [0.01, 0.02] | 0.000 |
| Hours between last medication as specified and Finnegan-Score | 0.00 | 0.00 | [0.00, 0.00] | 0.069 |

| Table S37: Goodness to fit for Mixed effects model on FS for t-2 using mean; blood pressure, peripheral oxygen saturation, gestational age, body temperature and percentage of birth weight removed from set of variables | |
| --- | --- |
| R2m | 0.02 |
| R2c | 0.24 |
| AIC | 65,491.09 |
| BIC | 65,535.66 |
| Patients | 336.00 |
| Finnegan Scores | 12,436.00 |

###

### Variable set: Individual baseline-controlled mean

#### t-1

| Table S38: Estimates for Mixed effects model on FS for t-1 using baseline-controlled mean calculated with spacer period length of one day; blood pressure, peripheral oxygen saturation, gestational age, body temperature and percentage of birth weight removed from set of variables | | | | |
| --- | --- | --- | --- | --- |
| formula : Value of Finnegan-Score ~ Individual baseline-controlled heart rate in t-1 period + Individual baseline-controlled respiratory rate in t-1 period + Hours between last medication as specified and Finnegan-Score + ( 1 \| newid ) | | | | |
|  | Estimate | Std. Error | 95%-CI | p-value |
| (Intercept) | 5.81 | 0.11 | [5.59, 6.03] | 0 |
| Individual baseline-controlled heart rate in t-1 period | 0.03 | 0.00 | [0.03, 0.03] | 0 |
| Individual baseline-controlled respiratory rate in t-1 period | 0.01 | 0.00 | [0.00, 0.01] | 0 |
| Hours between last medication as specified and Finnegan-Score | 0.00 | 0.00 | [0.00, 0.00] | 0 |

| Table S39: Goodness to fit for Mixed effects model on FS for t-1 using baseline-controlled mean; blood pressure, peripheral oxygen saturation, gestational age, body temperature and percentage of birth weight removed from set of variables | |
| --- | --- |
| R2m | 0.04 |
| R2c | 0.22 |
| AIC | 44,046.92 |
| BIC | 44,089.19 |
| Patients | 258.00 |
| Finnegan Scores | 8,483.00 |

#### t-2

| Table S40: Estimates for Mixed effects model on FS for t-2 using baseline-controlled mean calculated with spacer period length of one day; blood pressure, peripheral oxygen saturation, gestational age, body temperature and percentage of birth weight removed from set of variables | | | | |
| --- | --- | --- | --- | --- |
| formula : Value of Finnegan-Score ~ Individual baseline-controlled heart rate in t-2 period + Individual baseline-controlled respiratory rate in t-2 period + Hours between last medication as specified and Finnegan-Score + ( 1 \| newid ) | | | | |
|  | Estimate | Std. Error | 95%-CI | p-value |
| (Intercept) | 5.84 | 0.11 | [5.62, 6.06] | 0 |
| Individual baseline-controlled heart rate in t-2 period | 0.03 | 0.00 | [0.02, 0.03] | 0 |
| Individual baseline-controlled respiratory rate in t-2 period | 0.01 | 0.00 | [0.00, 0.01] | 0 |
| Hours between last medication as specified and Finnegan-Score | 0.00 | 0.00 | [0.00, 0.00] | 0 |

| Table S41: Goodness to fit for Mixed effects model on FS for t-2 using baseline-controlled mean; blood pressure, peripheral oxygen saturation, gestational age, body temperature and percentage of birth weight removed from set of variables | |
| --- | --- |
| R2m | 0.03 |
| R2c | 0.21 |
| AIC | 44,387.95 |
| BIC | 44,430.26 |
| Patients | 258.00 |
| Finnegan Scores | 8,527.00 |

##

## Multimedia Appendix 2.7: Model fit comparison including reworked analysis

| Table S42: Goodness to fit parameters, patient and score counts of mixed effects models | | | | | | |
| --- | --- | --- | --- | --- | --- | --- |
|  | R2m | R2c | AIC | BIC | Patients | Finnegan Scores |
| Mixed effects model on FS for t-1 using mean; full set of variables | 0.07 | 0.22 | 4,399.359 | 4,450.890 | 162 | 800 |
| Mixed effects model on FS for t-2 using mean; full set of variables | 0.08 | 0.25 | 4,350.899 | 4,402.319 | 162 | 792 |
| Mixed effects model on FS for t-1 using baseline-controlled mean; full set of variables | 0.07 | 0.23 | 3,449.673 | 3,498.347 | 132 | 617 |
| Mixed effects model on FS for t-2 using baseline-controlled mean; full set of variables | 0.08 | 0.26 | 3,423.502 | 3,472.121 | 128 | 614 |
| Mixed effects model on FS for t-1 using mean; body temperature removed from set of variables | 0.07 | 0.23 | 4,406.121 | 4,452.980 | 162 | 801 |
| Mixed effects model on FS for t-2 using mean; body temperature removed from set of variables | 0.08 | 0.27 | 4,364.504 | 4,411.275 | 162 | 794 |
| Mixed effects model on FS for t-1 using baseline-controlled mean; body temperature removed from set of variables | 0.07 | 0.23 | 3,452.569 | 3,496.834 | 132 | 618 |
| Mixed effects model on FS for t-2 using baseline-controlled mean; body temperature removed from set of variables.1 | 0.08 | 0.26 | 3,427.435 | 3,471.651 | 128 | 615 |
| Mixed effects model on FS for t-1 using mean; blood pressure and peripheral oxygen saturation removed from set of variables | 0.06 | 0.28 | 61,874.108 | 61,940.509 | 327 | 11,824 |
| Mixed effects model on FS for t-2 using mean; blood pressure and peripheral oxygen saturation removed from set of variables | 0.05 | 0.27 | 62,713.239 | 62,779.712 | 327 | 11,919 |
| Mixed effects model on FS for t-1 using baseline-controlled mean; blood pressure and peripheral oxygen saturation removed from set of variables | 0.05 | 0.25 | 41,877.551 | 41,940.509 | 251 | 8,065 |
| Mixed effects model on FS for t-2 using baseline-controlled mean; blood pressure and peripheral oxygen saturation removed from set of variables.1 | 0.04 | 0.24 | 42,224.854 | 42,287.863 | 251 | 8,111 |
| Mixed effects model on FS for t-1 using mean; blood pressure, peripheral oxygen saturation, gestational age and body temperature removed from set of variables | 0.03 | 0.24 | 64,088.060 | 64,139.946 | 334 | 12,239 |
| Mixed effects model on FS for t-2 using mean; blood pressure, peripheral oxygen saturation, gestational age and body temperature removed from set of variables | 0.02 | 0.23 | 64,910.287 | 64,962.226 | 334 | 12,331 |
| Mixed effects model on FS for t-1 using baseline-controlled mean; blood pressure, peripheral oxygen saturation, gestational age and body temperature removed from set of variables | 0.04 | 0.22 | 43,472.180 | 43,521.414 | 256 | 8,378 |
| Mixed effects model on FS for t-2 using baseline-controlled mean; blood pressure, peripheral oxygen saturation, gestational age and body temperature removed from set of variables | 0.03 | 0.21 | 43,808.583 | 43,857.853 | 256 | 8,422 |
| Mixed effects model on FS for t-1 using mean; blood pressure, peripheral oxygen saturation, gestational age, body temperature and percentage of birth weight removed from set of variables | 0.03 | 0.25 | 64,677.503 | 64,722.030 | 336 | 12,347 |
| Mixed effects model on FS for t-2 using mean; blood pressure, peripheral oxygen saturation, gestational age, body temperature and percentage of birth weight removed from set of variables | 0.02 | 0.24 | 65,491.088 | 65,535.659 | 336 | 12,436 |
| Mixed effects model on FS for t-1 using baseline-controlled mean; blood pressure, peripheral oxygen saturation, gestational age, body temperature and percentage of birth weight removed from set of variables | 0.04 | 0.22 | 44,046.916 | 44,089.191 | 258 | 8,483 |
| Mixed effects model on FS for t-2 using baseline-controlled mean; blood pressure, peripheral oxygen saturation, gestational age, body temperature and percentage of birth weight removed from set of variables. | 0.03 | 0.21 | 44,387.955 | 44,430.261 | 258 | 8,527 |
